# Bypassing the proline/thiazoline requirement of the macrocyclase PatG†
†Electronic supplementary information (ESI) available. See DOI: 10.1039/c7cc06550g


**DOI:** 10.1039/c7cc06550g

**Published:** 2017-11-01

**Authors:** E. Oueis, H. Stevenson, M. Jaspars, N. J. Westwood, J. H. Naismith

**Affiliations:** a Biomedical Science Research Complex & School of Chemistry , University of St Andrews , BSRC , North Haugh , St Andrews , KY16 9ST , UK . Email: naismith@strubi.ox.ac.uk; b Marine Biodiscovery Centre , Department of Chemistry , University of Aberdeen , Old Aberdeen , AB24 3UE , UK; c State Key Laboratory of Biotherapy , Sichuan University , China; d Division of Structural Biology , Oxford University , OX3 7BN , UK; e Research Complex at Harwell , Didicot, Oxon , OX11 0FA , UK

## Abstract

Biocatalysis is a fast developing field in which an enzyme's natural capabilities are harnessed or engineered for synthetic chemistry. The enzyme PatG is an extremely promiscuous macrocyclase enzyme tolerating both non-natural amino acids and non-amino acids within the substrate. It does, however, require a proline or thiazoline at the C-terminal position of the core peptide which means the final product must contain this group. Here, we show guided by structural insight we have identified two synthetic routes, triazole and a double cysteine, that circumvent this requirement. With the triazole, we show PatGmac can macrocyclise substrates that do not contain any amino acids in the final product.

## Abstract 2

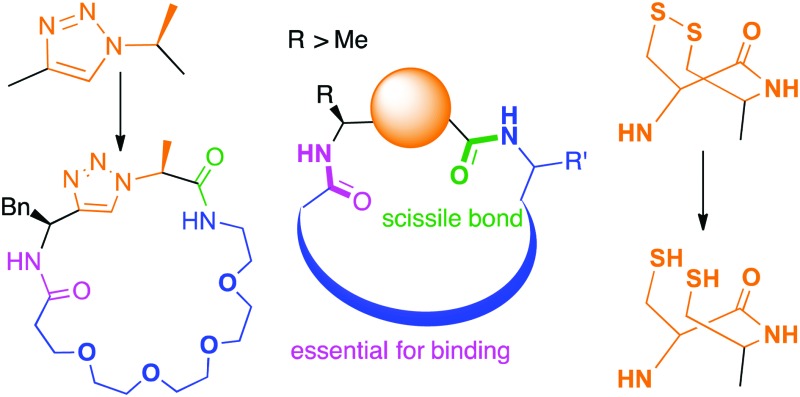
Macrocyclisation of fully non-peptidic compounds and non-heterocycle containing macrocycles using the peptidic ligase PatGmac.

## 

Enzymes provide useful tools as catalysts to achieve complex transformations in organic synthesis that either because of stereochemical variability or high activation energy are difficult to accomplish chemically. Concerns about environmental costs of organic solvents and waste streams have further driven the use of enzymes. Advances in recombinant DNA technology and directed evolution strategies have improved the availability, stability, and reactivity of enzymes.^1^ Innovations in protein immobilisation,^2^ microfluidic reactors,^3^ and protein design^4^ have further extended their utility. A wide range of transformations including hydrolytic reactions, reductive and oxidative reactions, transfer reactions, and carbon–carbon bond formation are catalysed by enzymes.^5^

Macrocyclisation is an important modification in the synthesis of many biologically active compounds, including not only natural products but also drug leads.^6^ It is argued that macrocyclic compounds (whether peptidic or not) are better drug leads for challenging molecular targets such as protein–protein interactions.^7^ Hence, the strong interest in these molecules as therapeutics.^8^ This has driven the development of a number of new technologies for the generation of diverse libraries of cyclic peptides *in vivo* (SICLOPPS: split-intein circular ligation of peptides and proteins)^9^ or the construction of non-standard peptide libraries *in vitro* (RaPID: random non-standard peptide integrated discovery)^10^ for example. Macrocyclic peptides are generally acknowledged as structurally diverse, rigid and stable (chemically and enzymatically), highly desirable properties for therapeutic applications, despite their size.^11^ Significant progress has been reported on predicting membrane permeability of large cyclic compounds that lie outside Lipinski's rule of 5, allowing for a more rational approach to macrocycle drug design.^12^ Several enzyme macrocyclases are known, and some have already been exploited for biocatalysis. Butelase 1 macrocyclises peptides and proteins (26–200 residues) at an extremely fast rate;^13^ PCY1 is a naturally occurring promiscuous macrocyclase for smaller peptides (5–9);^14^ PoPB macrocyclises the α-amanitin precursor peptide^15^ and PatGmac,^16^ is a highly promiscuous macrocyclase from the cyanobactins, a family of heterocycle-containing peptides.^17^ A principal limitation of these enzymes is that they operate on peptide substrates; yet a general enzyme catalysed synthesis of macrocycles is highly desirable.

PatGmac requires a C-terminal recognition sequence AYD (which is cleaved off during macrocyclisation) and either thiazoline or l-proline immediately preceding the recognition tag. The ring is thought to be essential as it adopts either a *cis*-(proline) or *cis*-like (thiazoline) conformation allowing the substrate (core) peptide to curve away from the enzyme.16a,b,d,e,17b Consequently, there are only a few interactions with the core peptide and very few restrictions on substrate. Those restrictions include no d-amino acids at either the N-terminus or either of the last two C-terminal positions of the core peptide.16a,e PatGmac has a broad substrate scope including non-natural amino acids,16*a* peptides with up to three 1,2,3-triazole rings,16*d* non-amino acidic scaffolds including sugars, benzene rings, alkyl and PEG chains16*e* (Fig. 1). Hybrid peptide non-peptide molecules with only three amino acids including the terminal l-Pro/thiazoline have been made.16*e* Structural biology has rationalised the requirement for this conformation-inducing terminal residue. The requirement does limit the scope of the enzyme and always results in a proline/thiazoline in the final product.

**Fig. 1:**
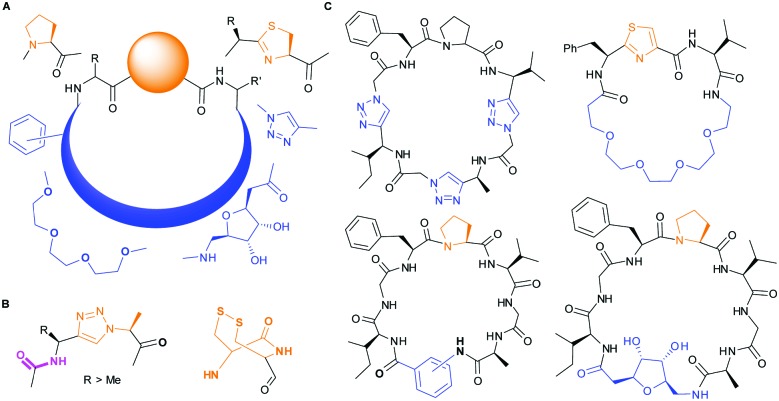
PatG substrates: (A) accepted substitutions within final product are shown in blue, but so far require proline or thiazoline (orange). (B) Triazole and double cysteine bypass this requirement. (C) Macrocycles synthesized with PatGmac so far.16d,e

Here we explored options to remove the requirement for a thiazoline/l-Pro residue at the C-terminus of the core peptide. We have used 1,4-*anti*-1,2,3-triazole-alanine^18^ and vicinal cysteine disulphide bonds^19^ as replacements for l-Pro. The former has allowed the synthesis of fully non-peptidic macrocycles using enzymes, whilst the latter allows for the generation of non-heterocycle containing macrocycles that can vary conformation in response to redox conditions.

From structural analysis, we hypothesised that one or both hydrogen bonds between the C-terminus of the core peptide in the substrate and the enzyme was critical for recognition (Fig. 2).17*b* We designed, synthesised and tested range of substrates with an insertion of a 1,4-disubstituted *anti*-triazole-alanine at the C-terminus of the core peptide (position 8) (Table 1). The 1,4-*anti*-triazole is easily obtained using Cu(i) catalysis and can be achieved on solid phase during peptide synthesis.16d,^20^ We first used propargylamine for the triazole (Tz**_1–4_**) formation, as this glycine mimic was commercially available. The alanine azido-acid counterpart was synthesised in one step by a diazo transfer reaction from commercial alanine^21^ Peptides **1–3** were synthesised with Gly–Tz**_1_**_–_**_4_**–Ala dipeptide mimic at the C-terminus (positions 7 and 8) but all failed to yield the desired product. Only unreacted starting peptide remained in solution, indicating the peptide was not a PatGmac substrate. To explore if the 1,4-*anti*-triazole precluded a *cis*-like conformation, the 1,5-disubstituted *syn*-triazole (Tz**_1–5_**) was employed with Gly–Tz**_1–5_**–Ala dipeptide mimic at positions 7 and 8 of the precursor peptide. Neither Ru(Cp*Cl(PPh_3_)_2_) nor RuCp*(cod)Cl catalysts^22^ gave a useful amount of fully protected dipeptide Gly–Tz**_1–5_**–Ala. The thermal reaction between Fmoc-protected propargylamine and the alanine azido benzylic ester afforded a 1 : 2 ratio of *syn*- to *anti*-triazole. The carboxylic ester of the triazole-containing dipeptides was then hydrolysed and the regioisomeric mixture was used as a building block in the peptide synthesis to generate peptides **2**/**4** and **3**/**5**. The regioisomeric triazole-containing peptides of both sequences were separated by HPLC but 1,5-*syn*-triazole peptides **4** and **5** gave the same negative result as their corresponding Tz**_1–4_** peptides (*cf.* peptides 2 and 4, 3 and 5 in Table 1). The structure of PatGmac H618A in complex with a proline-containing substrate (PDB ; 4AKT)17*b* reveals a binding pocket for the side chain of position 7 (Fig. 2). We hypothesised that a side chain at position 7 might be necessary to rigidify the peptide and thus favour the hydrogen bond. In order to test this hypothesis, peptides **6** and **7** with a proline preceded by a glycine and alanine (phenylalanine **8** at this position has been previously shown to be a substrate)16*d* were tested. Peptide **6** (Gly) was unchanged by the enzyme but peptide **7** (Ala) was processed to afford cyclic peptide, at essentially the same rate and efficiency as PatGmac peptide substrates. We concluded a side chain at position 7 is required to properly orient substrate to make the second hydrogen bond. The requirement for both hydrogen bonds also rationalises why peptides containing β-alanine (**9**) or (*R*)-3-amino-2-methylpropanoic acid ((*R*)-β^2^-homoalanine) (**10**), which move the amide out of position due to an extra carbon, were not processed by the enzyme.

**Table 1:** Synthetic peptides **1**–**26**, the resulting PatGmac reaction outcome, and isolated macrocyclic peptides and their yields

Peptide	Substrate sequence^*a*^	Product^*b*^	Cyclic^*c*^	Yield^*d*^ (%)
**1**	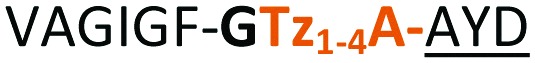	NR		
**2**	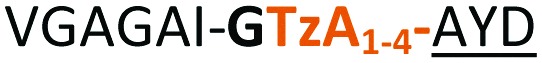	NR		
**3**	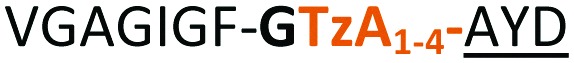	NR		
**4**	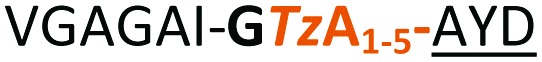	NR		
**5**	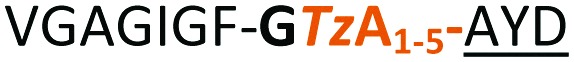	NR		
**6**	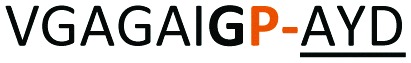	NR		
**7**	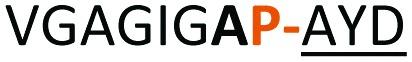	**C**		
**8**	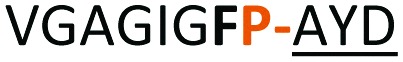	**C**	**8c** 16*d*	32
**9**	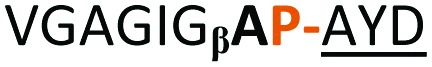	NR		
**10**	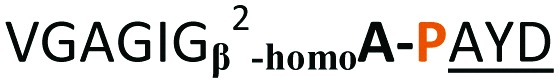	NR		
**11a**	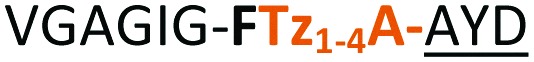	**C**	**11c/d**	40
**11b**		NR		
**12**	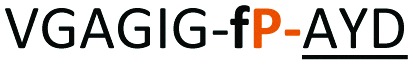	NR		
**13a**	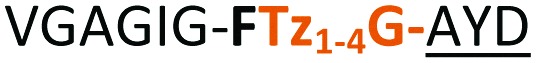	NR		
**13b**		NR		
**14**	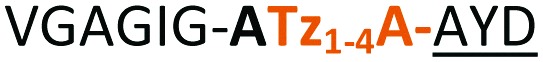	**C**	**14c**	40
**15**	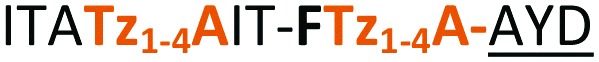	**C**		
**16**	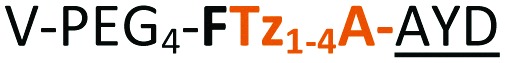	**C**	**16c/d**	36
**17**	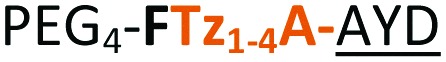	**C**	**17c**	34
**18**	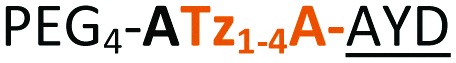	**C**		
**19**	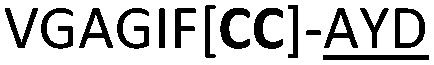	**C** (L)	**19c**	37
**20**	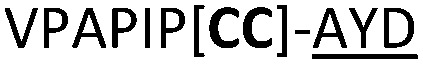	L		
**21**	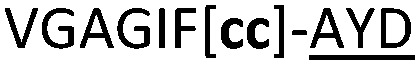	NR		
**22**	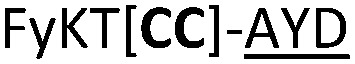	L		
**23**	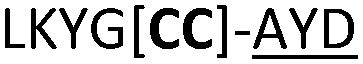	NR		
**24**	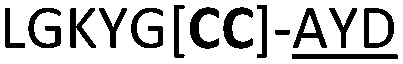	**C**		
**25**	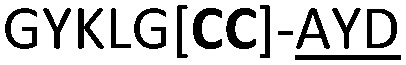	**C** (L)		
**26**	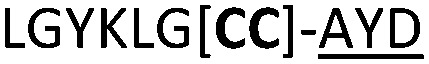	**C** (L)		

^*a*^Underlined is the minimal recognition sequence AYD where the peptide is cleaved. Tz**_1–4_** = 1,4-*anti*-triazole, Tz**_1–5_**= 1,5-*syn*-triazole, βA = β-alanine, β^2^-homoA = (*R*)-3-amino-2-methylpropanoic acid, d-amino acids in lower case, [CC] = disulphide bond.

^*b*^NR = No reaction, C = Cyclic, L = Linear; major product shown in table, minor product shown in brackets.

^*c*^Isolated macrocycles.

^*d*^Their yields.

**Fig. 2:**
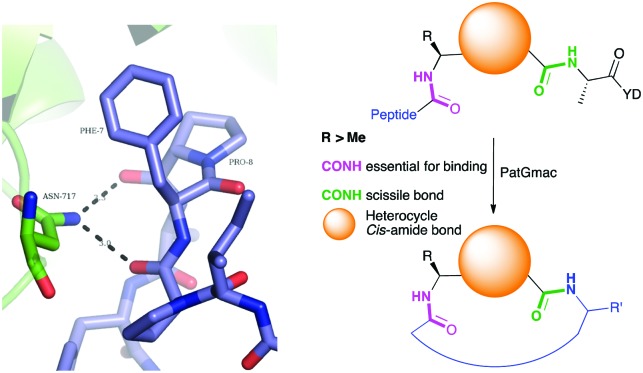
Left: Hydrogen bonding between the side chain of Asn717 and the carbonyls of substrate. We conclude these hydrogen bonds are critical in substrate recognition and explain the requirement for an l-configured non-glycine residue at position residues 7 of substrate (Phe is shown from PDB ; 4AKT). Right: PatGmac macrocyclisation substrate requirements: a heterocycle before AYD adopting a *cis*- or *cis*-like conformation, a side chain (R ≥ Me) preceding the heterocycle. We conclude two substrate carbonyls (coloured green & pink) hydrogen bonded with Asn717 are critical.

Having established the importance of position 7, we modified our design strategy. We synthesized peptide **11** with Phe–Tz**_1–4_**–Ala dipeptide mimic at the C-terminus. The phenylalanine amino alkyne derivative was synthesised in five steps starting from the commercial Boc protected amino acid, as previously described.16*d* Epimerisation of phenylalanine amino alkyne occurred for the phenylalanine derivative during synthesis affording a mixture of inseparable enantiomers. The final diastereomeric peptides however were easily separated by HPLC affording peptides **11a** and **11b**. The major diastereomer **11a** was macrocyclised by PatGmac, whereas the minor diastereomer **11b** did not. Since d-amino acids at positions 7 (the control peptide **12** with a d-Phe at position 7 failed to give the corresponding macrocycle) and d amino acids at position **8** have been reported not to be tolerated,16*a***11b** was assigned as having one d-amino acid at the triazole, either position 7 or 8 and does not process. The macrocyclisation of peptide **11a** demonstrates for the first time that the l-Pro requirement can be dispensed with and consistent with knowledge of the system is assumed to be l-Phe–Tz**_1–4_**–l-Ala. Deletion of the methyl group to afford Phe–Tz**_1–4_**–Gly dipeptide mimic (peptides **13a, b**) afforded no product in the presence of PatGmac, as expected. The dipeptide mimic Ala–Tz**_1–4_**–Ala **14** was a substrate as was peptide **15**, which has two triazole units. This led us to conclude that replacing l-Pro with the triazole group did not diminish our ability to introduce other modifications elsewhere in the core peptide. In order to explore these limits, peptide **16** having one amino acid at the N-terminus linked by a PEG chain was tested and macrocyclised. Peptides **17** and **18**, which have no α amino acids in the core, but have a free amine on a PEG group, gave macrocyclic compounds.

We then explored the use of vicinal l-cysteine disulphide bond as an alternative to proline, a defining motif of three important cyclic peptide families that are biologically active: malformins, somatostatins, and conotoxins.^23^ The disulfide bond containing substrate peptides (**19**–**26**) were synthesised by standard solid phase peptide synthesis and isolated as free cysteine-containing peptides and then oxidised overnight in 10% DMSO in TFA. Peptides **19** and **20** were designed as a positive and negative controls as **19** mimics **8**, a good substrate, whilst **20** mimics VPAPIPFP a poor substrate that mainly gives linear peptide.17*b* Analysis after two days of incubation showed that peptide **19** gave mainly macrocyclic peptide whereas peptide **20** gave only cleaved linear peptide. Peptide **21** with the d-cysteine motif (akin to malformins)^23^ did not macrocyclise. We took these results as confirming that vicinal l-cysteine disulphide bond could replace l-Pro and we explored derivatives of the remaining natural product families with this motif. Cyclic hexapeptidic analogues of somatostatin-containing YwKT or FwKT and a vicinal cysteine disulphide bond were reported to be more potent than the natural product.^23^ Peptide **22** was designed to produce such an analogue, but mainly yielded cleaved peptide, most likely because of the PatGmac preference for longer peptides.16*e* As a final test we explored conotoxin cyclic analogues^24^**23**–**26**. The hexapeptide **23** did not react; however the other hepta- and octa-peptide substrates all gave the desired cyclic peptides. PatGmac does afford facile access to analogues of conotoxin. We confirmed that reduction of the disulfide bond of macrocyle **19c** by TCEP in methanol/water was possible after macrocyclisation to give peptide **19d** (ESI†). This result opens the possibility of redox control of the macrocycle structure.

Products were identified by MALDI-MS and further verified by MS–MS fragmentation data (ESI†). Where the reactions were carried out at scale, macrocycles (Fig. 3) were isolated, purified by HPLC, and characterised using HRMS and NMR (ESI†). We observed moderate yields (32–40%) with an incubation of two weeks at 37 °C and pH = 8.1. Cyclic peptides derived from **11a** and **16** purified as two HPLC separable peaks with identical chemical composition determined by NMR and HRMS. EXSY NMR analysis indicates that they are not readily exchangeable conformers, suggesting these are either diasteriomers or structurally rigid conformers unable to interconvert (atropisomers). Spontaneous epimerization of macrocycles is known,^25^ but seems less likely for alpha position of a triazole. PatGmac does not process peptide substrates with d-amino acids at either position 7 or 8, it maybe the triazole leads to PatGmac tolerating substrate d-configuration. Cyclic peptide **17c** purified as a single peak, but proton NMR identified a minor (<5%) conformer. Macrocycle **14c** has in addition to the predominant conformer other minor conformers. Macrocycle **19c** gave rise to a complex spectrum, showing one major compound with at least two other minor conformers that were not fully identified.

**Fig. 3:**
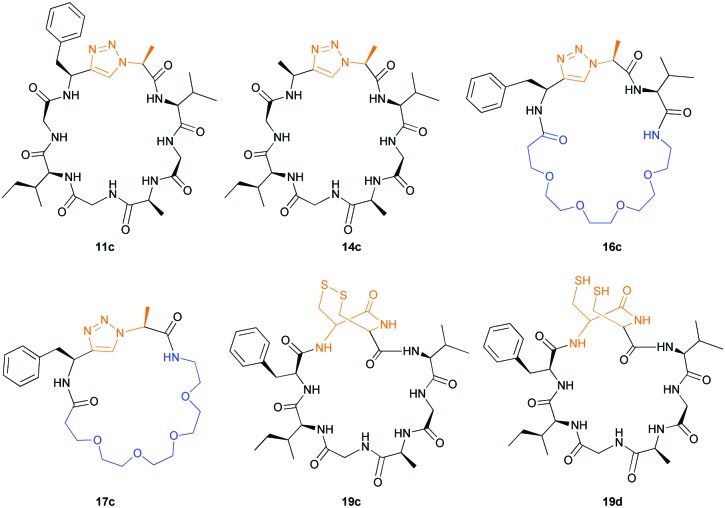
Non-proline containing macrocycles macrocyclised by PatGmac.

Macrocyclisation is an important and essential transformation in nature in general, and for the synthesis of biologically active molecules. Many enzymes in nature are involved in ring closure reactions, but are usually restricted to their biosynthetic pathway. Indeed, biocatalysis in general can be very efficient in conducting certain reactions, however this usually comes at a very high price in terms of substrate specificity. Hence, the ability to macrocyclise a range of structurally different compounds is a huge advantage allowing for a wider diversity. Herein we show that PatGmac is able to macrocyclise cyclic peptides containing non-natural and natural proline mimics at the C-terminus of the core peptide, a position that was thought to be restricted to natural heterocycles. We were able to synthesize macrocyclic compounds with no amino acids or no heterocycles. This very broad substrate range of PatGmac expands the scope of its applications, despite its slower rate and moderate yields. Nonetheless, further improvement of the catalytic efficiency of the enzyme achieved by directed evolution^26^ or protein engineering would be valuable. To the best of our knowledge, PatGmac is the first described peptidic ligase capable of macrocyclising non-peptidic precursors, making it the first enzymatic tool employed for the generation of diverse macrocyclic libraries.

This work was supported by the European Research Council (339367), UK Biotechnology and Biological Sciences Research Council (K015508/1), the Wellcome Trust (TripleTOF 5600 mass spectrometer (094476), the MALDI TOF–TOF Analyser (079272AIA), 700 NMR) and the EPSRC UK National Mass Spectrometry Facility at Swansea University. J. H. N. is a Royal Society Wolfson Merit Award Holder and 1000 talent scholar at Sichuan University.

## Conflicts of interest

There are no conflicts to declare.

## Supplementary Material

Supplementary information

## References

[cit1] Reetz M. T. (2013). J. Am. Chem. Soc..

[cit2] Cynthia S., Shelley D. M. (2008). Recent Pat. Eng..

[cit3] Laurenti E., Santos Vianna Jr. A. dos (2016). Biocatalysis.

[cit4] Li Y., Cirino P. C., Bornscheuer U. T. (2014). Biotechnol. Bioeng..

[cit5] Clouthier C. M., Pelletier J. N. (2012). Chem. Soc. Rev..

[cit6] Bockus A. T., McEwen C. M., Lokey R. S., Wessjohann L. A., Ruijter E., Garcia-Rivera D., Brandt W., Yu X., Sun D., Yudin A. K. (2013). Curr. Top. Med. Chem..

[cit7] Cardote T. A. F., Ciulli A., Corte J. R., Fang T., Osuna H., Pinto D. J. P., Rossi K. A., Myers J. E., Sheriff S., Lou Z., Zheng J. J., Harper T. W., Bozarth J. M., Wu Y., Luettgen J. M., Seiffert D. A., Decicco C. P., Wexler R. R., Quan M. L. (2016). ChemMedChem.

[cit8] Driggers E. M., Hale S. P., Lee J., Terrett N. K. (2008). Nat. Rev. Drug Discovery.

[cit9] Tavassoli A. (2017). Curr. Opin. Chem. Biol..

[cit10] Passioura T., Suga H. (2017). Chem. Commun..

[cit11] Newman D. J. and Cragg G. M., Macrocycles in Drug Discovery, The Royal Society of Chemistry, 2015.

[cit12] Giordanetto F., Kihlberg J., Over B., Matsson P., Tyrchan C., Artursson P., Doak B. C., Foley M. A., Hilgendorf C., Johnston S. E., Iv M. D. Lee, Lewis R. J., McCarren P., Muncipinto G., Norinder U., Perry M. W. D., Duvall J. R., Kihlberg J., Pye C. R., Hewitt W. M., Schwochert J., Haddad T. D., Townsend C. E., Etienne L., Lao Y., Limberakis C., Furukawa A., Mathiowetz A. M., Price D. A., Liras S., Lokey R. S. (2014). J. Med. Chem..

[cit13] Nguyen G. K. T., Kam A., Loo S., Jansson A. E., Pan L. X., Tam J. P. (2015). J. Am. Chem. Soc..

[cit14] Chekan J. R., Estrada P., Covello P. S., Nair S. K., Barber C. J., Pujara P. T., Reed D. W., Chiwocha S., Zhang H., Covello P. S. (2017). Proc. Natl. Acad. Sci. U. S. A..

[cit15] Czekster C. M., Naismith J. H., Luo H., Hong S.-Y., Sgambelluri R. M., Angelos E., Li X., Walton J. D. (2017). Biochemistry.

[cit16] McIntosh J. A., Robertson C. R., Agarwal V., Nair S. K., Bulaj G. W., Schmidt E. W. (2010). J. Am. Chem. Soc..

[cit17] Schmidt E. W., Nelson J. T., Rasko D. A., Sudek S., Eisen J. A., Haygood M. G., Ravel J. (2005). Proc. Natl. Acad. Sci. U. S. A..

[cit18] Tam A., Arnold U., Soellner M. B., Raines R. T. (2007). J. Am. Chem. Soc..

[cit19] Etzkorn F. A., Advances in Amino Acid Mimetics and Peptidomimetics, JAI Press, 1st edn, 1999.

[cit20] Tornøe C. W., Christensen C., Meldal M. (2002). J. Org. Chem..

[cit21] Goddard-Borger E. D., Stick R. V. (2007). Org. Lett..

[cit22] Boren B. C., Narayan S., Rasmussen L. K., Zhang L., Zhao H., Lin Z., Jia G., Fokin V. V., Zhang L., Chen X., Xue P., Sun H. H. Y., Williams I. D., Sharpless K. B., Fokin V. V., Jia G. (2008). J. Am. Chem. Soc..

[cit23] Chung B. K. W., Yudin A. K. (2015). Org. Biomol. Chem..

[cit24] Brust A., Wang C.-I. A., Daly N. L., Kennerly J., Sadeghi M., Christie M. J., Lewis R. J., Mobli M., Alewood P. F. (2013). Angew. Chem., Int. Ed..

[cit25] Wipf P., Fritch P. C., Geib S. J., Sefler A. M. (1998). J. Am. Chem. Soc..

[cit26] Packer M. S., Liu D. R. (2015). Nat. Rev. Genet..

