# Supplementary material for: Bypassing the proline/thiazoline requirement of the macrocyclase PatG
Source: Chem Commun (Camb). 2017 Nov 1;53(91):12274–7. doi: 10.1039/c7cc06550g (PMC5708355; doi:10.1039/c7cc06550g)
Supplement: Supplementary file 1 [file CC-053-C7CC06550G-s001.pdf]

This journal is © The Royal Society of Chemistry 2017

I. GENERAL INFORMATION AND MATERIALS 2

II. GENERAL PROCEDURES 4

PatGmac cloning, expression and purification 4

Solid-phase peptide synthesis of peptides 1-26 4

Pat Gmac macrocylization reaction of peptides 1-26 5

Synthesis of the non-natural amino acid building blocks 6

III. MS AND HPLC DATA OF STARTING AND FINAL HYBRID PEPTIDES 15

IV. NMR DATA OF FINAL CYCLIC PEPTIDES 17

V. NMR SPECTRA OF FINAL CYCLIC PEPTIDES 23

VI. MS-MS DATA OF ENZYMATIC REACTION PRODUCTS OF PEPTIDES 1-26 31

VII. MALDI-MS TRACES OF THE PATGMAC REACTIONS OF PEPTIDES 19 AND 20 37

VIII. LC-MS TRACES OF FINAL CYCLIC PEPTIDES 38

IX. LC-MS TRACES OF STARTING HYBRID PEPTIDES 1-26 47

X. REFERENCES 74

I. General information and materials

The HPLC grade acetonitrile (MeCN) was purchased from Fisher. Aqueous buffers and aqueous mobile-phases for HPLC were prepared using water purified with an Elga® Purelab® Milli-Q water purification system (purified to 18.2 MΩ.cm) and filtered over 0.45 μm filters. Solvents, amino acids and reagents were purchased commercially from different sources and used without any further purification. Anhydrous CH_3CN and MeOH were purchased from Sigma.

NMR spectra (^{1}H, 2D) of the cyclic peptides were recorded on a Bruker Ascend 700 spectrometer {δH (700 MHz), δC (175 MHz)} at ambient temperature in deuterated DMSO. NMR spectra ((^{1}H, 2D, ^{13}C) of the synthetic intermediates were recorded on a Bruker Ascend 500 spectrometer {δH (500 MHz), δC (126 MHz)} at ambient temperature in deuterated $CDCl_3$, unless otherwise stated. Chemical shifts are expressed in parts per million (ppm) from DMSO-d6 (δH = 2.50, δC = 39.52) or $CDCl_3$ (δH = 7.26, δC = 77.16).[1] Multiplicities are described as s (singlet), d (doublet), q (quadruplet), dd (doublet of doublets), dd (doublet of doublets of doublets), t (triplet), dt (doublet of triplets), m (multiplet), br (broad). Coupling constants *J* are quoted in Hertz (Hz) to the nearest 0.1 Hz. Signals of protons and carbons were assigned, as far as possible, by using the following two dimensional NMR spectroscopy techniques: [^{1}H, ^{1}H] COSY (Correlation Spectroscopy), [^{1}H, ^{1}H] TOCSY (Total Correlation Spectroscopy), [^{1}H, ^{1}H] ROESY (Rotating-frame NOE Spectroscopy), [^{1}H, ^{13}C] HSQC (Heteronuclear Single Quantum Coherence) and long range [^{1}H, ^{13}C] HMBC (Heteronuclear Multiple Bond Connectivity). EXSY (Exchange Spectroscopy) experiment was used to identify equilibrium chemical exchange at rt.

Low-resolution mass spectra were obtained with an Agilent 6130 single quad apparatus equipped with an electrospray ionization source. High-resolution mass spectra (HRMS) were obtained with a Thermo Exactive Orbitrap mass spectrometer or obtained from the EPSRC mass facility in Swansea.

Reactions performed in the enzymatic media were monitored using MALDI-MS acquired using a 4800 MALDI TOF/TOF Analyser (ABSciex, Foster City, CA) equipped with a Nd:YAG 355 nm laser and calibrated using a mixture of peptides. The spot was analysed in positive MS mode between 500 and 4000 m/z, by averaging

1000 laser spots. The samples, diluted in water to reduce the buffer concentration, (0.5 µL) were applied to the MALDI target along with alpha-cyano-4-hydroxycinnamic acid matrix (0.5 µL, 10 mg/mL in 50:50 acetonitrile:0.1% TFA) and allowed to dry. MSMS data were acquired using a TripleTOF 5600+. The sample was subjected to chromatography on an Acclaim PepMap 100 C18 trap and an Acclaim PepMap RSLC C18 column (ThermoFisher Scientific), using a nano-LC Ultra 2D plus loading pump and nano-LC as-2 autosampler (Eksigent). The sample was injected at neutral pH. The trap was washed with 2% acetonitrile, 0.05% trifluoroacetic acid, and the desired peptide was then eluted with a gradient of increasing acetonitrile, containing 0.1 % formic acid (15-40% acetonitrile in 5 min, 40-95% in a further 1 min, followed by 95% acetonitrile to clean the column, before re-equilibration to 15% acetonitrile). The eluent was sprayed into a TripleTOF 5600+ electrospray tandem mass spectrometer (Sciex) operating with standard nanospray conditions, and analysed in Product Ion Scan mode isolating the m/z of interest. The collision energy was adjusted to give optimal fragmentation. The MSMS fragmentation pattern was interrogated for diagnostic peaks.

Automated solid-phase peptide synthesis (SPPS) was carried out on a Biotage® Syro Wave™ system in polypropylene (PP) syringe with a PTFE frit. Final cleavage and deprotection were completed manually.

Analytical RP-HPLC was performed on an Agilent infinity 1260 series equipped with either a MWD detector and a single quadrupole MS using a Macherey-Nagel Nucleodur C18 column (10 µm x 4.6 x 250 mm) using the following chromatographic system. **System A:** 1 mL/min flow rate with MeCN and 0.1 % aqueous TFA [95% TFA (5 min), linear gradient from 5 to 95% of MeCN (35 min), 95% MeCN (40 min)] and UV detection at 220 nm.

Semi-preparative RP-HPLC was performed on an Agilent Infinity 1260 series equipped with an MWD detector using a Macherey-Nagel Nucleodur C18 column (10 µm x 16 x 250 mm at 10 mL/min or 10 µm x 21 x 250 mm at 21 mL/min) and fractions were collected automatically by peak detection at the specified wavelength using an Agilent 1260 Infinity preparative-scale fraction collector. Two chromatographic systems were used; **System P1**: MeCN and 0.1 % aqueous TFA [95% TFA (5 min), linear gradient from 5 to 95% of MeCN (35 min), 95% MeCN (40 min)] and UV detection at 280 nm.

II. General procedures

PatGmac cloning, expression and purification

The PatGmac enzyme was cloned from genomic DNA *(Prochlon sp.)* into the pHISTEV vector, expressed in *Escherichia coli* BL21 (DE3) cells grown on auto-induction medium, and purified as previously described by Koehnke *et al.*[2] However, subsequent to the Nickel column eluting with 250 mM imidazole, the remaining purification steps were replaced with dialysis in a bicine buffered solution [20 mM Bicine, 150 mM NaCl, pH 8.1] to remove the imidazole and the reducing agent.

Solid-phase peptide synthesis of peptides 1-26

The different precursor peptides were synthesized by standard automated solid-phase (SPPS) on a Chem-Matrix Rink amide resin (~ 0.5mmol/g) using the Fmoc strategy and Fmoc-protected amino acids (aa). A double coupling strategy using a 5-fold excess with HBTU/DIEA (HBTU, 0.5M in DMF and DIEA, 2M in NMP) and DIC/oxyma pure (DIC, 0.5M in DMF, Oxyma, 1M in DMF) was used for all amino acids for 30 minutes at 75 °C. The Fmoc deprotection was done in 20% piperidine/DMF for 12 min at rt.

The introduction of the triazole moiety was accomplished in two ways (Scheme S1):

1- Standard amide bond coupling of a pre-synthesized triazole containing dipeptide

2- Copper-catalyzed azide alkyne cycloaddition (CuAAC) on resin (1,4-triazole only)

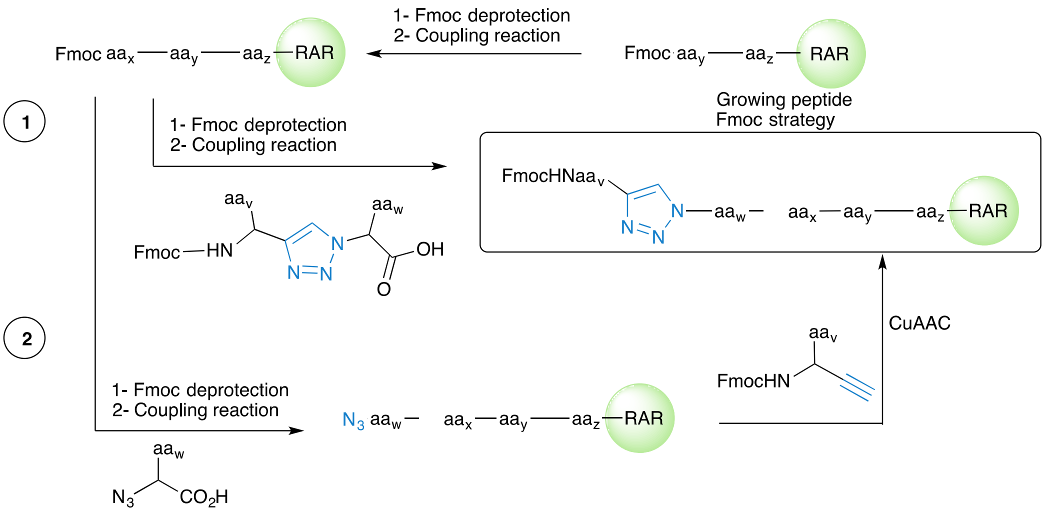

Scheme S1: Two SPPS strategies for the synthesis of triazole-containing peptides.

For the manual CuAAC reaction, the resin containing a terminal azide is washed with

5x THF and the corresponding amino alkyne (5 eq.) is diluted in THF and added to the resin. DIEA (10 eq.) is then added followed by CuI (5 eq.). The mixture is bubbled with dry nitrogen for 1 min then sealed, protected from light, and left overnight with shaking. After the reaction is complete (mini work up of a few beads; repeat the procedure if reaction not complete), the resin is washed with 4x THF, 4x H_2O, 4x THF, and 4x DMSO. The resin is swelled in DMSO for 10 min before the Fmoc deprotection step.

For the final cleavage and side chain deprotection, the beads (washed with CH_2Cl_2 and dried) were transferred into a flacon tube and the cleavage cocktail was added and left shaking for 2h: 96% TFA, 2.5% H_2O, 1.5% TIS. In the presence of cysteines, 1.5% of 1,2-ethanedithiol (EDT) was also added. The resin was filtered, washed with CH_2Cl_2, and the filtrate concentrated under reduced pressure. The peptides were then precipitated in cold Et_2O and the precipitate purified by HPLC. LCMS traces of peptides **1-26** can be found in section XI.
The disulfide bond of peptides **19-26** was formed overnight using 10% DMSO in TFA after full deprotection of the precursor peptides. The reaction was either done on the purified precursor, evaporated and freeze dried, or on the crude precursor, which was purified afterwards to afford the pure peptide.

Pat Gmac macrocylization reaction of peptides 1-26

The reactions were conducted in 20 mM bicine buffer, 500 mM NaCl, and 5% DMSO solution, pH 8.1 and incubated at 37 °C (without shaking) until full consumption of the starting peptide occured (MALDI monitoring). The reaction set-up was prepared in the following order; final concentrations:

1- A solution of the linear peptide in DMSO (between 10mM); 100µM
2- DMSO; 5%
3- Reaction buffer: 20mM Bicine, 150mM NaCl, pH 8.1
4- 5M NaCl; 500mM
5- PatGmac enzyme; 60µM

The small reaction set-ups were just aliquoted and analyzed by MALDI monitoring. The large-scale reaction mixtures were extracted 3 times with *n*-butanol (BuOH): H_2O (1/1, v/v). BuOH was added to the aqueous reaction, vigorously mixed, and then centrifuged for 10 min at high speed to help separate the two phases. The combined

BuOH fractions were evaporated under reduced pressure to dryness. The crude material was solubilized in a minimum volume of H_2O/MeCN and immediately purified by HPLC.

Synthesis of the non-natural amino acid building blocks

1- Experimental procedure for the synthesis of the Fmoc-protected (*R*)-β²-homoalanine

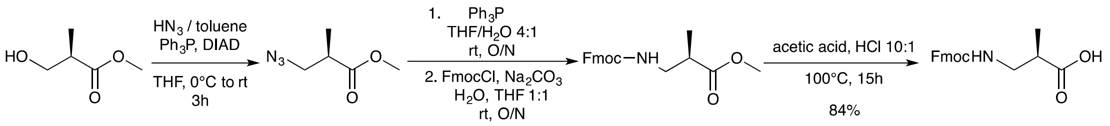

27 28

Scheme S2: Synthesis of the Fmoc-protected (*R*)-β²-homoalanine

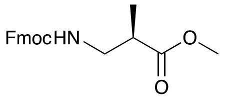

To a solution of triphenylphosphine (1.46g, 5.57mmol) and di-tert-butyl azodicarboxylate (1.28g, 5.57mmol) in anhydrous THF (15mL) at 0°C under inert atmosphere, a solution of hydrazoic acid* in toluene (1.1M, 5.3mL, 5.3mmol) was added drop-wise. Then a solution of the alcohol (280µL, 2.53mmol) solubilized in anhydrous THF (1.5mL) was added to the mixture and the reaction left stirring at room temperature for 3 hours. The reaction completion was monitored by TLC. The solvent was concentrated under reduced pressure and the crude mixture used for the next step without any purification.

The crude azide mixture (2.53mmol) and triphenylphosphine (0.73g, 2.78mmol) were solubilized in THF (24mL) and H_2O (6mL) (4/1, v/v) and the reaction stirred at room temperature overnight. The THF was evaporated under reduced pressure. Attempts to extract the amine into organic phase failed and the aqueous phase containing the amine was subsequently used for the next step.

To the amine (2.53mmol) in water (10mL) and THF (10mL) was added Na_2CO_3 (1g) and fluorenylmethyloxycarbonyl chloride (FmocCl, 1g, 3.85mmol) and the reaction was stirred at room temperature overnight. EtOAc (30mL) was added to the reaction and the two phases separated. The aqueous phase was extracted with EtOAc (2x30mL), and the combined organic phases washed with brine, dried over $MgSO_4$, filtered, and concentrated under reduced pressure. The crude mixture was purified

over silica gel (100% CH_2Cl_2 to 2% EtOAc/CH_2Cl_2) to afford the desired Fmoc-protected amine **27** (0.4g) as a white solid in 47% yield over three steps. The NMR spectroscopic data were in agreement with those described in the literature.[31] ^{1}H NMR (400 MHz, $CDCl_3$) δ=7.76 (d, *J* = 7.5, 2H), 7.58 (d, *J* = 7.4, 2H), 7.40 (tt, *J* = 7.3, *J* = 0.9, 2H), 7.31 (dd, *J* = 7.4, *J* = 0.9, 2H), 5.25-5.14 (m, 1H), 4.44-4.34 (m, 2H), 4.21 (t, *J* = 6.9, 1H), 3.72 (s, 3H), 3.45-3.26 (m, 2H), 2.78-2.66 (m, 1H), 1.19 (d, *J* = 7.3, 3H).

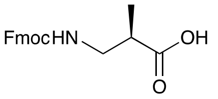

A solution of ester **27** (0.36g, 1.06mmol) in acetic acid (7.2mL) is treated with concentrated HCl (0.72mL) and the mixture heated at 100°C overnight. TLC monitoring showed the reaction completion. After cooling, the solution was poured into water (70mL). As no precipitation occurred as was expected, the solution was extracted with EtOAc (3x70mL) and the combined organic phases washed with brine, dried over $MgSO_4$, filtered, and concentrated under reduced pressure. The thick yellow oil obtained was recrystallized in pentane, and the precipitate triturated in pentane two times. Filtration afforded the desired carboxylic acid **28** (0.29g, 84%) as an off-white solid. The ^{1}H NMR showed two sets of peaks at room temperature. The major peaks were identical to the ones described in the literature. Further investigation showed that the two sets of peaks are for the same compound, possibly present in solution as two rotamers. **T=295K**: 7/3 ratio; ^{1}H NMR (400 MHz, $CDCl_3$, 295K) δ=7.76 (d, *J* = 7.4, 2H), 7.58 (d, *J* = 7.5, 2H), 7.40 (t, *J* = 7.3, 2H), 7.31 (t, *J* = 7.4, 2H), 6.29 (s, br, 0.3H), 5.26-5.18 (m, 0.7H), 4.60-4.47 (m, 0.6H), 4.47-4.32 (m, 1.4H), 4.30-4.17 (m, 1H), 3.49-3.39 (m, 0.7H), 3.38-3.28 (m, 0.7H), 3.16-3.00 (m, 0.6H), 2.84-2.68 (m, 0.7H), 2.41-2.29 (m, 0.3H), 1.24 (d, *J* = 7.2, 2.1H), 1.03 (d, *J* = 6.3, 0.9H). **T=253K**: 1/1 ratio; ^{1}H NMR (500 MHz, $CDCl_3$, 253K) δ=7.78 (t, *J* = 6.7, 2H), 7.59 (dd, *J* = 7.2, *J* = 2.7, 1H), 7.55 (d, *J* = 7.4, 1H), 7.45-7.38 (m, 2H), 7.37-7.28 (m, 2H), 7.02 (s, br, 1H), 5.34 (t, *J* = 7.4, 1H), 4.54 (dd, *J* = 10.5, *J* = 5.7, 1H), 4.46-4.31 (m, 3H), 4.25 (t, *J* = 5.9, 1H), 4.21 (t, *J* = 7.0, 1H), 3.49-3.37 (m, 1H), 3.34-3.25 (m, 1H), 3.14-3.04 (m, 1H), 3.03-2.94 (m, 1H), 2.81-2.71 (m, 1H), 2.31-2.21 (m, 1H), 1.23 (d, *J* = 7.2, 3H), 1.02 (d, *J* = 7.2, 3H).

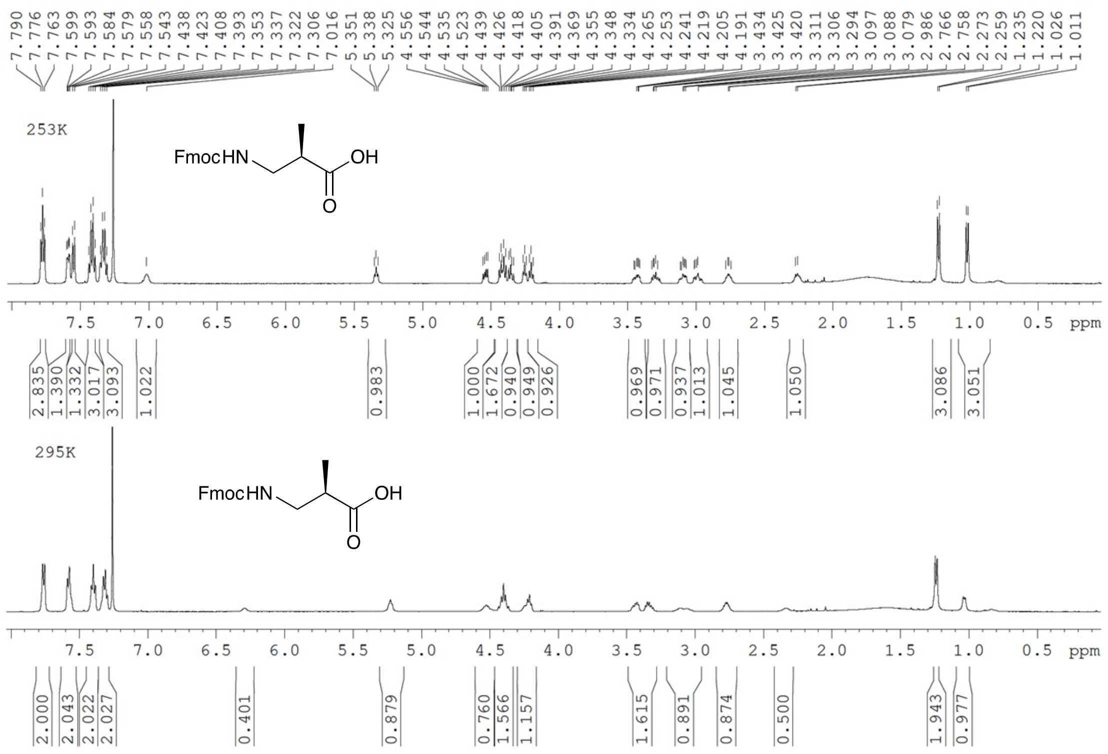

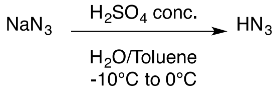

Scheme S3: Synthesis of hydrozoic acid HN_3.

Hydrazoic acid HN_3: To a paste mixture of sodium azide (6.5g, 0.1mmol) in water (6.5mL) at -10°C was added toluene (40 mL) under rigorous stirring. Concentrated sulphuric acid (2.66mL) was added drop-wise and the temperature monitored to keep under 10°C. The reaction temperature was cooled to 0°C and the solution decanted. The toluene fraction was dried over $MgSO_4$ and filtered. 1mL of the solution was diluted in 30mL of water and titrated with NaOH 1M to determine its concentration.

2- Experimental procedure for the synthesis of azido-acids and amino alkynes

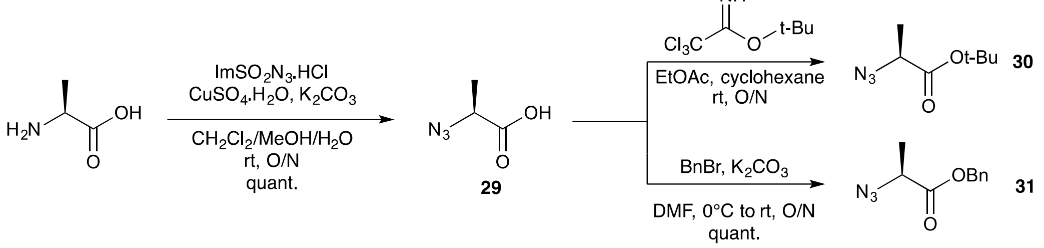

Scheme S4: General synthetic scheme of azido acid analogues

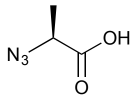

To a solution of *L*-Ala (1.4g, 15.7mmol) $CuSO_4.5H_2O$ (98.1mg, 0.39mmol) in a mixture of CH_2Cl_2 (44mL), MeOH (52mL), and H_2O (26mL) (1.7/2/1, v/v), $ImSO_2N_3.HCl$[4] (7.29g, 34.8mmol) was added and the pH adjusted to pH=9 with saturated aqueous K_2CO_3 solution. The mixture was stirred vigorously for 18h at room temperature. CH_2Cl_2 (52mL) was added to the reaction and separated. The organic phase was extracted with saturated aqueous $NaHCO_3$ solution (2x75mL). The combined aqueous phases were washed with Et_2O (2x75mL) and then the pH adjusted to pH = 2 with concentrated HCl (the aqueous solution changes colour from blue to colourless through shades of green and yellow). The acidic aqueous phase was then extracted with Et_2O (3x100mL). The combined organic layer (of the last three extractions) was dried over $MgSO_4$, filtered, and concentrated to afford azido acid **29** (1.8g, quant) as pale yellow oil. The NMR spectroscopic data were in agreement with those described in the literature. [5] The corresponding azido acids were then used without any further purification. ^{1}H NMR (400 MHz, $CDCl_3$) δ=4.03 (q, *J* = 7.2, 2H), 1.54 (d, *J* = 6.8, *3*H).

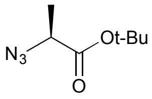

Cyclohexane (1.8mL) and tert-butyl-2,2,2-trichloroacetimidate (380mg, 1.74mmol) were added to a stirred solution of azido acid x (100mg, 0.87mmol) in ethyl acetate (4.5mL). The reaction mixture was stirred vigorously at room temperature overnight. The reaction mixture was washed with saturated aqueous $NaHCO_3$ solution (1x5mL) and H_2O (1x5mL). The organic layer was dried over $MgSO_4$, filtered, and concentrated. The crude solid obtained was triturated with pentane multiple times and the solid filtered away. The pentane filtrate was evaporated to afford the *tert*-butyl protected azido acid **30** (70mg, 47%) as light yellow oil. The NMR spectroscopic data were in agreement with those described in the literature.[6] ^{1}H NMR (500 MHz, $CDCl_3$) δ=3.79 (q, *J* = 7.2, 2H), 1.50 (s, 9H), 1.43 (d, *J* = 7.0, *3*H).

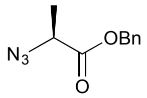

To a solution of azido acid **31** (1.8g, 15.7mmol) and K_2CO_3 (4.56g, 33mmol) in DMF (60mL) at 0°C under inert atmosphere, benzyl bromide (1.96mL,

16.5mmol) was added dropwise and the reaction left at room temperature overnight. Water (60mL) was added and the reaction extracted with EtOAc (3x100mL). The combined organic layer was washed with water and brine, dried over $MgSO_4$, filtered, and concentrated. Purification of the crude over silica gel column (30% pentane/CH_2Cl_2) afforded the desired benzyl ester **x** (2.63g, 82%) as colourless oil. The NMR spectroscopic data were in agreement with those described in the literature.[6] ^1H NMR (500 MHz, $CDCl_3$) δ=7.39-7.34 (m, 5H), 5.22 (s, 2H), 3.98 (q, J = 7.1, 2H), 1.49 (d, J = 7.2, 3H).

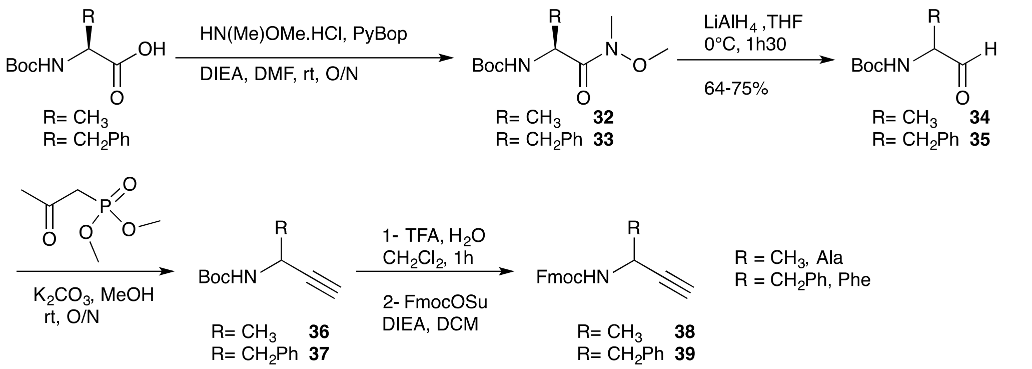

Scheme S5: General synthetic scheme of amino alkyne analogues

The azido-alkyne analogues were synthesized as previously described[7] following scheme S4. Epimerization is observed on the final product and is believed to have occurred during the reduction stage with $LiAlH_4$ on the aldehyde derivative.

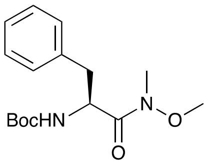

To a solution of Boc-Phe (3g,11.4mmol) and DIEA (2.98mL, 7.1mmol) in DMF (23mL) was added PyBop (6.5g, 12.5mmol) and the reaction was stirred at room temperature for 0.5 h. Weinreb amine (1,22g, 12.5mmol), DIEA (2.18mL, 12.5mmol) and DMF (15.3mL) were then added to the reaction mixture and left stirring at room temperature overnight. The reaction mixture was diluted with H_2O (50mL) and extracted with EtOAc (3x50mL). The combined organic fractions are washed successively with saturated aqueous $NaHCO_3$, $KHSO_4$ 1M, and brine. The organic fraction is dried over $MgSO_4$, filtered, and evaporated under reduced pressure. Purification over silica gel (100% CH_2Cl_2 to 10% EtOAC/CH_2Cl_2) afforded

the corresponding weinreb amide **33** (3.05g, 87%) as a light yellow oil.

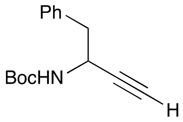

To a solution of the Weinreb amides **33** (2.87g, 9.4mmol) in anhydrous THF (30mL) at 0 °C, $LiAlH_4$ (0.44g, 11.2mmol) was added slowly and the reaction was stirred at 0 °C for 1.5 h. The reaction mixture was quenched at 0 °C by the addition of a solution of HCl 0.1M, untill bubbling stopped, and extracted with EtOAc (3x50mL). The combined organic fractions were washed with brine, dried over $MgSO_4$, filtered, and evaporated under reduced pressure. Aldehyde **35** obtained as a white solid was used for the net step without any further purification.
To a solution of the aldehyde **35** (7.5mmol) and K_2CO_3 (2.07g, 15mmol) in anhydrous MeOH (100mL), the Bestmann-Ohira reagent (dimethyl (1-diazo-2-oxopropyl)phosphonate; 1.35mL, 9mmol) was added and the reaction stirred at room temperature overnight. The reaction mixture was diluted with Et_2O and washed successively with 5% aqueous $NaHCO_3$ and H_2O, dried over $MgSO_4$, filtered, and evaporated under reduced pressure. Purification over silica gel (15% pentane/CH_2Cl_2) afforded the corresponding alkyne **37** (1.22g) as a white solid with 53% overall yield.

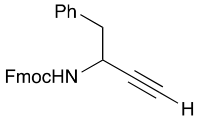

Alkyne **37** (1.22g, 4.97mmol) was diluted in CH_2Cl_2 (10mL) and TFA (7mL) and H_2O (0.5mL) were added and the reaction stirred at room temperature until full deprotection of the compound. The solvent was evaporated and resuspended in toluene and evaporated 3 times until dryness. The oily crude was diluted in CH_2Cl_2 (29mL) and DIEA (1.04mL, 5.96mmol) and FmocOSu (3.35g, 9,94mmol) were added and the reaction stirred at room temperature overnight. The mixture was washed successively with 3x HCl 1M, 3x $NaHCO_3$, and brine. The organic layer was dried over $MgSO_4$, filtered, and evaporated under reduced pressure. Purification over silica gel (20% pentane/CH_2Cl_2) afforded the Fmoc-protected amino-alkyne **39** (1.6g, 88%) as an off-white solid.

3- General procedure for the synthesis of 1,4 and 1,5-disubstituted 1,2,3-triazoles

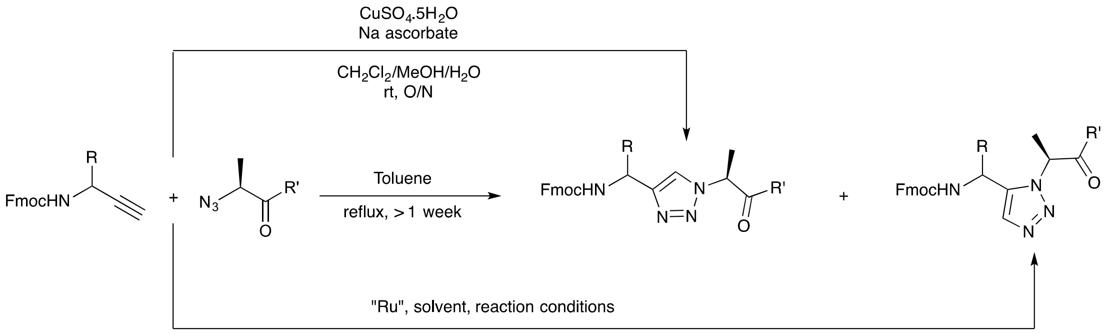

Scheme S6: General procedure for the synthesis of 1,4 and 1,5-disubstituted 1,2,3-triazoles

Procedure A: Thermal Huisgen reaction

A solution of the azide (1 eq.) and the alkyne (1 eq.) in toluene was heated and left stirring under reflux (110°C) untill full conversion was detected by NMR. The reaction was then cooled down to room temperature and toluene evaporated under reduced pressure. The crude was then purified over silica gel.

Procedure B: Copper catalysed azide alkyne cycloaddition (CuAAC) for the generation of 1,4-disubstituted 1,2,3-triazoles or *anti*-triazoles.

To a solution of the azide (1 eq.) and the alkyne (1 eq.) in $CH_2Cl_2/H_2O/MeOH$ (1/1/1, v/v/v) was added copper sulfate pentahydrate (0.1 eq.) and sodium ascorbate (0.2 eq.) and the reaction left stirring at room temperature overnight. The reaction mixture was diluted with H_2O and the reaction extracted with CH_2Cl_2 (3x). The combined organic fractions were washed with brine, dried over $MgSO_4$, filtered, and evaporated under reduced pressure.

Procedure C: Ruthenium catalysed azide alkyne cycloaddition (CuAAC) for the generation of 1,5-disubstituted 1,2,3-triazoles or *syn*-triazoles.

To a solution of the azide (1 eq.) and the alkyne (1 eq.) in anhydrous toluene under inert atmosphere was added RuCp*(cod)Cl (0.1 eq.) and the reaction left stirring at room temperature overnight. The reaction mixture was concentrated.

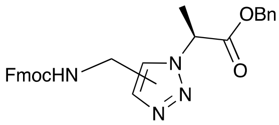

A mixture of *Anti*- and *Syn*-Fmoc-Gly-Tz-Ala-OBn was synthesized following procedure A. The two regioisomers **40** and **41** were inseperable by silica gel chromatography and RP-HPLC.

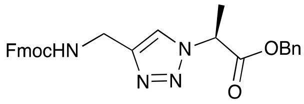

Anti-Fmoc-Gly-Tz-Ala-OBn was synthesized following procedure B. Only 30% conversion was achieved in these conditions. Triazole **40** was used for NMR reference only.

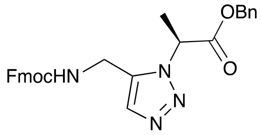

Syn-Fmoc-Gly-Tz-Ala-OBn was synthesized following procedure C. Triazole **41** was used for NMR reference only.

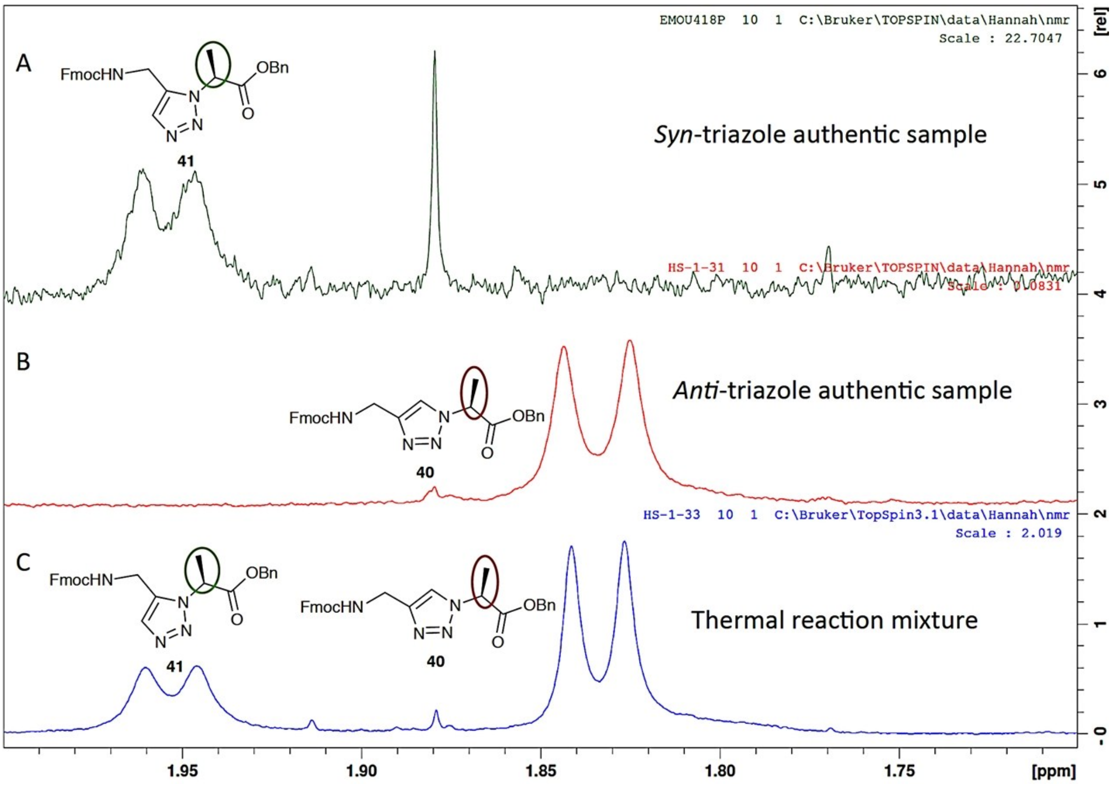

Figure S1: NMR comparison of the highlighted CH proton (Cα of Ala) in A) the authentic *syn*-triazole compound **41** obtained by ruthenium catalysis, B) the authentic *anti*-triazole compound **40** obtained by copper(I) catalysis, and C) the thermal

Huisgen reaction mixture containing both regioisomers at a 1:2 ratio of *syn* to *anti.*

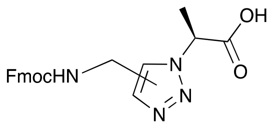

To a solution of the dipeptide mixture **40** and **41** (1.2 g, 2.49 mmol) in anhydrous methanol (6 mL) was added slowly 5% Pd/C (0.5 g, 0.5 mmol). The reaction flask was purged three times by three cycles of vacuum/inert atmosphere and left under inert atmosphere. Triethylsilane (3.98 mL, 24.9 mmol) was added dropwise (hydrogen gas generation *in situ*, vigorous bubbling of the solution) and the reaction left at room temperature. TLC monitoring showed the reaction was complete in under 10 minutes. The solution was then filtered over celite, washed with methanol, and evaporated under reduced pressure. The corresponding carboxylic acid regioisomeric mixture **42** and **43** was obtained as white solid in quantitative yiled. The regioisomeric mixture was inseparable by RP-HPLC and was used as a mixture for the synthesis of peptides **2**/**4** and **3**/**5**, which were separated by HPLC at the peptide stage.

4- Disulfide bond reduction of cyclic peptide 18c

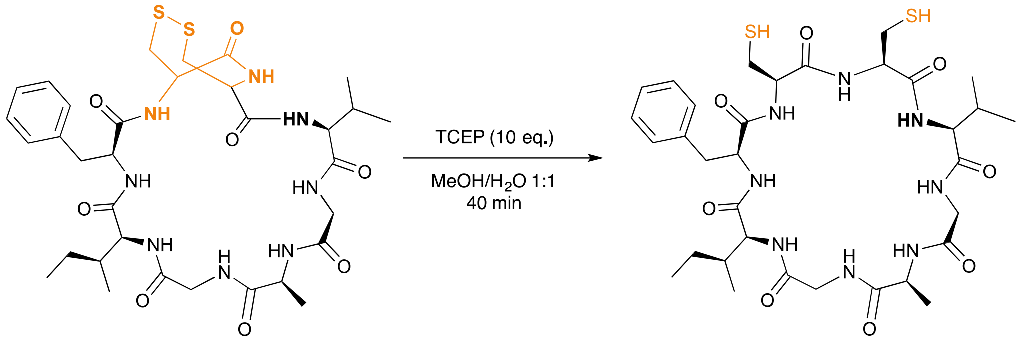

19c **19d**

Scheme S6: TCEP reduction of disulphide bond

Cyclic peptide **19c** was solubilized in methanol. 10 eq. of TCEP solubilized in the same amount of water (as methanol) were added to the peptide and left stirring at room temperature. The reaction was monitored by LCMS and found complete after 40 minutes (Figure S2). Peptide **19d** was purified by HPLC and the CD spectra in methanol of both cyclic peptides were recorded (Figure S3).

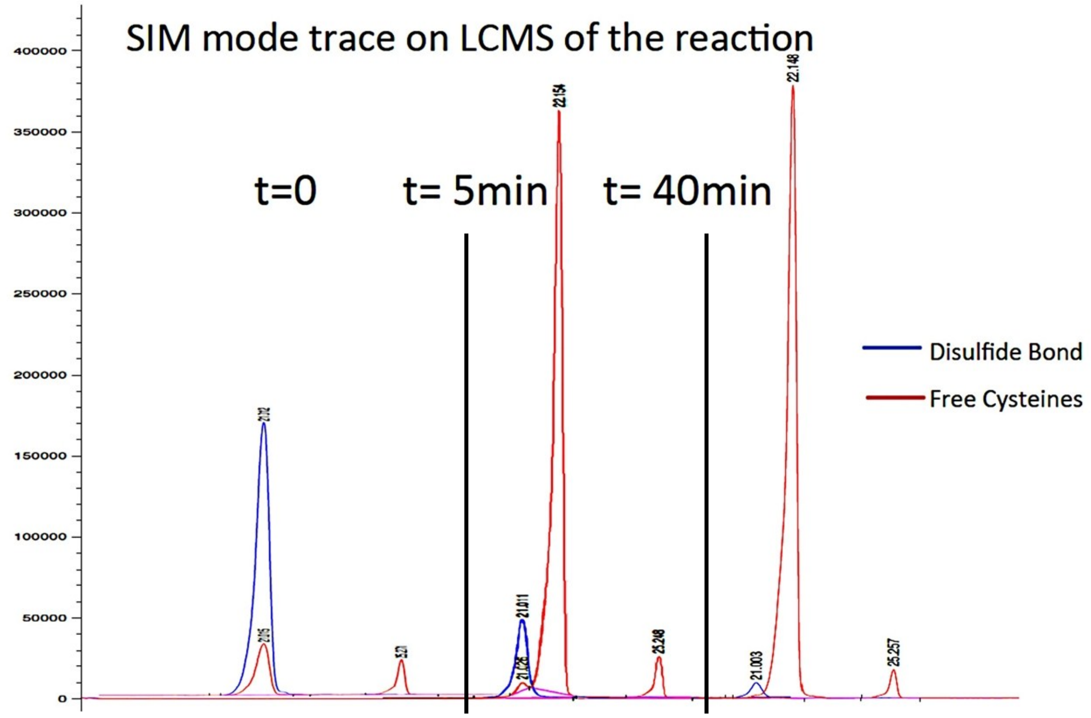

Figure S2: Disulfide bond reduction LCMS traces at different reaction times. Starting peptide **19c** $[M+H]^+$ MS trace is represented in Blue (Rt=21.0) and product **19d** $[M+H]^+$ MS trace is represented in red (Rt=22.1).

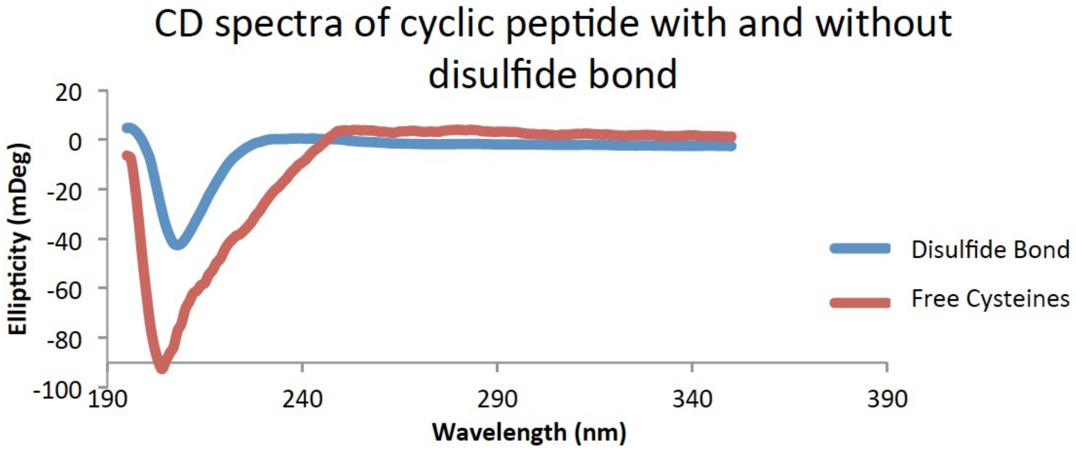

Figure S3: CD spectra of cyclic peptides **19c** (Blue) and **19d** (Red) in MeOH at rt.

III. MS and HPLC data of starting and final hybrid peptides

Table S1: MS data, HRMS, retention time, HPLC purities, and product information (under PatGmac reaction conditions) of starting hybrid peptides 7-24

	Peptide[a]	MS [M+H]$^+$	Calc. HRMS[b]	Found HRMS[b]	Rt[c]	Purity[d]	Pdct[e]	MS of product
**1**	VAGIGF**GTz$_{1\text{-}4}$A**AYD	1063.6	1063.5320	1062.5316	16.00	98%	NR	-
**2**	VGAGAI**GTz$_{1\text{-}4}$A**AYD	987.5	987.5007	987.5005	14.81	80%	NR	-
**3**	VGAGIGF**GTz$_{1\text{-}4}$A**AYD	1120.6	1120.5534	1120.5510	16.80	99%	NR	-
**4**	VGAGAI**G*Tz*$_{1\text{-}5}$A**AYD	987.5	987.5007	987.4986	15.05	76%	NR	-
**5**	VGAGIGF**G*Tz*$_{1\text{-}5}$A**AYD	1120.6	1120.5534	1120.5501	16.96	99%	NR	-
**6**	VGAGAI**GP**AYD	989.5	989.5051	989.5048	14.78	97%	NR	-
**7**	VGAGIG**AP**AYD	989.5	989.5051	989.5049	14.81	99%	C	623.3
**8**	VGAGIG**FP**AYD	1065.4	1065.5364	1065.5372	16.97	99%	C	699.4
**9**	VGAGIG-**$_{\beta}$A-P**AYD	989.5	989.5051	989.5055	13.79	99%	NR	-
**10**	VGAGIG-**$_{\beta^2\text{-homo}}$A-P**AYD	1003.5	1003.5207	1003.5212	14.19	97%	NR	-
**11a**	VGAGIG**FTz$_{1\text{-}4}$A**AYD	1063.5	1063.5320	1063.5318	17.25	99%	C	697.3
**11b**	VGAGIG***f*Tz$_{1\text{-}4}$A**AYD	1063.5	1063.5320	1063.5312	17.46	99%	NR	-
**12**	VGAGIG**fP**AYD	1065.4	1065.5364	1065.5339	17.14	97%	NR	-
**13a**	VGAGIG**FTz$_{1\text{-}4}$G**AYD	1049.5	1049.5163	1049.5150	15.91	99%	NR	-
**13b**	VGAGIG***F*Tz$_{1\text{-}4}$G**AYD	1049.5	1049.5163	1049.5153	16.16	99%	NR	-
**14**	VGAGIG**ATz$_{1\text{-}4}$A**AYD	987.5	*985.4861*	*985.4870*	14.18	95%	C	621.3
**15**	ITA**Tz$_{1\text{-}4}$A**IT**FTz$_{1\text{-}4}$A**AYD	1203.6	1203.6269	1203.6285	17.43	95%	C	-
**16**	V-PEG$_4$-**FTz$_{1\text{-}4}$A**AYD	955.5	955.4884	955.4889	16.54	98%	C	589.3
**17**	PEG$_4$-**FTz$_{1\text{-}4}$A**AYD	856.4	856.4199	856.4201	15.98	99%	C	490.3
**18**	PEG$_4$-**ATz$_{1\text{-}4}$A**AYD	781.4	781.3727	781.3709	13.52	99%	C	414.2
**19**	VGAGIF[CC]AYD	1115.5	1115.4649	1115.4643	17.40	98%	C	749.4
							L	767.4
**20**	VPAPIP[CC]AYD	1145.5	1145.5118	1145.5114	15.94	99%	L	797.4
**21**	VGAGIF[cc]AYD	1115.5	1115.4649	1115.4646	17.69	96%	NR	-
**22**	FyKT[CC]AYD	1110.3	*1108.4238*	*1108.4210*	14.27	95%	L	762.3
**23**	LKYG[CC]AYD	1032.2	*1030.4132*	*1030.4098*	13.59	98%	NR	-
**24**	LGKYG[CC]AYD	1089.5	1089.4492	1089.4497	13.98	99%	C	723.4
**25**	GYKLG[CC]AYD	1089.5	*1087.4347*	*1087.4311*	13.38	99%	C	723.4
							L	741.4
**26**	LGYKLG[CC]AYD	1202.5	*1200.5187*	*1200.5149*	14.75	99%	C	836.4
							L	854.4

[a] Tz$_{1\text{-}4}$= 1,4-*anti*-triazole, Tz$_{1\text{-}5}$= 1,5-*syn*-triazole, βA= β-Alanine, β^2-homoA= (R)-3-amino-2-methylpropanoic acid, *D*-amino acids in lower case, [CC]= disulphide bond.
[b] Calculated and found HRMS values are for [M+H]$^+$ unless written in *italics*, indicating it is [M-H]$^-$
[c] Retention time as observed by analytical HPLC following system A.
[d] Purity assessed by analytical HPLC at 220 nm UV absorption. LCMS traces of all precursor peptides can be found in section XV.
[e] The product obtained after reaction of the starting peptide with PatGmac as detected by Maldi. C: Cyclic peptide, L: Linear peptide, NR: no reaction.

Table S2: MS data, HRMS, retention time, HPLC purities, and yields of the final cyclic hybrid peptides

	Cyclic Peptide	MS [M+H]$^+$	Calcd HRMS [M+H]$^+$	Found HRMS [M+H]$^+$	Calcd HRMS [M+Na]$^+$	Found HRMS [M+Na]$^+$	rt	Purity (%)[a]	Yield (%)[b]
**11c**	VGAGIGF**TzA**	697.4	697.3780	697.3777	719.3600	719.3594	18.58	95%	40%
**11d**	VGAGIGF**TzA**	697.4		697.3776		719.3595	18.88	73% 68/32[c]	
**14c**	VGAGIGA**TzA**	621.3	621.3467	-	643.3287	643.3282			40%
**16c**	V-PEG$_4$-**FTzA**	589.3	589.3344	589.3342	611.3164	611.3153	19.88	99%	36%
**16d**	V-PEG$_4$-**FTzA**	589.3	589.3344	589.3343	611.3164	611.3155	20.24	100% 70/30[d]	
**17c**	PEG$_4$-**FTzA**	490.3	490.2660	490.2663	512.2480	512.2478	18.84	98%	34%
**19c**	VGAGIF[CC]	749.3	749.3109	749.3110	771.2929	771.2930	21.02	95%	37%
**19d**	VGAGIFCC	751.3	751.3266	751.3266	773.3085	773.3086	22.10	95%	-

[a] Purity assessed by analytical HPLC at 220 nm UV absorption. LCMS traces can be found in section XIII
[b]The yield of the enzymatic transformation(s) leading to the compound.
[c] The purity indicated relates to the cyclic peptide **11c** + **11d**; 68/32 indicates the ratio **11d**/**11c**
[d] The purity indicated relates to the cyclic peptide **16c** + **16d**; 70/30 indicates the ratio **16d**/**16c**

IV. NMR data of final cyclic peptides

Copies of the proton NMR spectra for each compound depicting the different species, when present, by color code as well as copies of the HSQC spectra and EXSY spectra can be found in sections VI and VII.

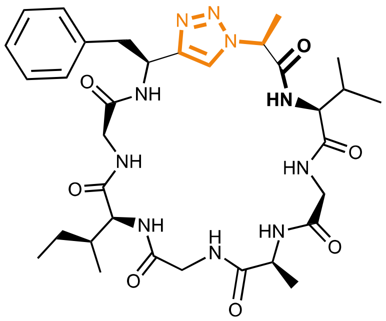

11c;11d; Cyclo(-VG$_a$AG$_b$IG$_c$FTz$_{1\text{-}4}$A-)

^{1}H NMR: (700 MHz, DMSO); HSQC and HMBC analysis (700 MHz, DMSO)

Amino acid*	Atom	^{1}H CS	^{13}C CS	^{1}H CS	^{13}C CS
Val	NH	8.68, d, *J* = 8.7	-	8.65, d, *J* = 5.9	-
	α CH	4.11, t, *J* = 7.7	58.5	3.82, t, *J* = 6.7	60.2
	β CH	1.99-1.93, m	27.0	1.95-1.90, m	29.6
	γ CH_3	0.90, d, *J* = 6.7;	18.8	0.92, d, *J* = 6.7;	19.4
		0.86, d, *J* = 6.7	19.5	0.86, d, *J* = 6.5	19.4
	CO	-	ND		171.6

Gly (a)	NH	8.35-8.33, m	-	8.43-8.40, m	-
	α CH_2	4.09-4.03, m; 3.41, dd, J = 3.6, J = 17.2	42.1	4.04-4.00, m; 3.40, dd, J = 3.9, J = 16.9	42.3
	CO	-	ND	-	168.5
Ala	NH	7.89, d, J = 8.4	-	7.75-7.71, m	-
	α CH	4.32-4.27, m	47.9	4.34, t, J = 7.4	48.3
	β CH_3	0.93, d, J = 7.2	18.1	1.26, d, J = 7.2	18.4
	CO	-	172.5	-	172.9
Gly (b)	NH	7.95, t, J = 5.7	-	8.20-8.16, m	-
	α CH_2	3.86-3.81, m; 3.64, dd, J = 5.0, J = 16.7	41.8	3.99-3.94, m; 3.54-3.49, m	41.9
	CO	-	ND	-	169.0
Ile	NH	8.00, d, J = 8.3	-	7.75-7.71, m	-
	α CH	4.09-4.03, m	57.5	4.16, t, J = 7.5	57.0
	β CH	1.74-1.67, m	35.5	1.69-1.62, m	37.3
	γ CH_2	1. 43-1.37, m; 1.09-1.03, m	24.6	1.48-1.38, m; 1.06-1.00, m	24.6
	γ CH_3	0.74, d, J = 6.8	15.5	0.77, d, J = 6.9	15.4
	δ CH_3	0.78, t, J = 7.4	11.2	0.80, t, J = 7.4	11.2
	CO	-	ND	-	171.3
Gly (c)	NH	8.24-8.21, m	-	8.31-8.28, m	-
	α CH_2	3.92, dd, J = 7.3, J = 16.7; 3.37-3.31, m	43.7	4.01-3.98, m; 3.24, dd, J = 4.5, J = 16.5	42.2
	CO	-	ND	-	168.3
Phe	NH	8.35-8.33, m	-	8.26, d, J = 9.2	-
	α CH	5.18-5.14, m	ND	5.19-5.14, m	ND
	β CH_2	3.02, dd, J = 5.2, J = 13.7; 2.95, dd, J = 9.8, J = 13.7	41.6	3.37, 3.31, m; 2.85, dd, J = 10.4, J = 13.8	40.0
	Ar CH	7.24-7.21, m<br>7.18-7.16, m	126.7/129.5<br>128.3	7.27-7.23, m<br>7.19-7.16, m	128.3/129.5<br>126.6
	Ar C		138.6		138.8
TzAla	Tz CH	8.01, s	121.5	8.06, s	121.7
	Tz C	-	148.5	-	149.0
	α CH	5.59, q, J = 7.1	57.9	4.54, q, J = 7.3	57.7
	β CH_3	1.59, d, J = 7.1	28.5	1.61, d, J = 7.1	18.7
	CO	-	169.4	-	169.7

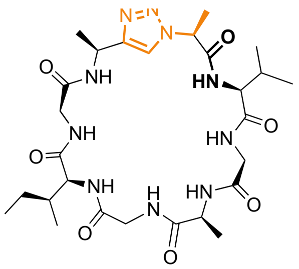

14c; Cyclo(-$VG_aA_aG_bIG_cA_bTz_{1\text{-}4}A$-)

^{1}H NMR: (700 MHz, DMSO); HSQC and HMBC analysis (700 MHz, DMSO)

**Amino acid***	**Atom**	**^{1}H CS**	**^{13}C CS**
Val	NH	8.72, d, *J* = 5.0	-
	α CH	33.79-3.76, m	60.8
	β CH	1.96-1.88, m	29.4
	γ CH_3	0.93, d, *J* = 6.7;	19.2
		0.85, d, *J* = 6.7	19.2
	CO		ND
Gly (a)	NH	8.49-8.43, m	-
	α CH_2	4.02-3.89, m;	42.4
		3.41-3.73, m	
	CO	-	ND
Ala (a)	NH	7.69, d, *J* = 7.7	-
	α CH	4.33-4.27, m	48.5
	β CH_3	1.26, d, *J* = 7.3	18.4
	CO	-	ND
Gly (b)	NH	8.16-8.10, m	-
	α CH_2	4.02-3.89, m;	42.2
		3.56-3.50	
	CO	-	173.1
Ile	NH	7.62, d, *J* = 7.9	-
	α CH	4.18, t, *J* = 7.5	56.8
	β CH	1.73-1.64, m	37.2
	γ CH_2	1.45-1.39, m; 1.08-0.99, m	24.6
	γ CH_3	0.82-0.77, m	15.5
	δ CH_3	0.82-0.77, m	11.4
	CO	-	ND
Gly (c)	NH	8.42-8.37, m	-
	α CH_2	4.02-3.89, m;	42.5
		3.49-3.44, m	
	CO	-	ND
Ala (b)	NH	8.16-8.10, m	-
	α CH	5.05 , t, *J* = 7.7	41.1
	β CH_3	1.38, d, *J* = 7.2	20.6
TzAla	Tz CH	7.92, s	120.9
	Tz C	-	150.0
	α CH	5.52, q, *J* = 7.3	57.8
	β CH_3	1.60, d, *J* = 7.5	18.5
	CO	-	169.9

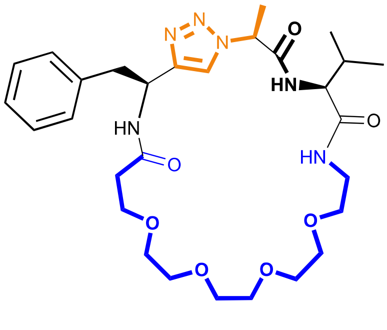

16c

16c;16d; Cyclo(-V(PEG)$_4$-FTz$_{1\text{-}4}$A-)

^{1}H NMR: (700 MHz, DMSO); HSQC and HMBC analysis (700 MHz, DMSO)

**Amino acid***	**Atom**	**^{1}H CS**	**^{13}C CS**	**^{1}H CS**	**^{13}C CS**
Phe	NH	8.34, d, *J* = 8.8	-	8.31, d, *J* = 8.7	-
	α CH	5.18, dt, *J* = 9.5, *J* = 4.1	47.3	5.20-5.15, m	47.3
	β CH_2	3.36, dd, *J* = 4,1, *J* = 13.9; 2.89, dd, *J* = 10.6, *J* = 13.8	40.1	3.20-3.15, m; 3.04-2.96, m	39.0
	Ar CH	7.28-7.21, m;<br>7.20-7.16	128.4/129.5<br>126.5	7.28-7.21, m;<br>7.20-7.16	128.4/129.5<br>126.5
	Ar C		138.7		ND
TzAla	Tz CH	7.80, s	121.2	7.90, s	121.5
	Tz C	-	149.3	-	147.9
	α CH	5.57, q, *J* = 7.1	58.1	5.60-5.52, m	58.1
	β CH_3	1.61, d, *J* = 7.2	19.1	1.59, d, *J* = 7.0	19.2
	CO	-	168.7	-	169.7
Val	NH	8.39, d, *J* = 8.7	-	8.59, d, *J* = 7.6	-
	α CH	4.07, dd, *J* = 7.4, *J* = 8.5	58.5	4.17-4.12, m	57.9
	β CH	1.96-1.89, m	30.5	1.98-1.93, m	30.8
	γ CH_3	0.81, d, *J* = 5.3;<br>0.80, d, *J* = 5.1	18.4<br>19.4	0.85, d, *J* = 5.2;<br>0.84, d, *J* = 5.5	18.4<br>19.4
	CO		170.9		168.7
PEG$_4$	NH	8.00-7.96, m	-	8.10-8.07, m	-
	CH_2(NH)	3.50-3.40, m;<br>3.03-2.98, m	39.2	3.04-2.96, m;<br>3.04-2.96, m	39.2
	CH_2(CO)	2.42-2.37, m;<br>2.09, dt, *J* = 4.7, *J* = 14.4	36.5	2.42-2.28, m;<br>2.18-2.13, m	36.3
	CH_2(CH_2 CO)	3.63, dt, *J* = 4.3, *J* = 9.4;<br>3.50-3.44, m	67.1	3.60-3.54, m;	66.7
	7x CH_2	3.54-3.37, m	72.0-68.3	3.62-3.33, m	66.6-71.0
	CO	-	170.3	-	ND

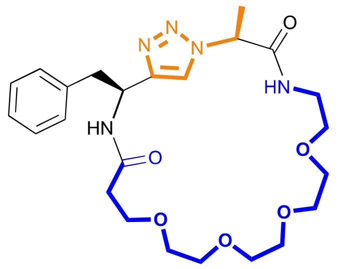

17c

17c; Cyclo(-V(PEG)$_4$-FTz$_{1\text{-}4}$A-)

^{1}H NMR: (700 MHz, DMSO); HSQC and HMBC analysis (700 MHz, DMSO)

**Amino acid***	**Atom**	**^{1}H CS**	**^{13}C CS**
Phe	NH	8.31, d, *J* = 8.9	-
	α CH	5.24-5.20, m	47.2
	β CH_2	2.92, dd, *J* = 9.7, *J* = 13.6;<br>3.26, dd, *J* = 5.1, *J* = 13.7	40.5
	Ar CH	7.26-7.22, m;<br>7.22-7.19, m<br>7.19-7.15	128.4<br>129.6<br>126.6
	Ar C		138.5
TzAla	Tz CH	7.83, s	121.3
	Tz C	-	149.0
	α CH	5.31, q, *J* = 7.0	58.5
	β CH_3	1.63, d, *J* = 7.1	18.7
	CO	-	168.7
PEG_4	NH	8.42-8.37, m	-
	CH_2(NH)	3.36-3.31, m;<br>3.20-3.14, m	39.3
	CH_2(CO)	2.43-2.34, m;<br>2.10-2.05, m	36.5
	CH_2(CH_2CO)	3.64, td, *J* = 3.4, *J* = 9.6;<br>3.52-3.44, m	67.0
	7x CH_2	3.51-3.40, m	67.7-72.3
	CO	-	170.2

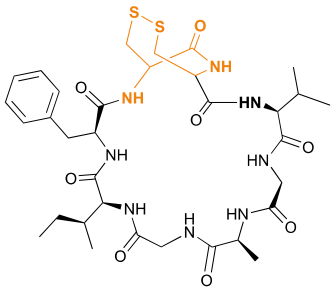

19c; Cyclo(-VGAGIF[CC]-)

^{1}H NMR: (700 MHz, DMSO); HSQC and HMBC analysis (700 MHz, DMSO)

Amino acid*	Atom	^{1}H CS	^{13}C CS
Val	NH	7.51, d, *J* = 9.2	-
	α CH	4.21, d, *J* = 7.9	58.8
	β CH	2.04-1.95, m	31.0
	γ CH_3	0.90-0.85, m;	18.7
		0.90-0.85, m	19.7
	CO	-	ND
Gly (a)	NH	8.04-8.00, m	-
	α CH_2	3.89-3.83, m;	41.6
		3.64-3.51, m	
	CO	-	ND
Ala	NH	8.21, d, *J* = 8.1	-
	α CH	4.49, t, *J* = 7.4	47.7
	β CH_3	1.21, d, *J* = 7.1	19.0
	CO	-	173.5
Gly (b)	NH	8.39-8.31, m	-
	α CH_2	4.12-4.06, m;	43.0
		3.55-3.47, m	
	CO	-	ND
Ile	NH	7.96-7.90, m	-
	α CH	3.99-3.95, m	58.7
	β CH	1.69-1.59, m	36.0
	γ CH_2	1.25-1.18, m;	23.5
		1.00-0.92, m	
	γ CH_3	0.59-0.55, m	15.5
	δ CH_3	0.59-0.55, m	11.8
	CO	-	ND
Phe	NH	7.71, d, *J* = 7.9	-
	α CH	4.58-4.50, m	53.8
	β CH_2	3.16-3.07, m; 2.88-2.80, m	36.7
	Ar CH	7.29-7.21, m;	129.6/128.4
		7.21-7.15, m	126.6
	Ar C		138.2
	CO	-	ND
Cys	NH	8.13-8.09, m	
	α CH	4.95-4.85, m	52.2 or 54.1
	β CH_2	3.16-3.07, m; 2.88-2.80, m	39.7
	CO	-	ND
Cys	NH	8.17, d, *J* = 11.6	
	α CH	4.95-4.85, m	52.2 or 54.1
	β CH_2	2.75-2.71, m	42.0
	CO	-	ND

V. NMR spectra of final cyclic peptides

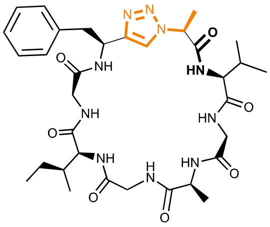

11c; Cyclo(-VGaAG$_b$IG$_c$FTz$_{1\text{-}4}$A-); DMSO-d6; 700 MHz

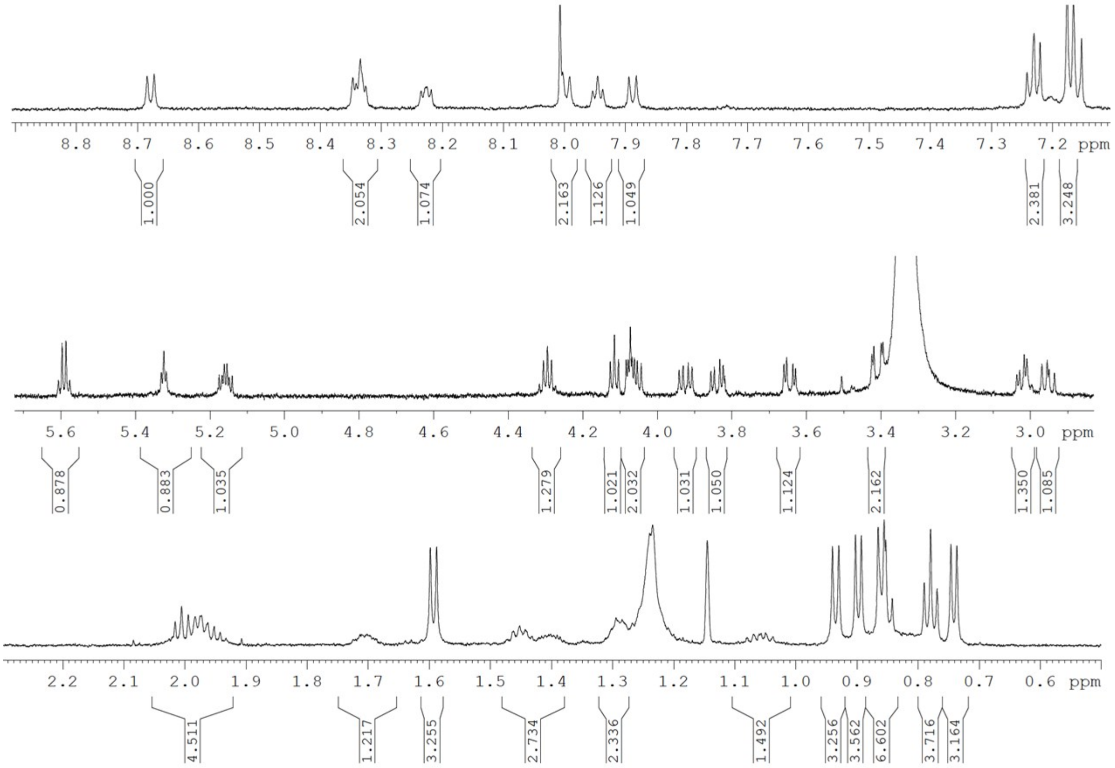

11c; Cyclo(-VGaAG$_b$IG$_c$FTz$_{1\text{-}4}$A-); HSQC (green/blue) and HMBC (red) spectra

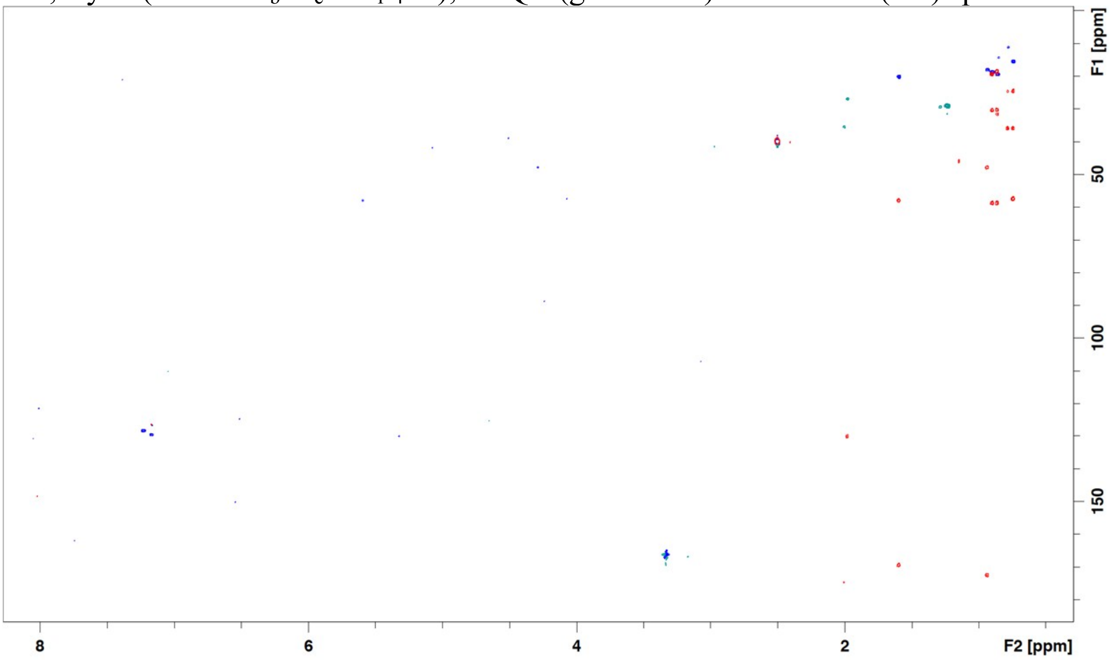

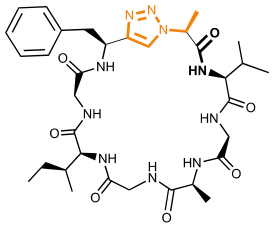

11c, 11d; Cyclo(-VGaAG$_b$IG$_c$FTz$_{1\text{-}4}$A-); DMSO-d6; 700 MHz

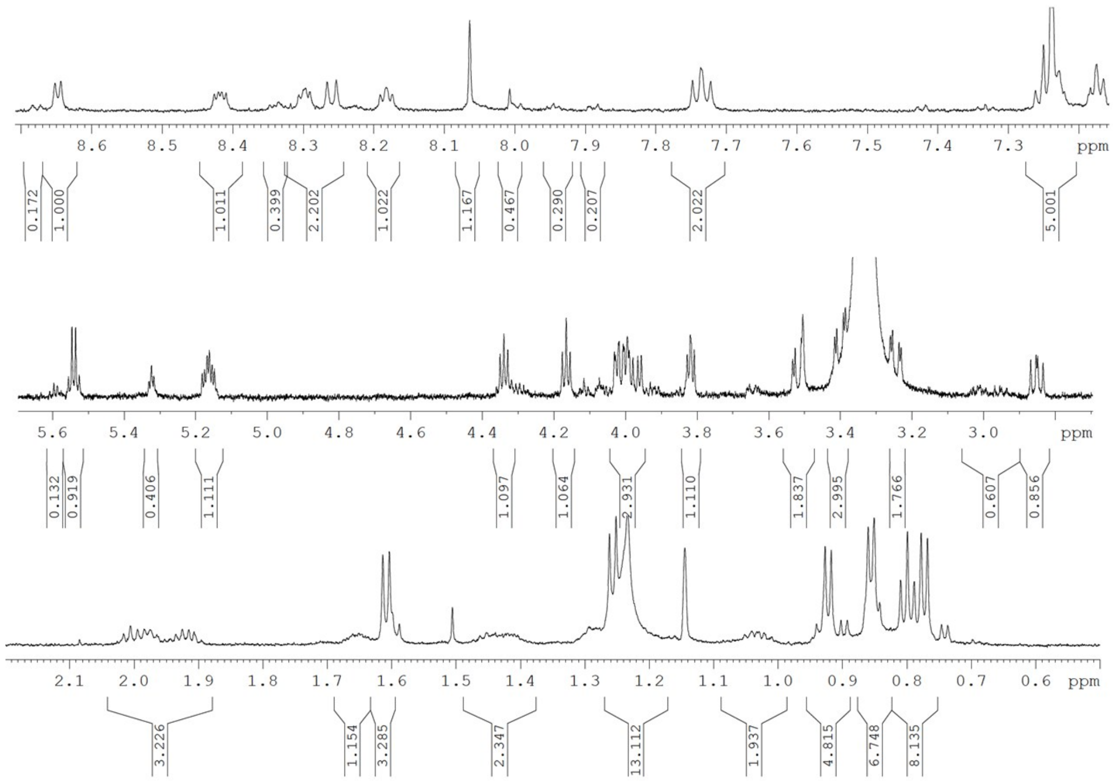

11c, 11d; Cyclo(-VGaAG$_b$IG$_c$FTz$_{1\text{-}4}$A-); HSQC (green/blue) and HMBC (red) spectra

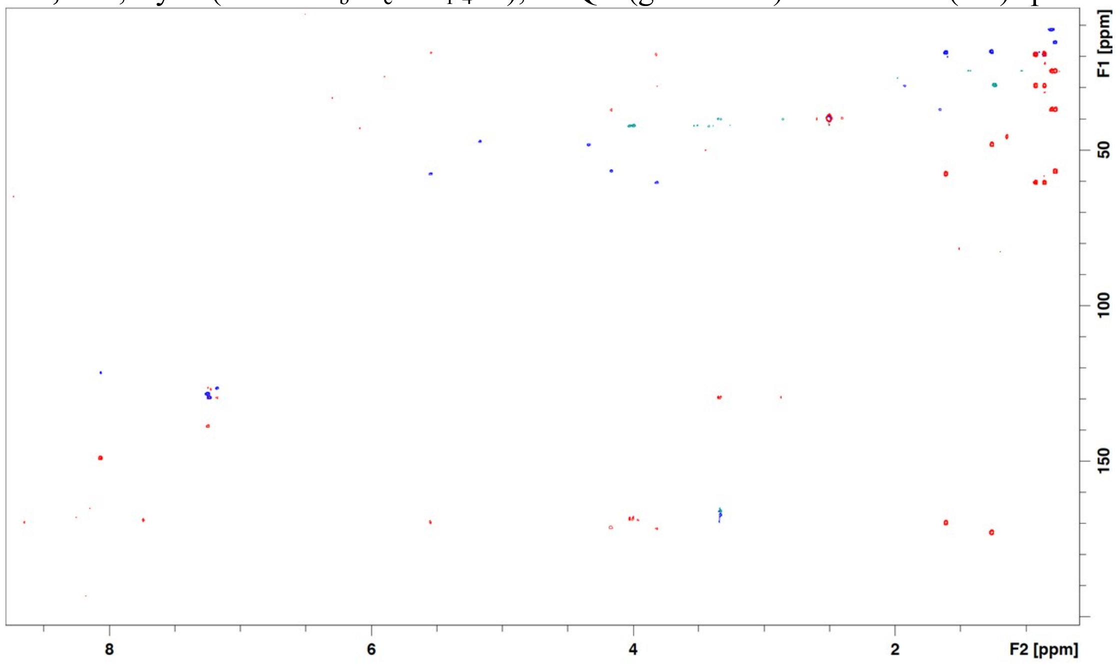

11c, 11d; Cyclo(-VGaAG$_b$IG$_c$FTz$_{1\text{-}4}$A-); EXSY

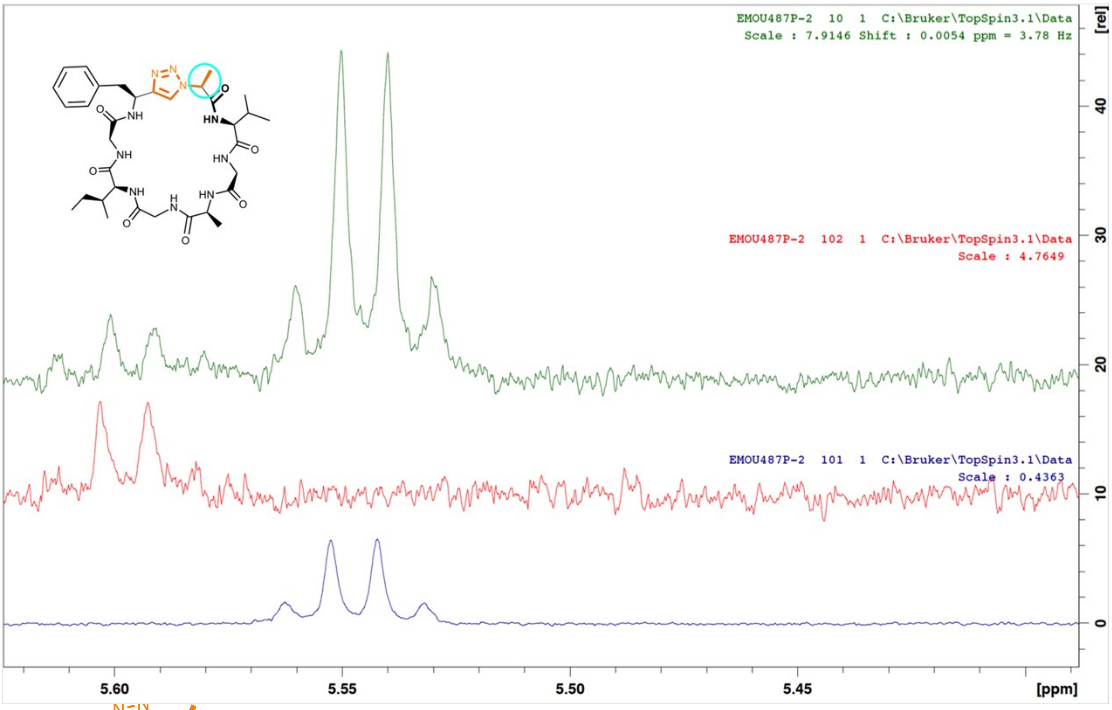

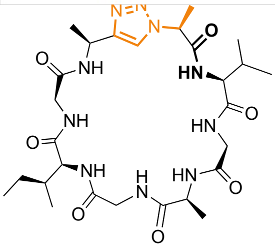

14c; Cyclo(-VG$_a$A$_a$G$_b$IG$_c$A$_b$Tz$_{1\text{-}4}$A-); DMSO-d6; 700 MHz

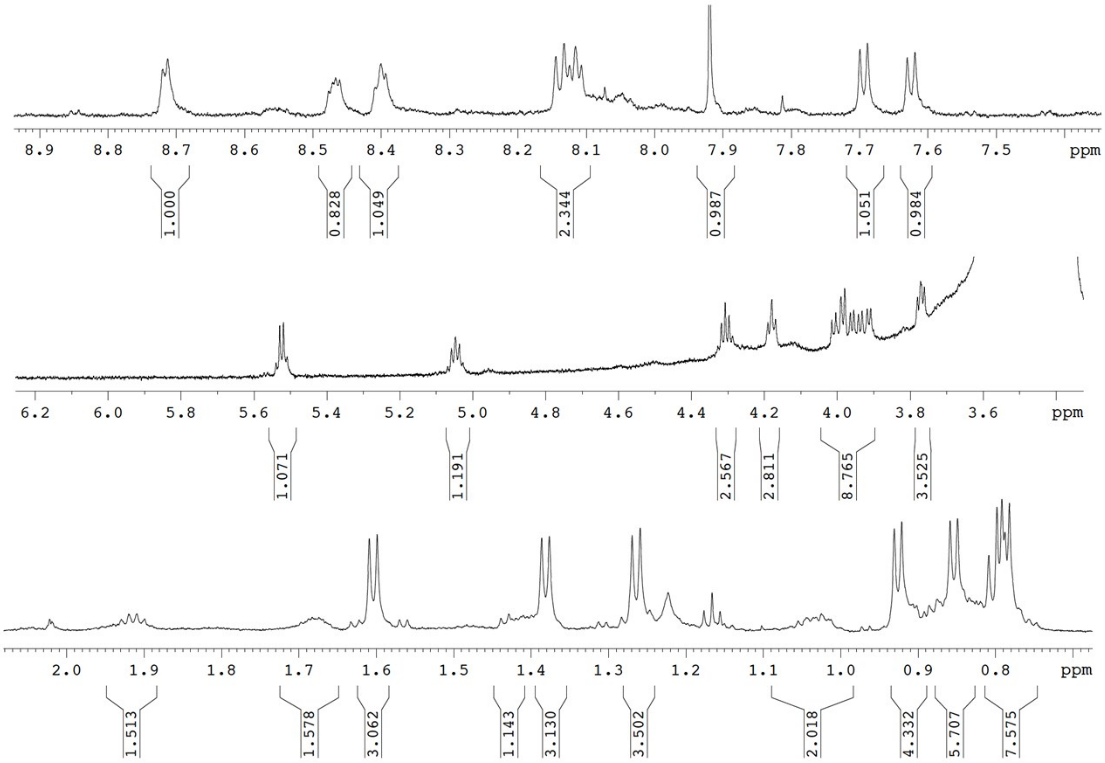

14c; Cyclo(-V$G_a$$A_a$$G_bIG_c$$A_b$$Tz_{1\text{-}4}$A-); HSQC (green/blue) and HMBC (red) spectra

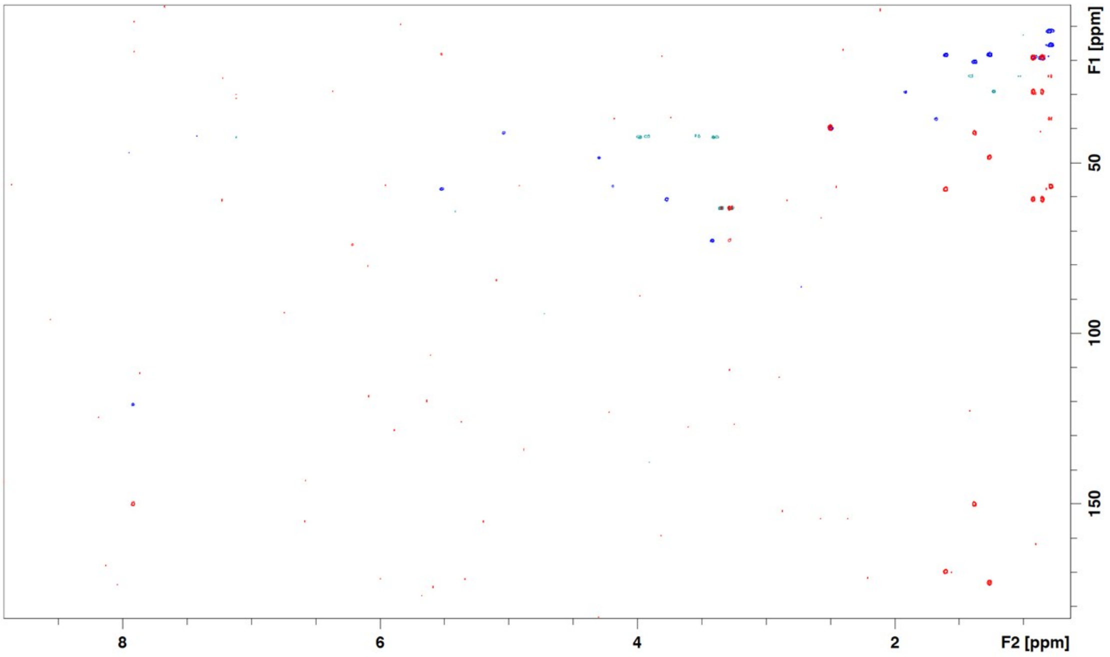

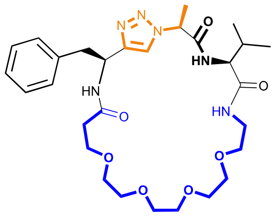

16c; Cyclo(-V$(PEG)_4$F$Tz_{1\text{-}4}$A-); DMSO-d6; 700 MHz

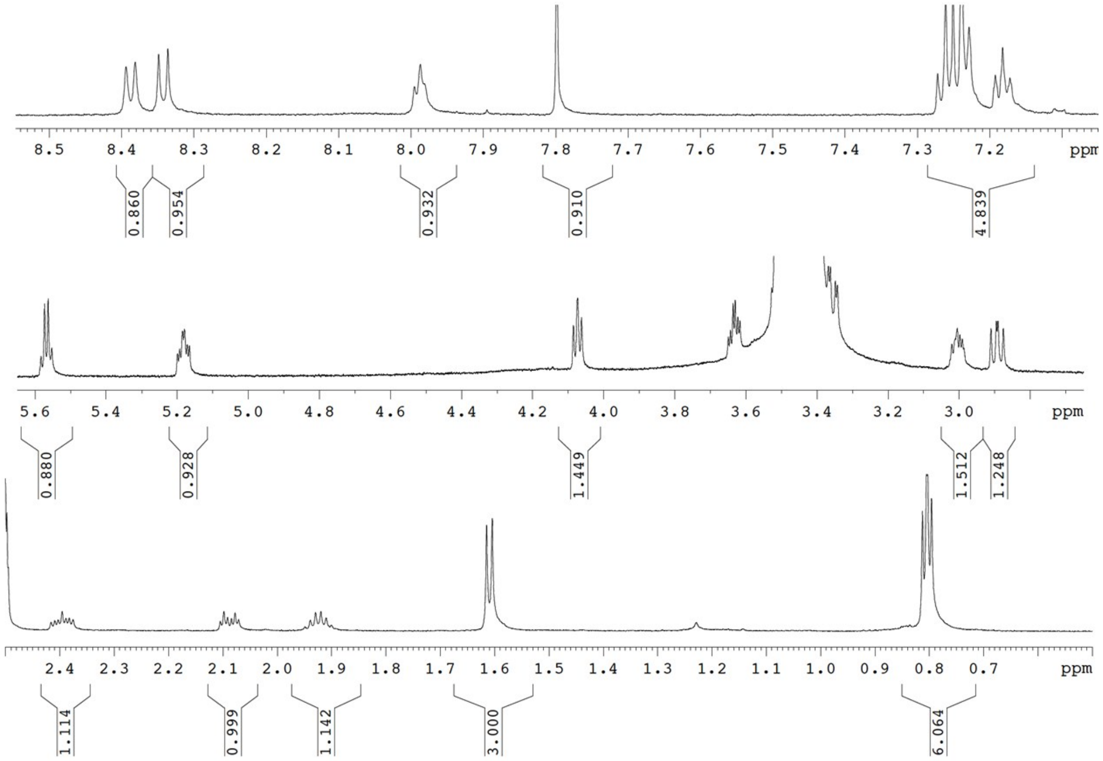

16c; Cyclo(-V(PEG)$_4$FTz$_{1\text{-}4}$A-); HSQC (green/blue) and HMBC (red) spectra

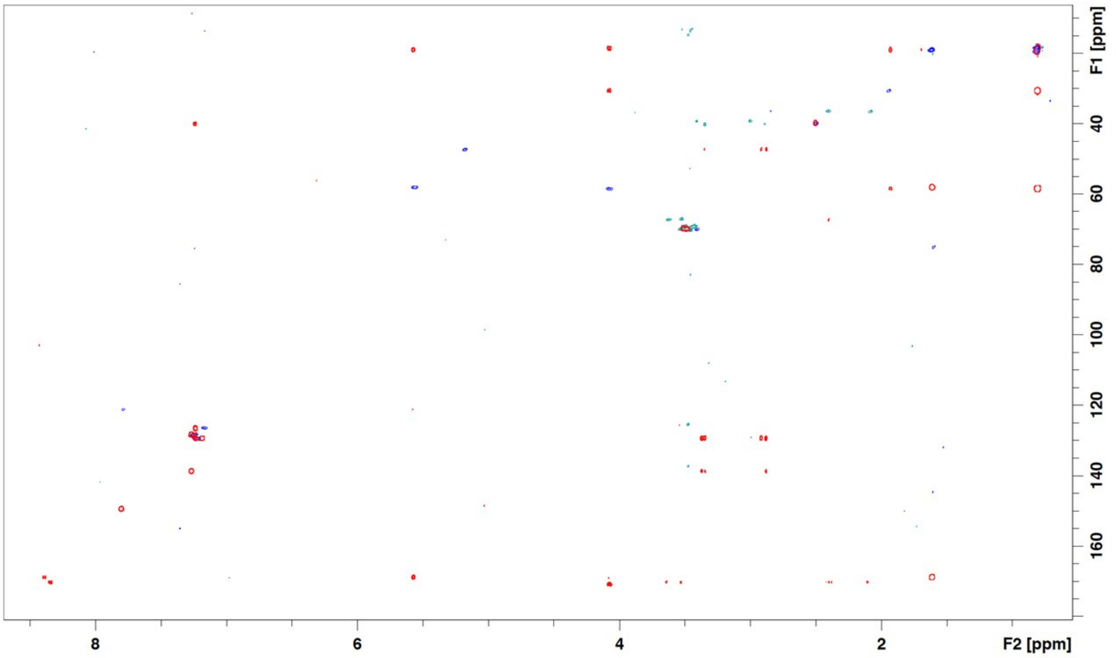

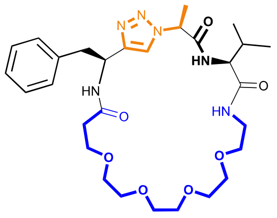

16c, 16d; Cyclo(-V(PEG)$_4$FTz$_{1\text{-}4}$A-); DMSO-d6; 700 MHz

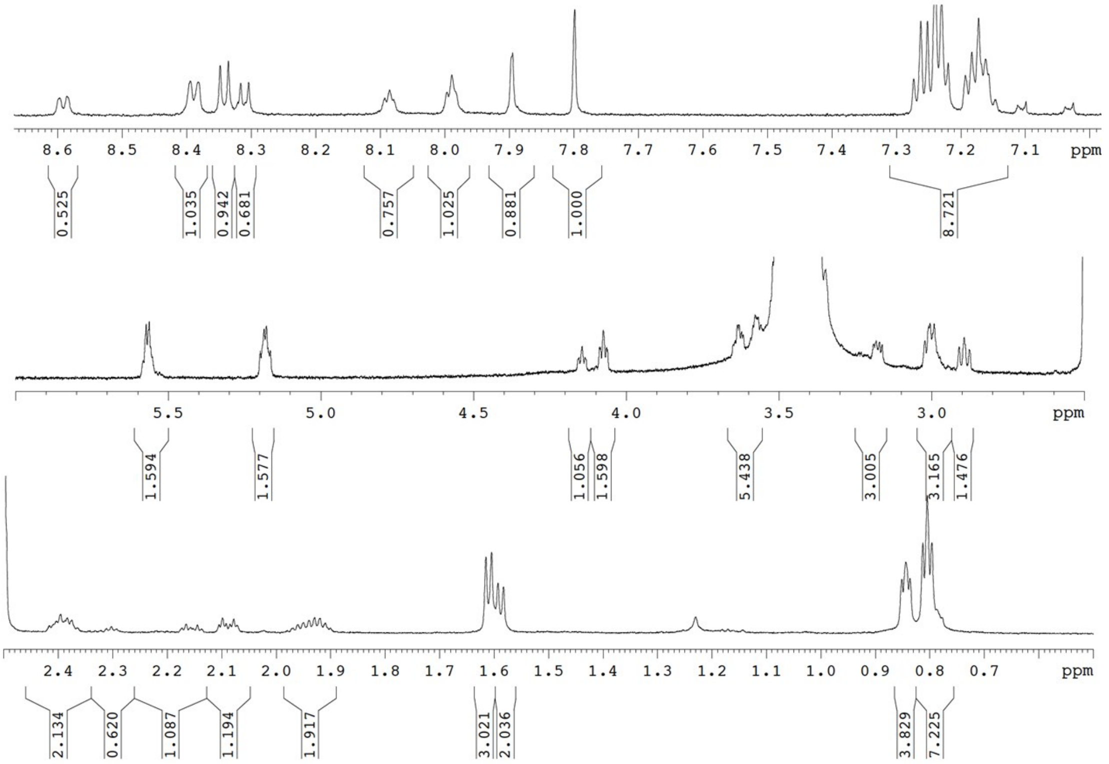

16c, 16d; Cyclo(-V(PEG)$_4$FTz$_{1\text{-}4}$A-); HSQC (green/blue) and HMBC (red) spectra

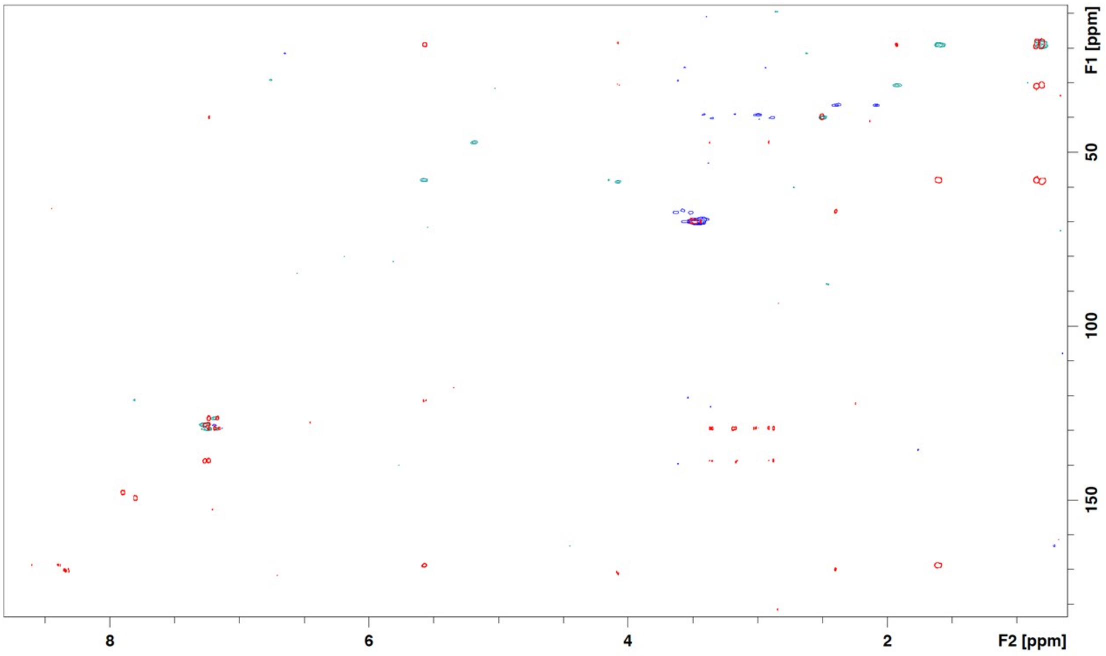

16c, 16d; Cyclo(-V(PEG)$_4$FTz$_{1\text{-}4}$A-); EXSY

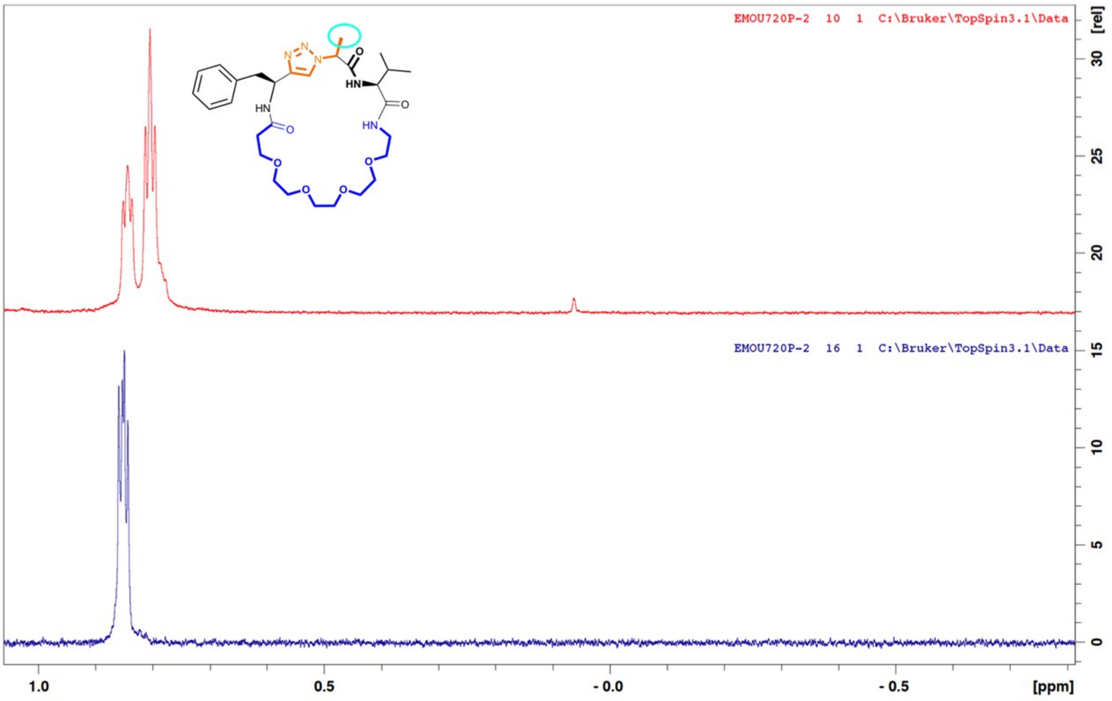

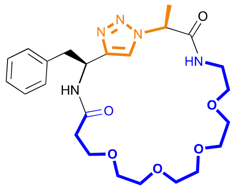

17c

17c; Cyclo(-(PEG)$_4$FTz$_{1\text{-}4}$A-); DMSO-d6; 700 MHz

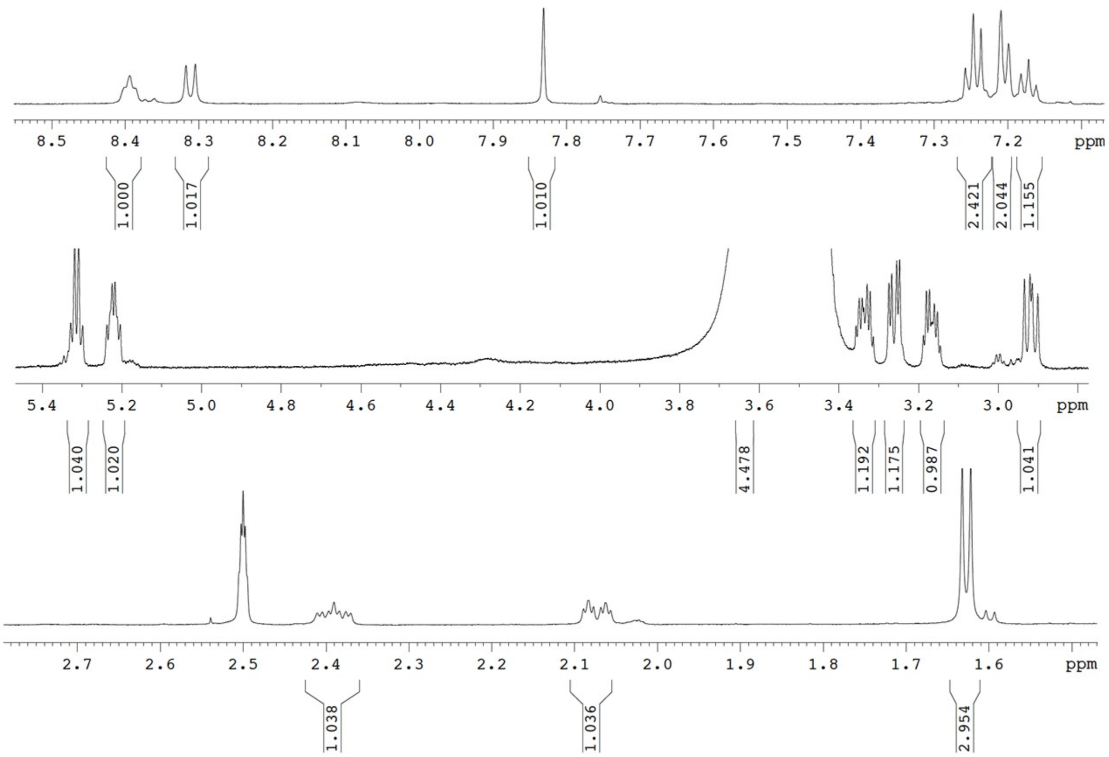

17c; Cyclo(-(PEG)$_4$FTz$_{1\text{-}4}$A-); HSQC (green/blue) and HMBC (red) spectra

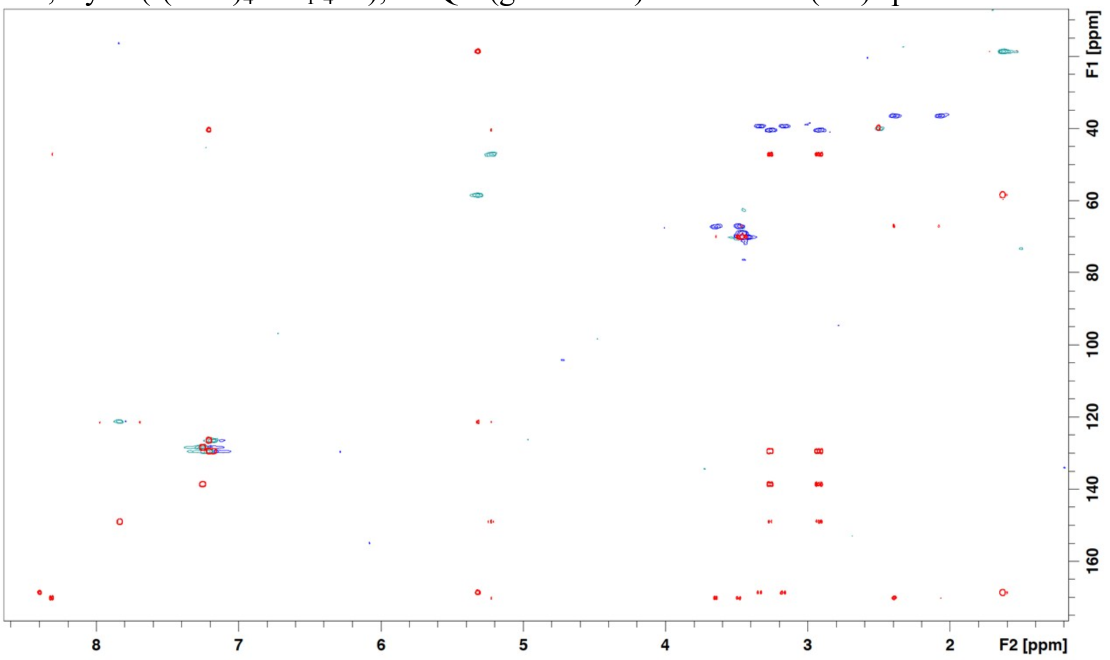

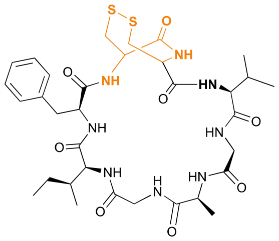

19c

19c; Cyclo (-VGAGIF[CC]-); DMSO-d6; 700 MHz

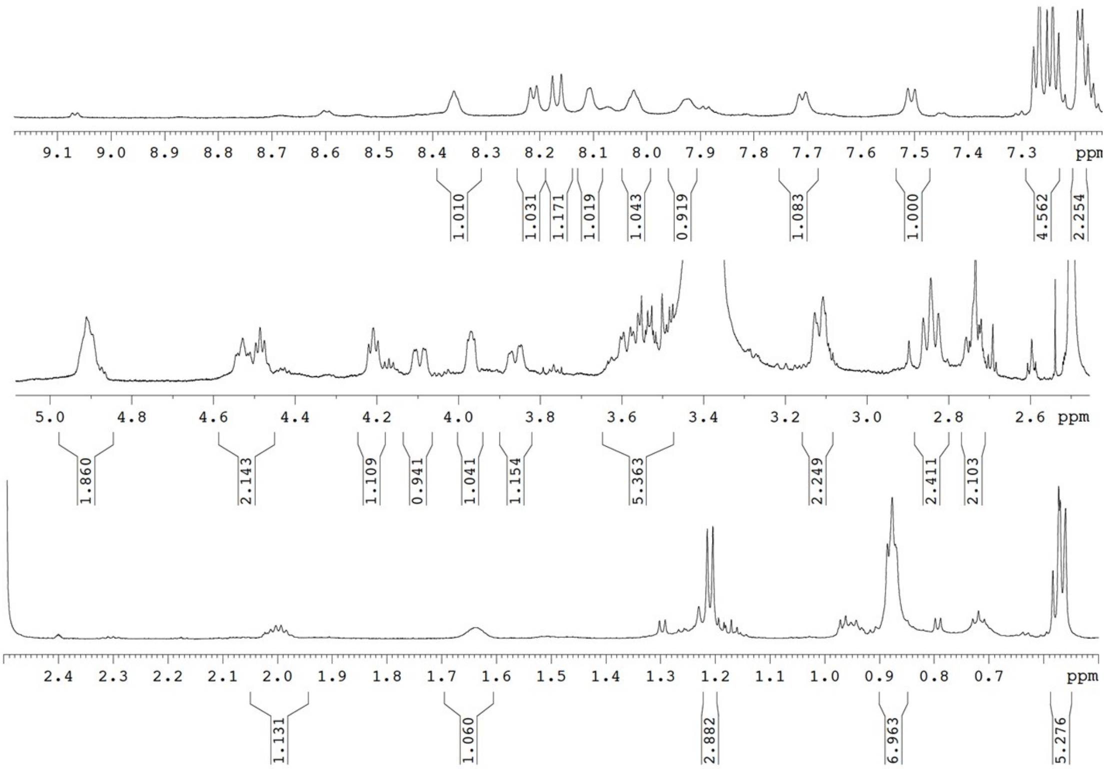

19c; Cyclo (-VGAGIF[CC]-); HSQC (green/blue) and HMBC (red) spectra

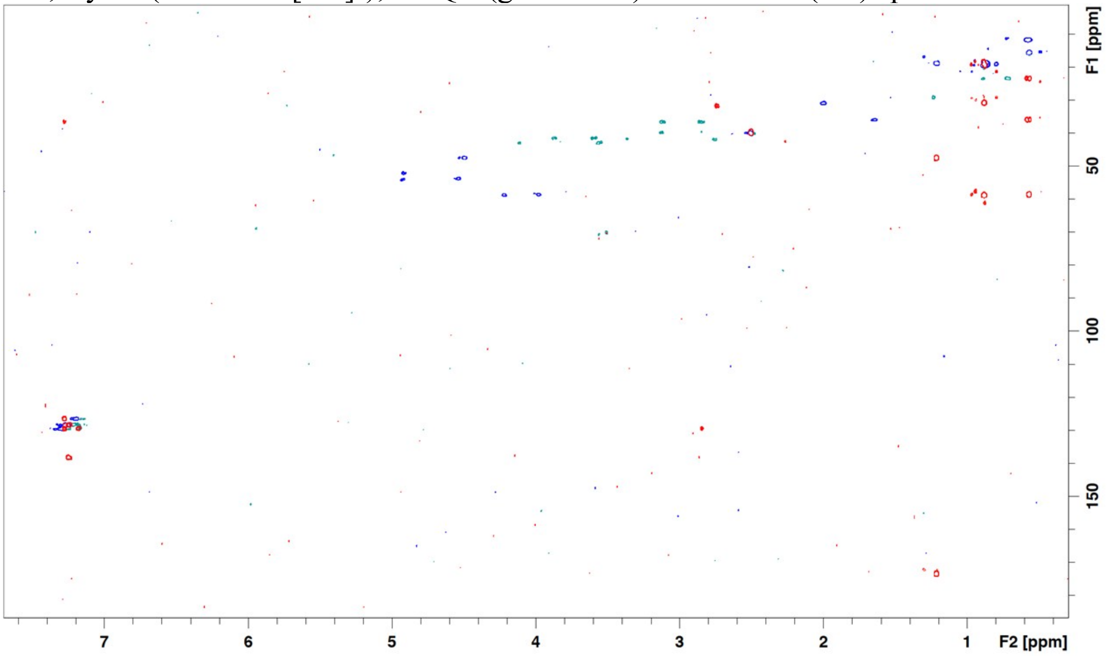

VI. MS-MS data of enzymatic reaction products of peptides 1-26

MS-MS fragmentation data of PatGmac products were acquired on the expected mass. The corresponding fragments and their theoretical masses are shown.
Cyclic peptides fragmentation pattern: The fragmentation can start at any point of the macrocycle Fragments containing both the *N*-terminal Val and the *C*-terminal Pro can only exist in the fragmentation pattern of cyclic peptide. CO loss is more common in cyclic peptides.
Linear peptides fragmentation pattern: Fragmentation can only start at the *N*- or *C*-terminal of the peptide. Fragments with both the *N*-terminal Val and the *C*-terminal Pro won't exist. One or two H_2O molecules loss is more common in linear peptides.

Expected theoretical masses in red; observed masses in black.

PatGmac reaction product of 7: Cyclo(-VGAGIGAP-); $[M+H]^+$=623.3

Fragment			$[M+H]^+$	$[M-H_2O+H]^+$	$[M-CO+H]^+$
VGAGIGAP			623.3511	605.3406	595.3562
			623.3499	605.3397	595.3556
-Ala	PVGAGIG		552.3140	534.3035	524.3196
			552.3133	534.3029	524.3151
	-Gly	PVGAGI	495.2926		467.2976
			495.2923		467.2972
	-Ile	PVGAG	382.2085		354.2136
			382.2088		354.2138
	-Gly	PVGA	325.1874		297.1921
			325.1879		297.1921
	-Ala	PVG	254.1499		226.1550
			254.1506		226.1546
	-Gly	PV	197.1285		169.1335
			197.1292		169.1343
-Ile	GAPVGAG		510.2671		482.2733
			510.2667		482.2719
-Val	-Pro	GAGIGA	427.2300	409.2194	399.2350
			427.2303	409.2195	399.2352

PatGmac reaction product of 11a: Cyclo(-VGAGIGFTz$_{1-4}$A-); $[M+H]^+$=697.4

Fragment			$[M+H]^+$	$[M-H_2O+H]^+$	$[M-CO+H]^+$
VGAGIGFTzA			697.3780	679.3675	669.3831
			697.3769	679.3669	669.3797
-Ile	GFTzAVGAG		584.2940		
			584.2952		
	-Gly	FTzAVGAG	527.2725		499.2776
			527.2726		499.2692
	-AlaGly	FTzAVG	399.2139		371.2190
			399.2121		371.2124
-FTzAV	GAGIG		356.1928		
			356.1907		
	-Gly	AGIG	299.1714		271.1765
			299.1687		271.1735
	-Ala	GIG	228.1343		
			228.1306		

PatGmac reaction product of 14: Cyclo(-VGAGIGATz$_{1-4}$A-); $[M+H]^+$=621.3

Fragment		$[M+H]^+$	$[M-H_2O+H]^+$	$[M-CO+H]^+$	$[M-N_2+H]^+$
VGAGIGATzA		621.3467	603.3362	593.3518	
		621.3474	603.3368	593.3494	
	$-N_2$		575.3300	565.3457	
			575.3347	565.3473	
-Gly					536.3191
					536.3224
-Ile	GATzAVGAG	508.2627		480.2677	
		508.2644		480.2636	
-ATzAV	GAGIG	356.1928			
		356.1924			
-GIGATzA	VGA	228.1343			
		228.1320			

PatGmac reaction product of 15: Cyclo(-ITATz$_{1-4}$AITFTz$_{1-4}$A-); $[M+H]^+$=837.4

Fragment		$[M+H]^+$	$[M-H_2O+H]^+$	$[M-CO+H]^+$	$[M-N_2+H]^+$
ITATzAITFTzA		837.4730	819.4624	809.4781	
		837.4834	819.4684	809.4734	
$-N_2$				781.4719	
				781.4765	
-Thr	ATzAITFTzAI			708.4304	708.4192
					708.4237
	-CO				680.4242
					680.4249
-ATzAI	TFTzAIT	558.3035			
		558.3050			
-FTzAI	TATzAIT	482.2722			
		482.2738			
-ITATzA	ITFTzA	457.2558		429.2609	429.2496
		457.2569			429.2522
-ITFTzA	ITATzA	381.2245		353.2296	353.2183
		381.2233			353.2207
	ATzAI				252.1707
					252.1693
	FTzA				215.1179
					215.1158
	ATzA			139.0978	139.0866
					139.0838

PatGmac reaction product of 16: Cyclo(-V-PEG$_4$-FTz$_{1-4}$A-); $[M+H]^+$= 589.3

Fragment		$[M+H]^+$	$[M-H_2O+H]^+$	$[M-CO+H]^+$
V-PEG$_4$-FTzA		589.3344		561.3395
		589.3351		561.3382
$-CO(CH_2)_2O(CH_2)_2$		490.2898		
		490.2903		
	PEG$_4$-NH_3^+	265.1758		
		265.1760		
	FTzA-N_2	215.1179		
		215.1179		

PatGmac reaction product of 17: Cyclo(PEG$_4$-FTz$_{1-4}$A-); $[M+H]^+$= 490.3

Fragment		$[M+H]^+$	$[M-H_2O+H]^+$	$[M-CO+H]^+$	$[M-N_2+H]^+$
PEG$_4$-FTzA		490.2660		462.2711	462.2599
		490.2667			462.2602
	-H_2O				444.2493
					444.2502
-CO(CH$_2$)$_2$O(CH$_2$)$_2$		391.2214			
		391.2232			

PatGmac reaction product of 18: Cyclo(PEG$_4$-ATz$_{1-4}$A-); $[M+H]^+$= 414.2

Fragment		$[M+H]^+$	$[M-H_2O+H]^+$	$[M-CO+H]^+$	$[M-N_2+H]^+$
PEG$_4$-FTzA		414.2347			386.2286
		414.2361			386.2299
	-H_2O				368.2292
					368.2193
-CO(CH$_2$)$_2$O(CH$_2$)$_2$		315.1901			
		315.1918			
	- O(CH$_2$)$_2$	271.1639			
		271.1651			
(PEG)4		248.1492			
		248.1485			

PatGmac reaction product of 19: Cyclo(-VGAGIF[CC]-); $[M+H]^+$= 749.4

Fragment			$[M+H]^+$	$[M-H_2O+H]^+$	$[M-CO+H]^+$
VGAGIF[CC]			749.3109	731.3004	721.3160
			749.3097		721.3153
-Ile	F[CC]VGAG		636.2269		
			636.2272		
		F[CC]	352.0784		
			352.0807		
		F			120.0808
					120.0837
-Phe	[CC]VGAGI		602.2425		574.2476
			602.2432		574.2483
	-Ile	[CC]VGAG	489.1585		
			489.1604		
-Val	-[CC]	GAGIF	446.2398		
			446.2414		
	-Phe	GAGI	299.1714		271.1765
			299.1743		271.1793
		GI			143.1179
					143.1208

PatGmac reaction product of 19: Linear VGAGIF[CC]; $[M+H]^+$= 767.4

Fragment		$[M+H]^+$	$[M-H_2O+H]^+$	$[M-CO+H]^+$
H-VGAGIF[CC]-OH		767.3215		
		767.3225		
-Ile	VGAGIF		545.3082	

					545.3097	
	-Phe	VGAGI			398.2398	
					398.2424	
		-CO				370.2449
						370.2482
-VGAGI				370.0890		
				370.0916		

PatGmac reaction product of 20: Linear VPAPIP[CC]; $[M+H]^{+}$= 797.4

**Fragment**				**$[M+H]^{+}$**	**$[M\text{-}H_2O+H]^{+}$**	**$[M\text{-}CO+H]^{+}$**
H-VPAPIP[CC]-OH				797.3684		
				797.3700		
-Val	PAPIP[CC]			698.3000		
				698.3019		
	-Pro	APIP[CC]		601.2473		
				601.2493		
	-Ala	PIP[CC]		530.2102		
				530.2132		
		P[CC]		320.0733		
				320.0766		
-P[CC]	VPAPI				478.3024	
					478.3060	
		-CO				450.3075
						450.3107
	-Val	PAPI			379.2340	
					379.2373	
		-CO				351.2391
						351.2417
	-Ile	PAP			266.1499	
					266.1528	
		PI			211.1441	
					211.1465	
	-Ile	VPAP			365.2183	
					365.2215	
		-Phe	VPA		268.1656	
					268.1684	
		-Val	PA		169.0972	
					169.0997	

PatGmac reaction product of 22: Linear FyKT[CC]; $[M+H]^{+}$= 762.3

**Fragment**				**$[M+H]^{+}$**	**$[M\text{-}H_2O+H]^{+}$**	**$[M\text{-}CO+H]^{+}$**
H-FyKT[CC]-OH				762.2949	744.2844	
				762.3021	744.2892	
-Phe	yKT[CC]			615.2265		
				615.2302		
	-Tyr	KT[CC]		452.1632	434.1532	
				452.1641	434.1528	
		-[CC]	KT		230.1499	
					230.1470	
		-T	K		129.1022	

				129.0981
-[CC]	FyKT			540.2822
				540.2838
	-Thr	FyK		439.2345
				439.2342
		-Lys	Fy	311.1396
				311.1371
			Ky	292.1656
				292.1635

PatGmac reaction product of 24: Cyclo(-LGKYG[CC]-); $[M+H]^+ = 723.3$

Fragment				$[M+H]^+$	$[M\text{-}H_2O+H]^+$	$[M\text{-}CO+H]^+$
LGKYG[CC]				723.2953	705.2847	695.3004
				723.2979		695.3033
-Gly	KYG[CC]L				648.2632	
					648.2656	
-Lys	YG[CC]LG			595.2003		567,2054
				595.2027		567.2084
	-Gly		YG[CC]L	538.1789		510.1839
				538.1818		510.1871
	-Leu		YG[CC]	425.0948		
				425.0974		
-Tyr	-Lys	-Gly	G[CC]L	375.1155		
				375.1179		
			LGK	299.2078		
				299.2103		
	-Leu		GK	186.1237		
				186.1257		
	-Gly		K	129.1022		
				129.1039		
			Y			136.0757
						136.0774

PatGmac reaction product of 25: Cyclo(-GYKLG[CC]-); $[M+H]^+ = 723.3$

Fragment				$[M+H]^+$	$[M\text{-}H_2O+H]^+$	$[M\text{-}CO+H]^+$
GYKLG[CC]				723.2953	705.2847	695.3004
				723.3015		695.3058
-Gly	[CC]GYKL				648.2632	
					648.2668	
	-Leu -NH		[CC]GYK	538.1789		510.1839
				538.1810		510.1853
	-Lys		[CC]GY	425.0948		
				425.0948		
	-[CC]		GY	221.0921		
				221.0880		
-Lys	LG[CC]GY			595.2003		567,2054
				595.2033		567.2084
	-Tyr		LG[CC]G	432.1370		
				432.1374		
	-Gly		LG[CC]	375.1155		347.1206
				375.1144		347.1197
-Tyr	-Lys	-Gly	G[CC]L	375.1155		

				375.1179		
			KLG	299.2078		
				299.2058		
			K	129.1022		
				129.0981		
			Y			136.0757
						136.0716
			LG-NH	186.1237		
				186.1199		

PatGmac reaction product of 26: Cyclo(-LGYKLG[CC]-); $[M+H]^{+}$= 836.4

Fragment				$[M+H]^{+}$	$[M-H_2O+H]^{+}$	$[M-CO+H]^{+}$
LGYKLG[CC]				836.3793	818.3688	808.3844
				836.3817		808.3860
-Gly	[CC]LGYKL				761.3473	
					761.3489	
	-Leu	-Lys	[CC]LGY	538.1789		510.1839
				538.1806		510.1871
	-Tyr		[CC]LG	375.1155		
				375.1179		
-Lys	LG[CC]LGY			708.2844		680.2895
				708.2868		680.2920
-Leu	G[CC]LGYK			723.2953		
				723.2988		
	-Lys		G[CC]LGY	595.2003		567,2054
				595.2026		567.2080
	-Tyr		G[CC]LG	432.1370		
				432.1402		
	-Gly		G[CC]L	375.1155		
				375.1179		
			YK	292.1656		
				292.1677		
			K	129.1022		
				129.1038		
			Y			136.0757
						136.0775

VII. Maldi-MS traces of the PatGmac reactions of peptides 19 and 20

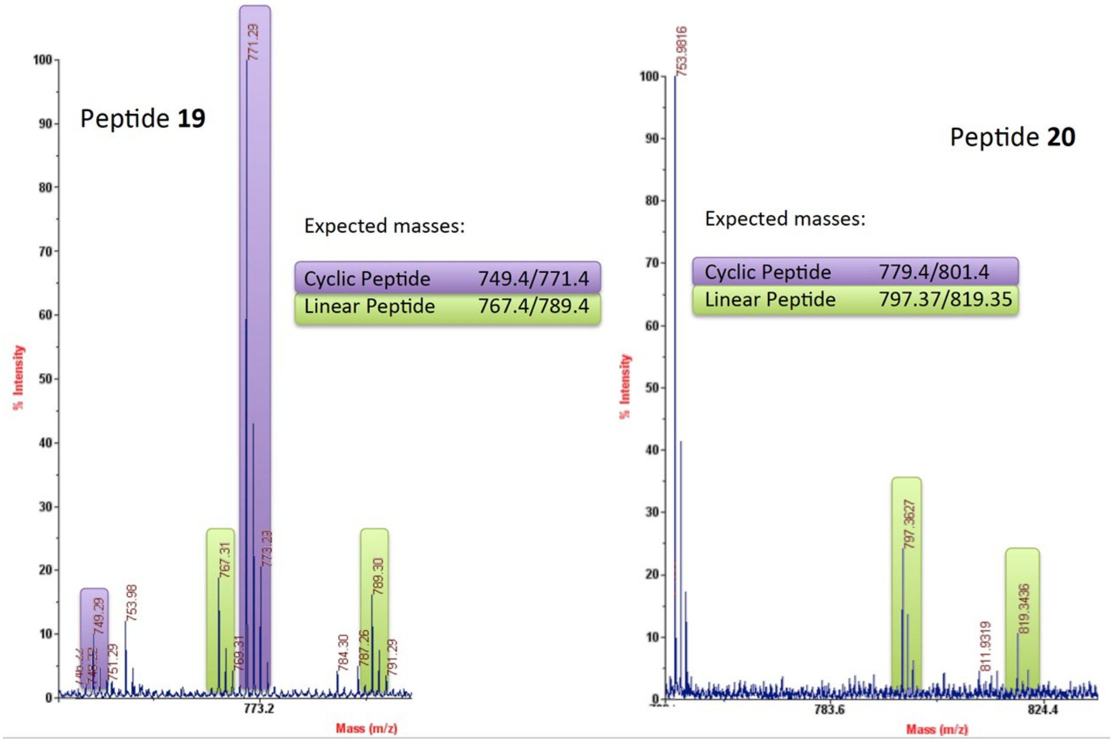

Figure S3: Maldi-MS trace after 2 days incubation of peptides **19** and **20** in the presence of PatGmac, showing expected masses of cyclic and linear products and highlighting the masses of the observed products.

VIII. LC-MS traces of final cyclic peptides

For each of the cyclic hybrid peptides **1-7**, the UV trace at 220 nm obtained by HPLC is complemented by its corresponding LCMS trace at the desired molecular weight (Single Ion Monitoring SIM mode) The HPLC methods used are described in section II.

Cyclic peptide **11c**; Cyclic peptide **11d;** Cyclo (-VGAGIG**FTz**$_{1-4}$**A**-), system A1

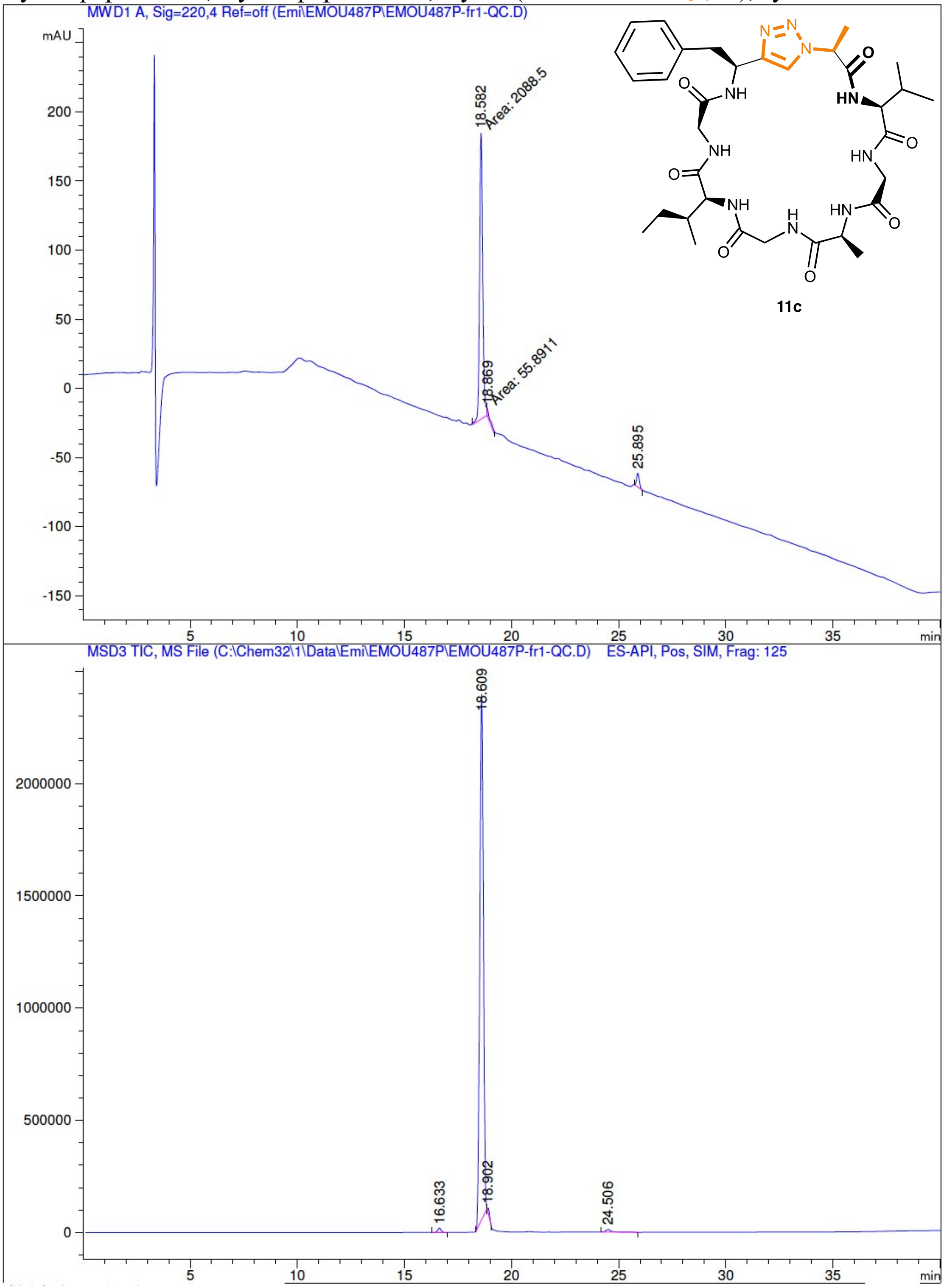

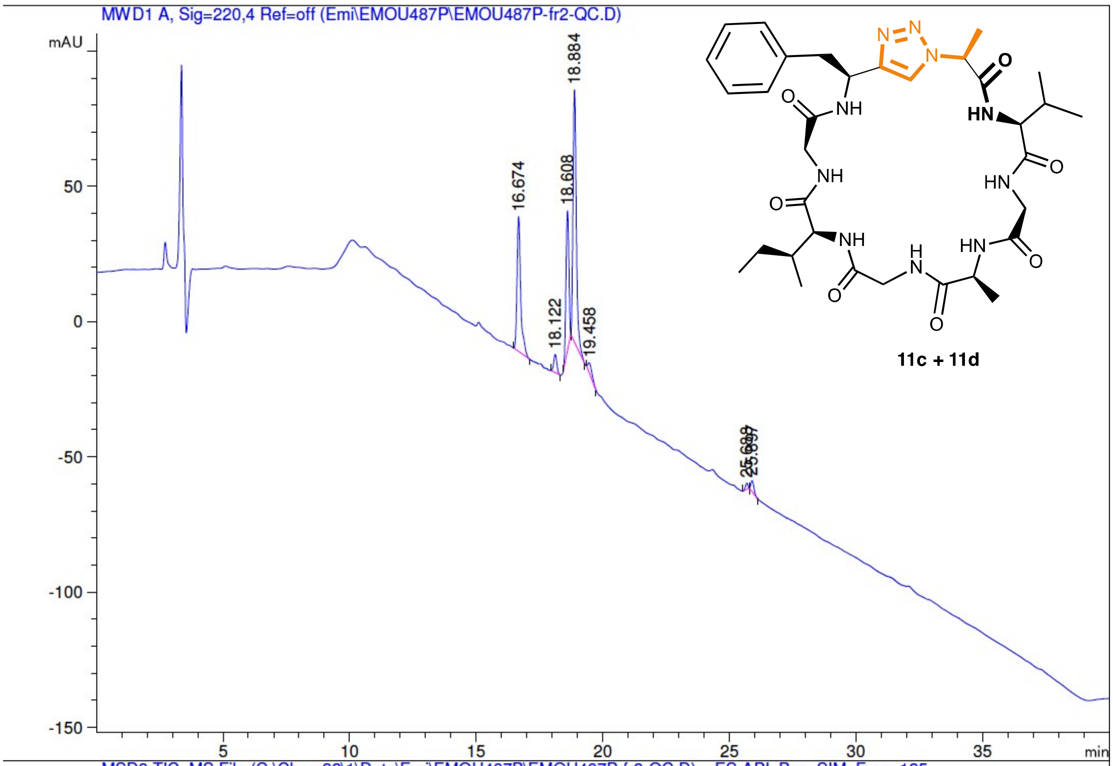
MWD1 A, Sig=220,4 Ref=off (Emi\EMOU487P\EMOU487P-fr2-QC.D)
mAU
16.674
18.122
18.608
18.884
19.458
11c + 11d
min

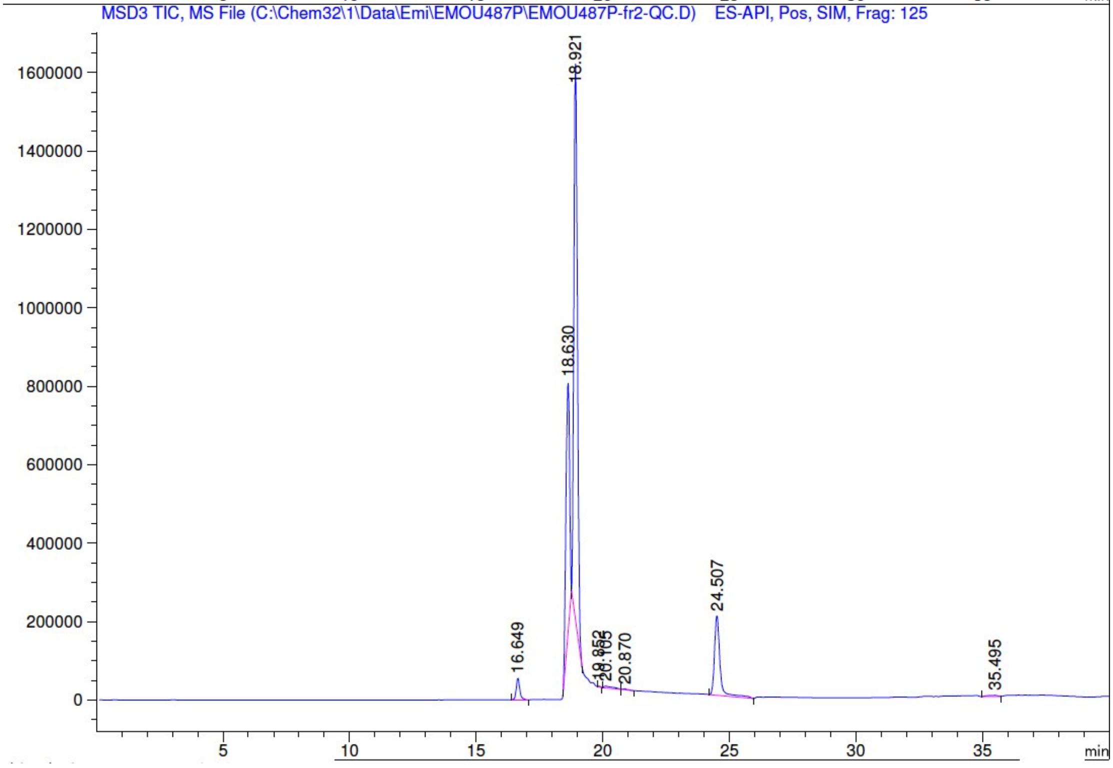
MSD3 TIC, MS File (C:\Chem32\1\Data\Emi\EMOU487P\EMOU487P-fr2-QC.D) ES-API, Pos, SIM, Frag: 125
18.921
18.630
16.649
20.870
24.507
35.495
min

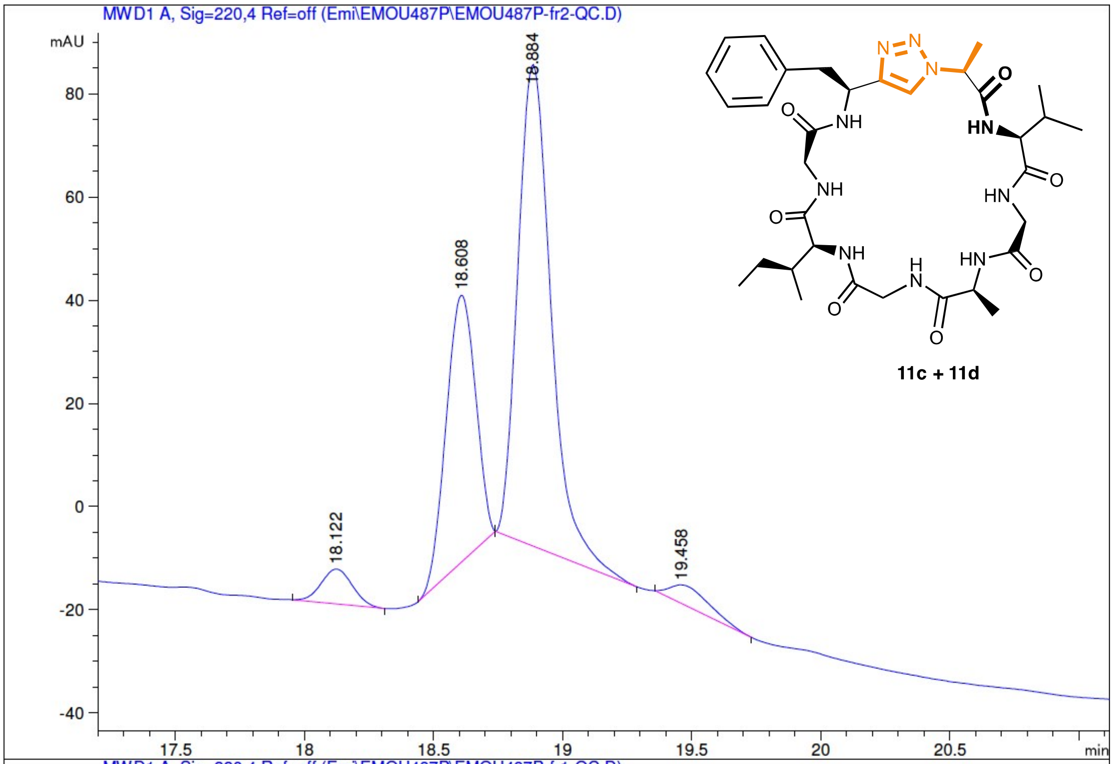
MWD1 A, Sig=220,4 Ref=off (Emi\EMOU487P\EMOU487P-fr2-QC.D)
mAU
80
60
40
20
0
-20
-40
18.122
18.608
18.884
19.458
17.5
18
18.5
19
19.5
20
20.5
min
11c + 11d

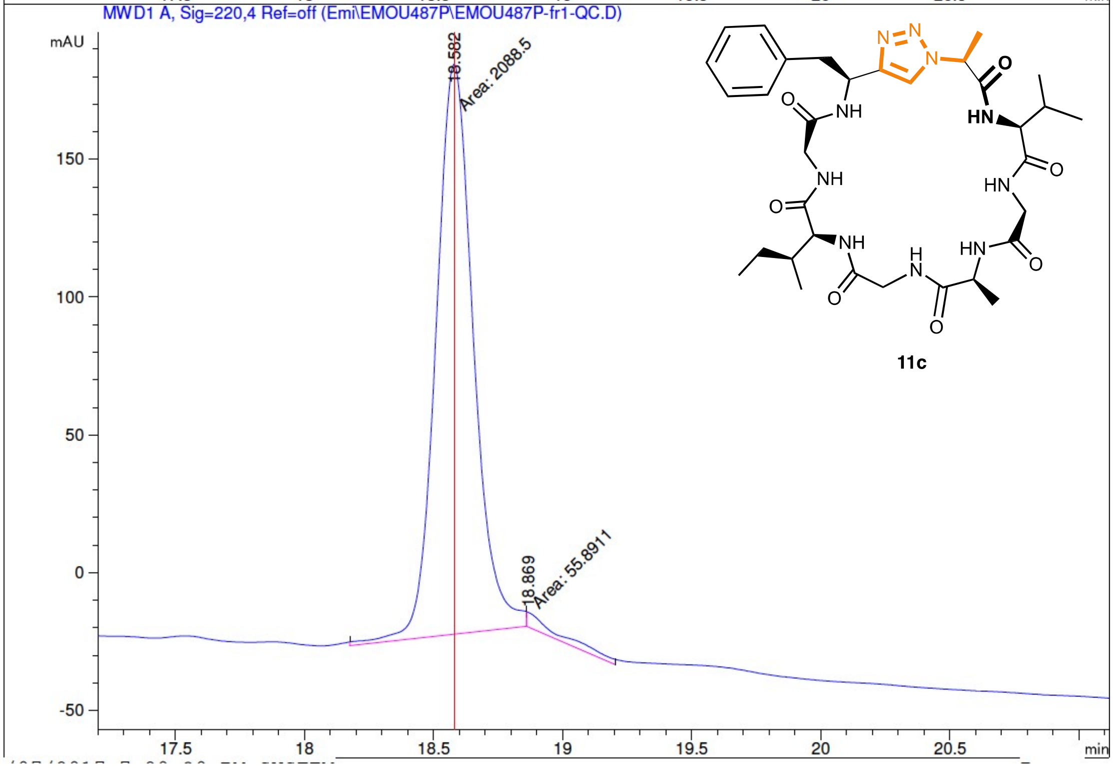
MWD1 A, Sig=220,4 Ref=off (Emi\EMOU487P\EMOU487P-fr1-QC.D)
mAU
150
100
50
0
-50
18.582
Area: 2088.5
18.869
Area: 55.8911
17.5
18
18.5
19
19.5
20
20.5
min
11c

Cyclic peptide **14c**; Cyclo (-VGAGIGA**Tz**$_{1\text{-}4}$**A**-), system A1

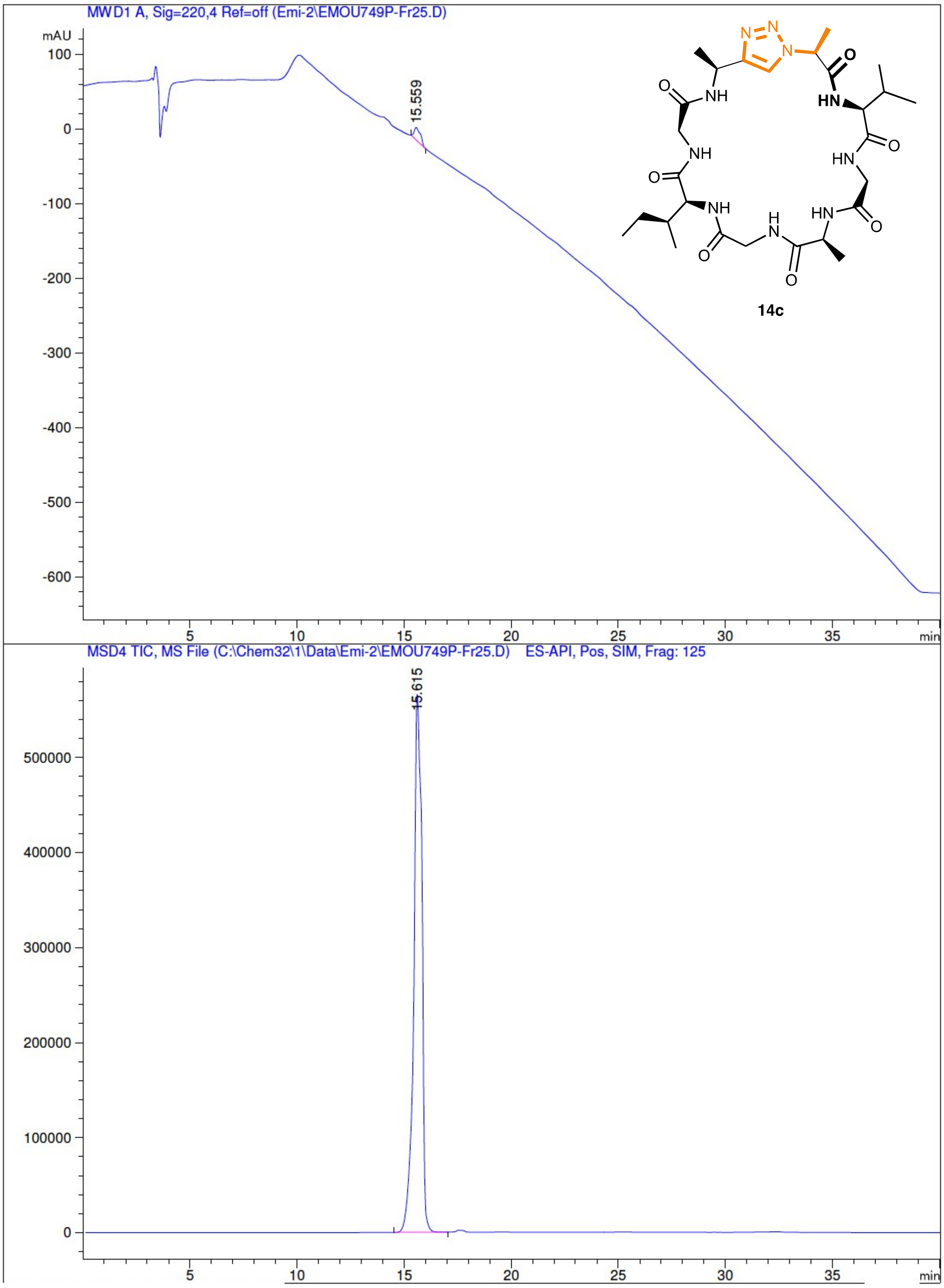

Cyclic peptide **16c**; Cyclic peptide **16d**; Cyclo (-V-PEG$_4$-**FTz**$_{1\text{-}4}$**A**-), system A1

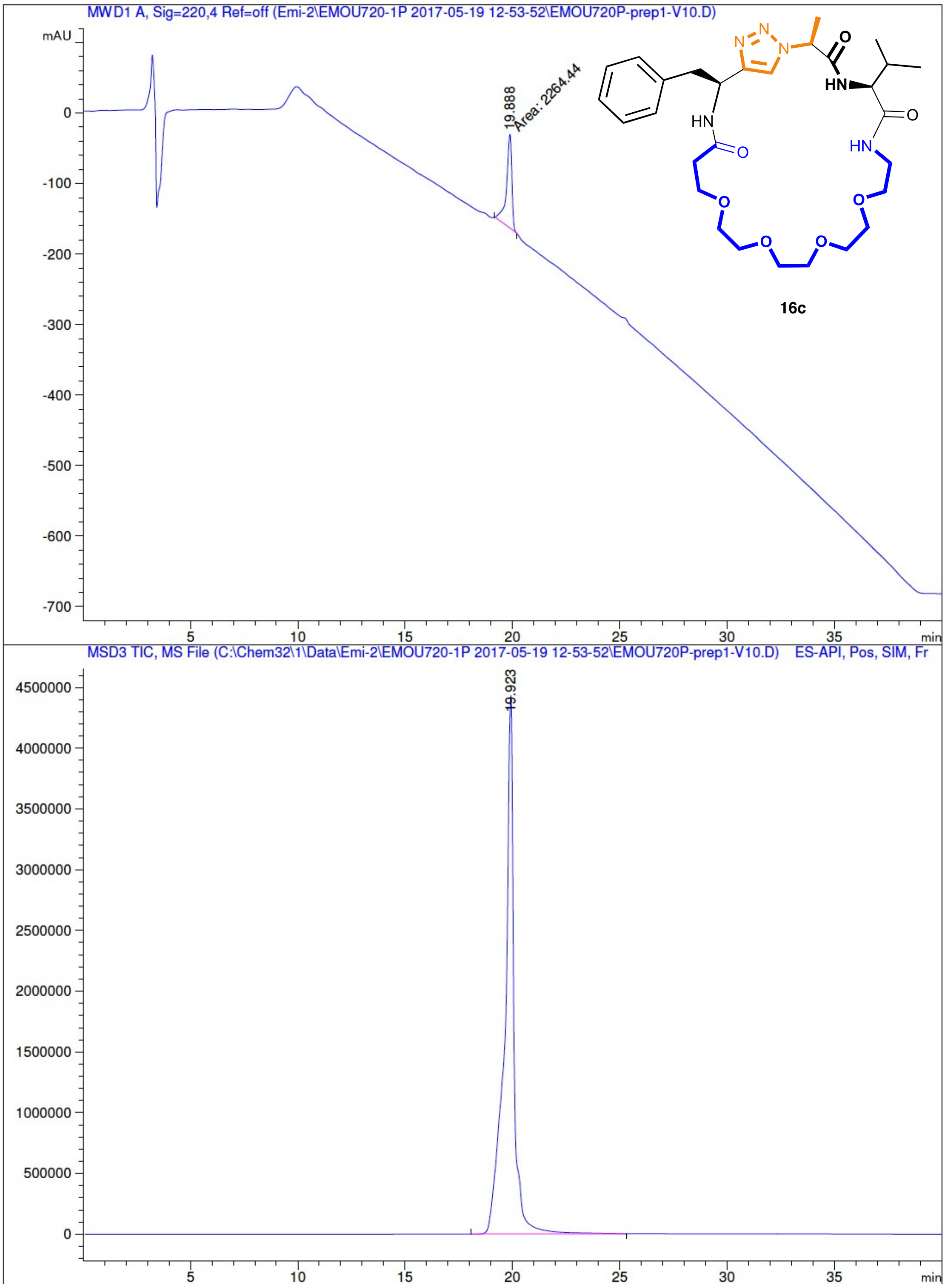

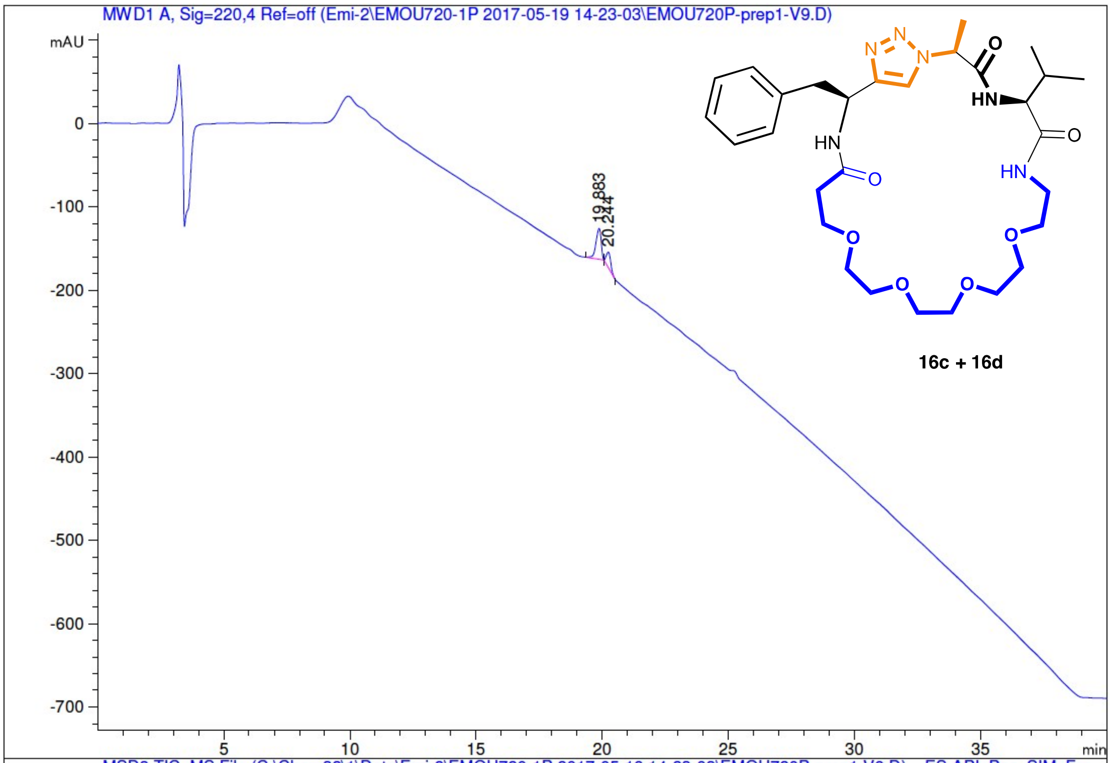
MWD1 A, Sig=220,4 Ref=off (Emi-2\EMOU720-1P 2017-05-19 14-23-03\EMOU720P-prep1-V9.D)
mAU
0
-100
-200
-300
-400
-500
-600
-700
5
10
15
20
25
30
35
min
19.883
20.244
16c + 16d

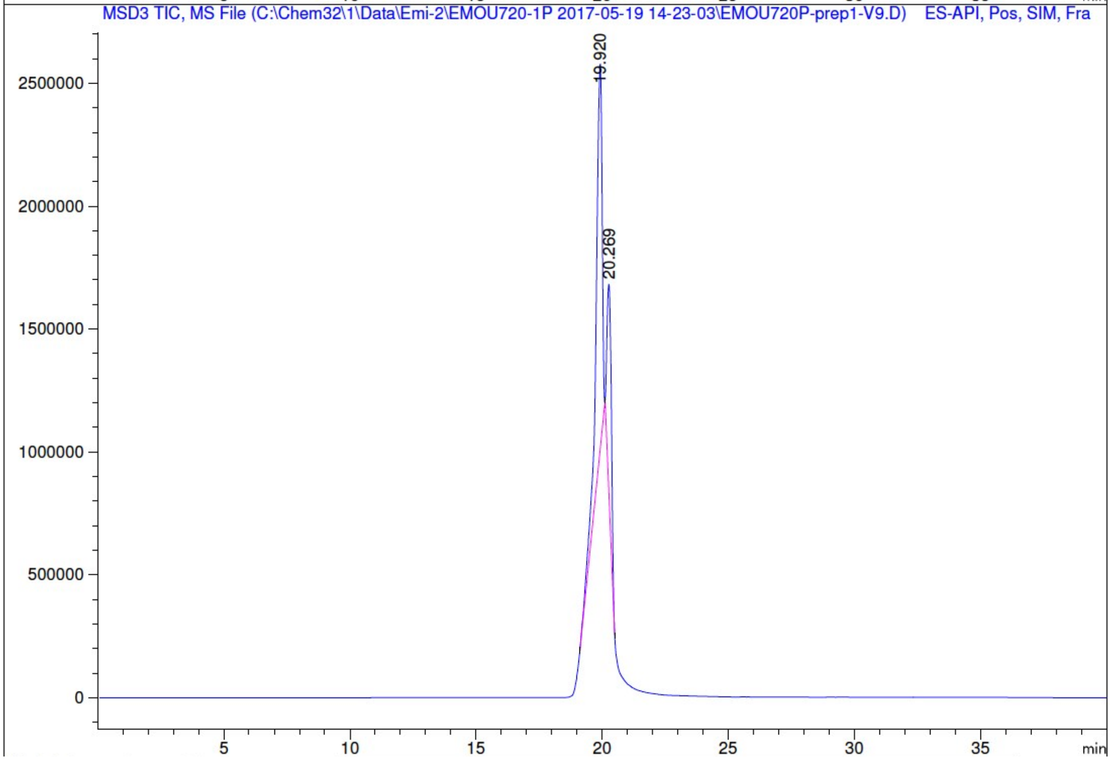
MSD3 TIC, MS File (C:\Chem32\1\Data\Emi-2\EMOU720-1P 2017-05-19 14-23-03\EMOU720P-prep1-V9.D) ES-API, Pos, SIM, Fra
2500000
2000000
1500000
1000000
500000
0
5
10
15
20
25
30
35
min
19.920
20.269

Cyclic peptide **17c**; Cyclo (-PEG$_4$-**F**$\mathbf{Tz}_{1\text{-}4}$**A**-), system A1

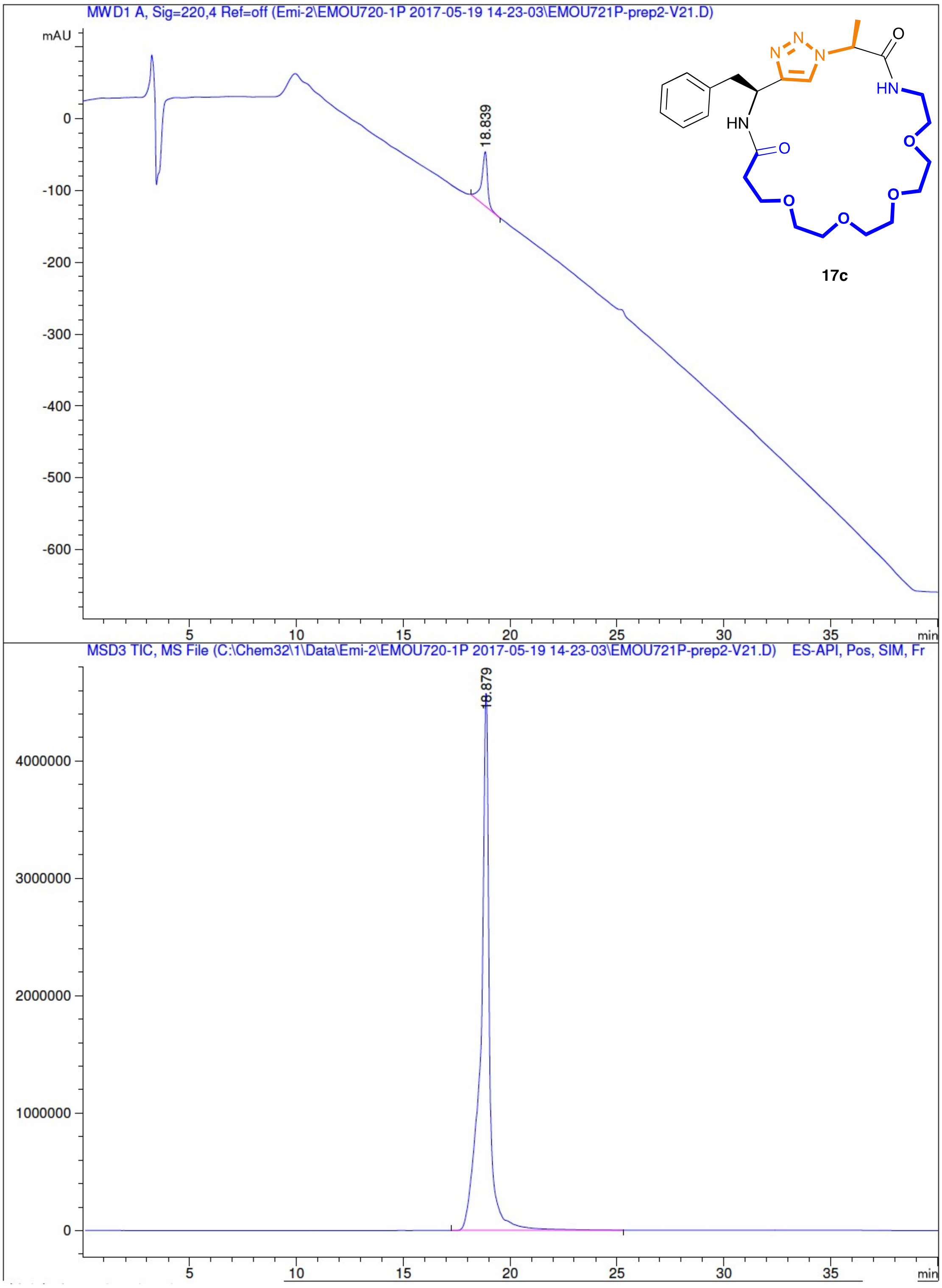

Cyclic peptide **19c**; Cyclo (-VGAGIF[**CC**]-), system A1

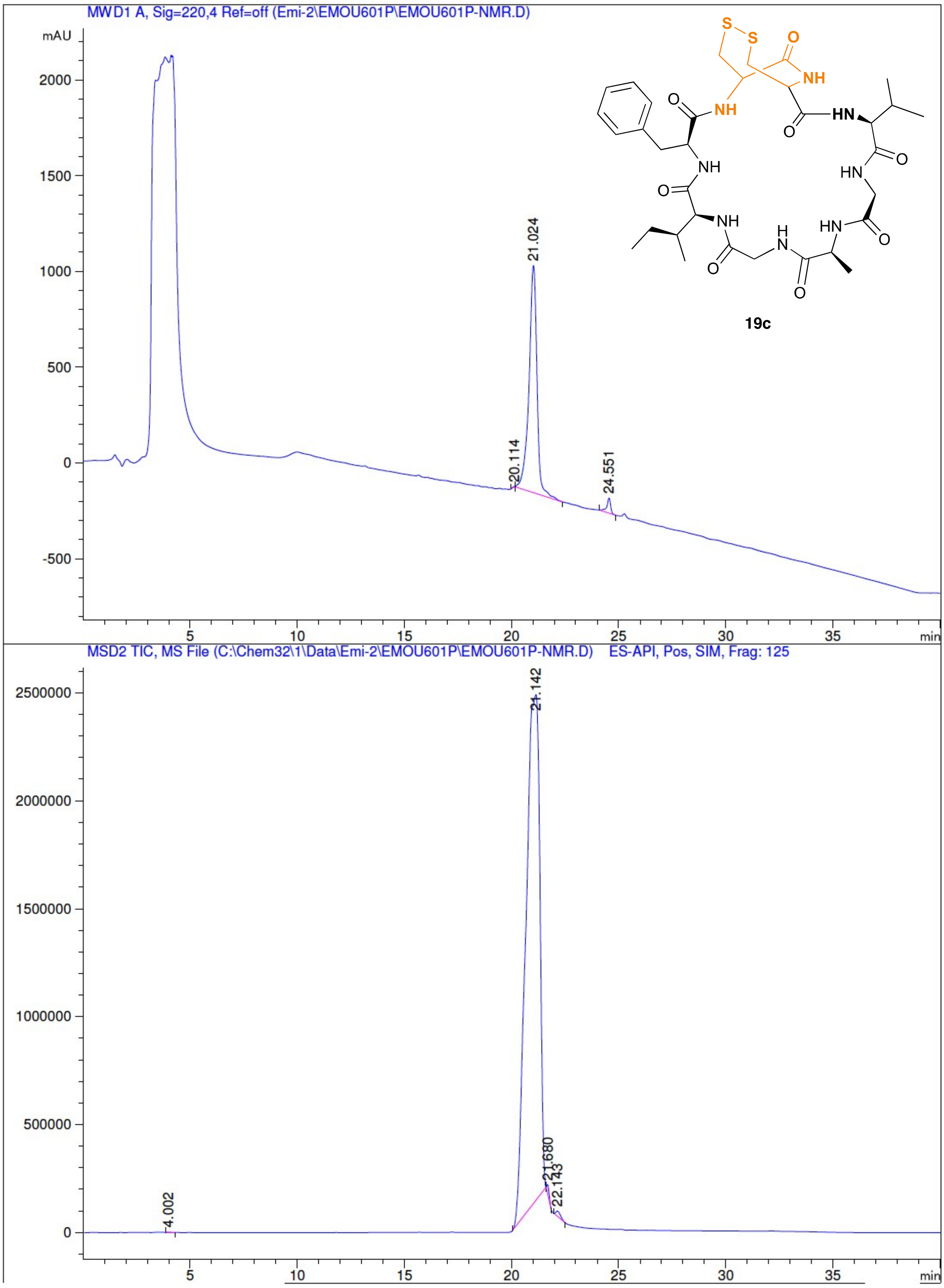

Cyclic peptide **19d**; Cyclo (-VGAGIFCC-), system A1

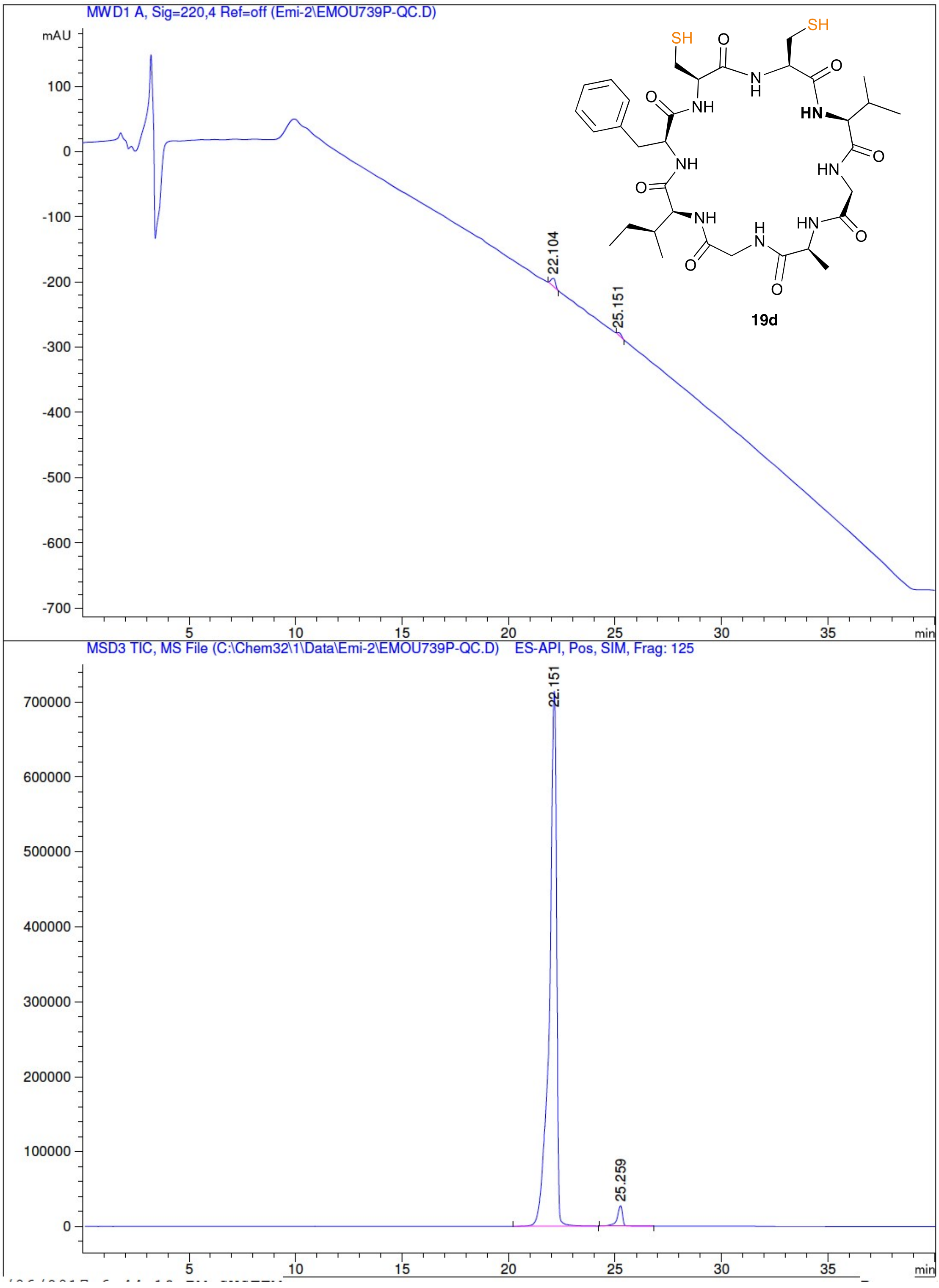

IX. LC-MS traces of starting hybrid peptides 1-26

For each of the precursor peptides **1-26,** the UV trace at 220 nm obtained by HPLC is complemented by its corresponding LCMS trace at the desired molecular weight (Single Ion Monitoring SIM mode). The HPLC methods used are described in section II.

Peptide **1**; VAGIGF**GTz**$_{1\text{-}4}$**A**AYD-NH_2, system A1

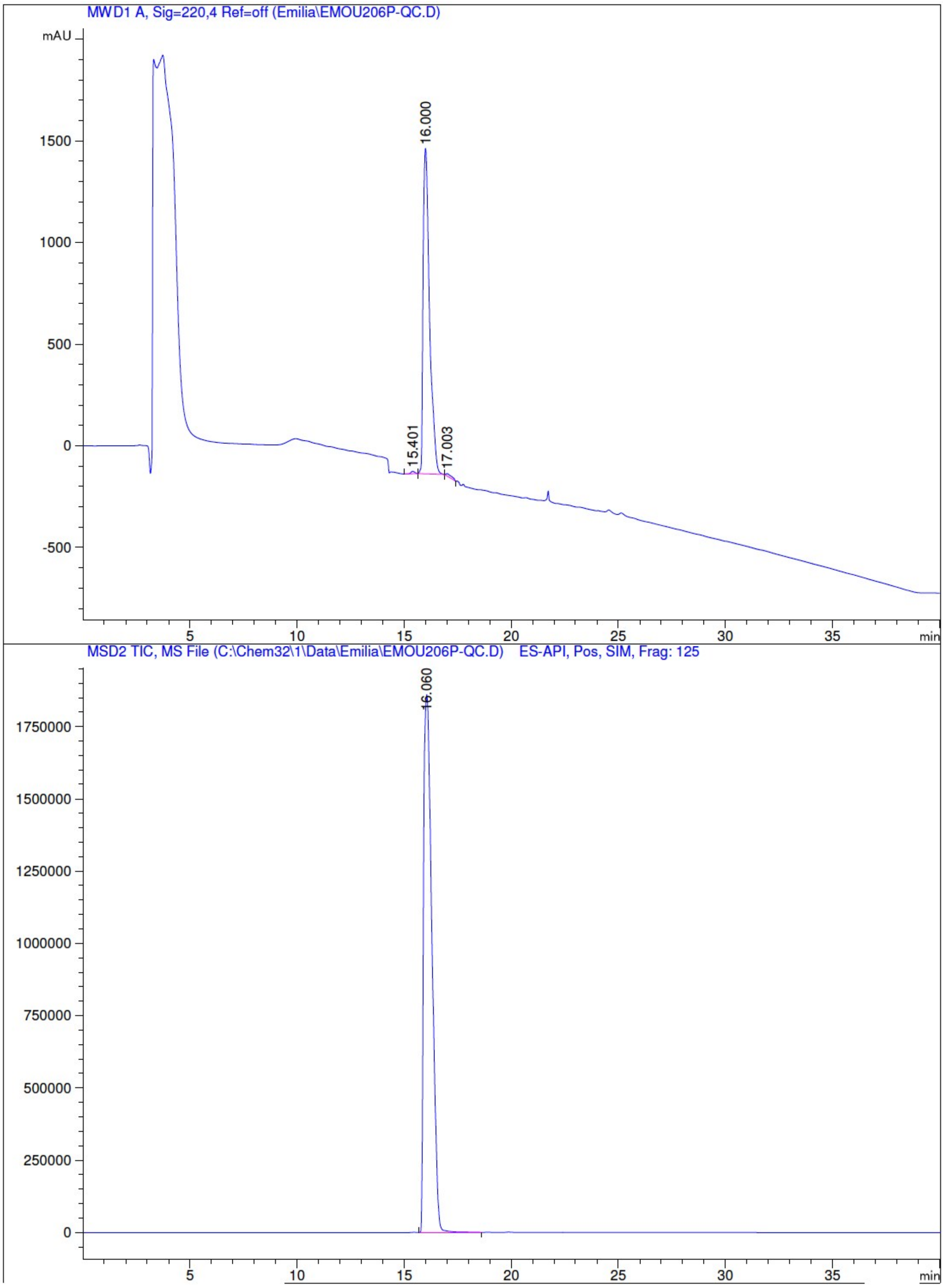

Peptide **2**; VGAGAI**GTz**$_{1\text{-}4}$**A**AYD -NH$_2$, system A1

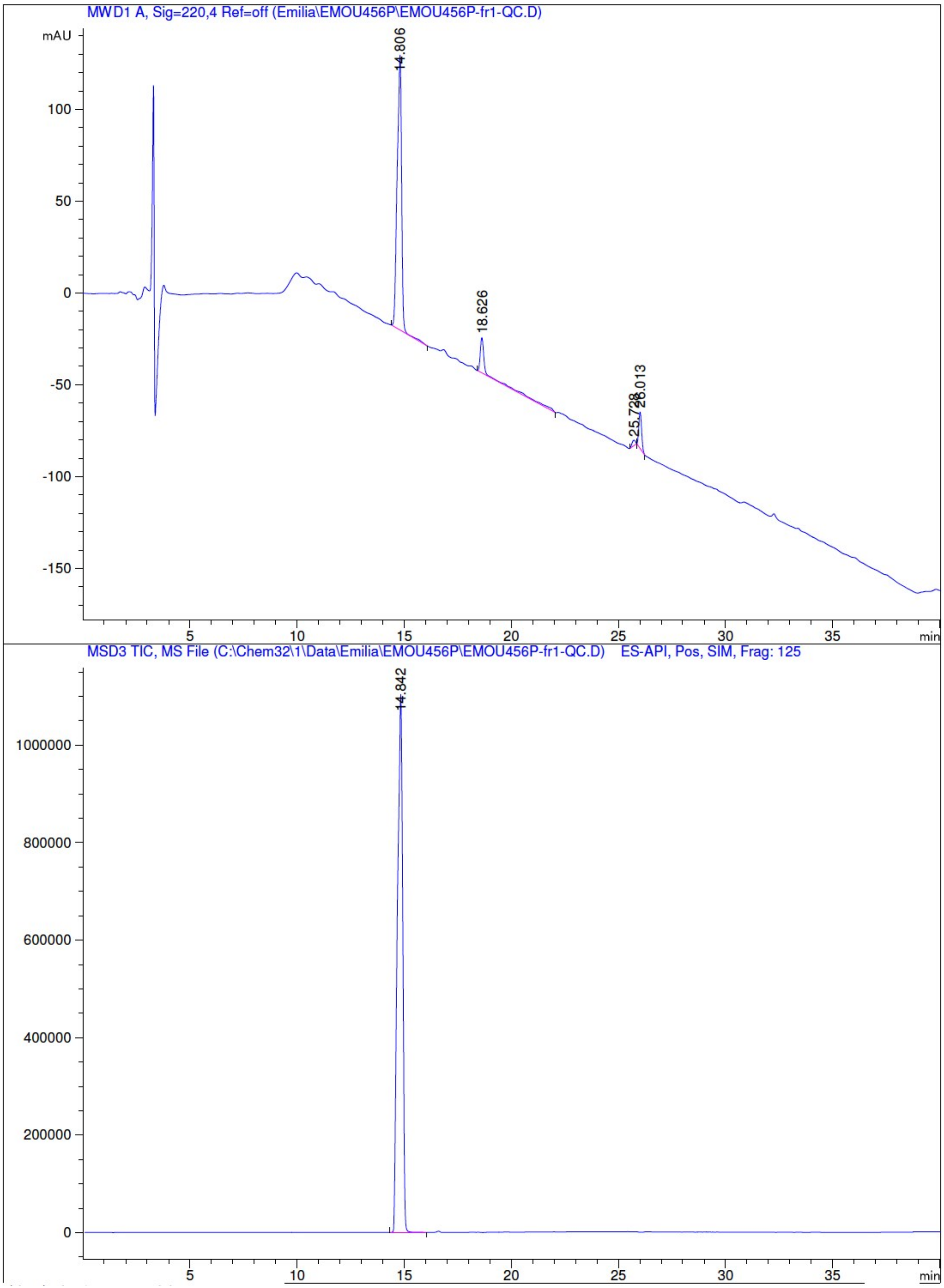

Peptide **3**; VGAGIGF**GTz**$_{1\text{-}4}$**A**<u>AYD</u>-NH_2, system A1

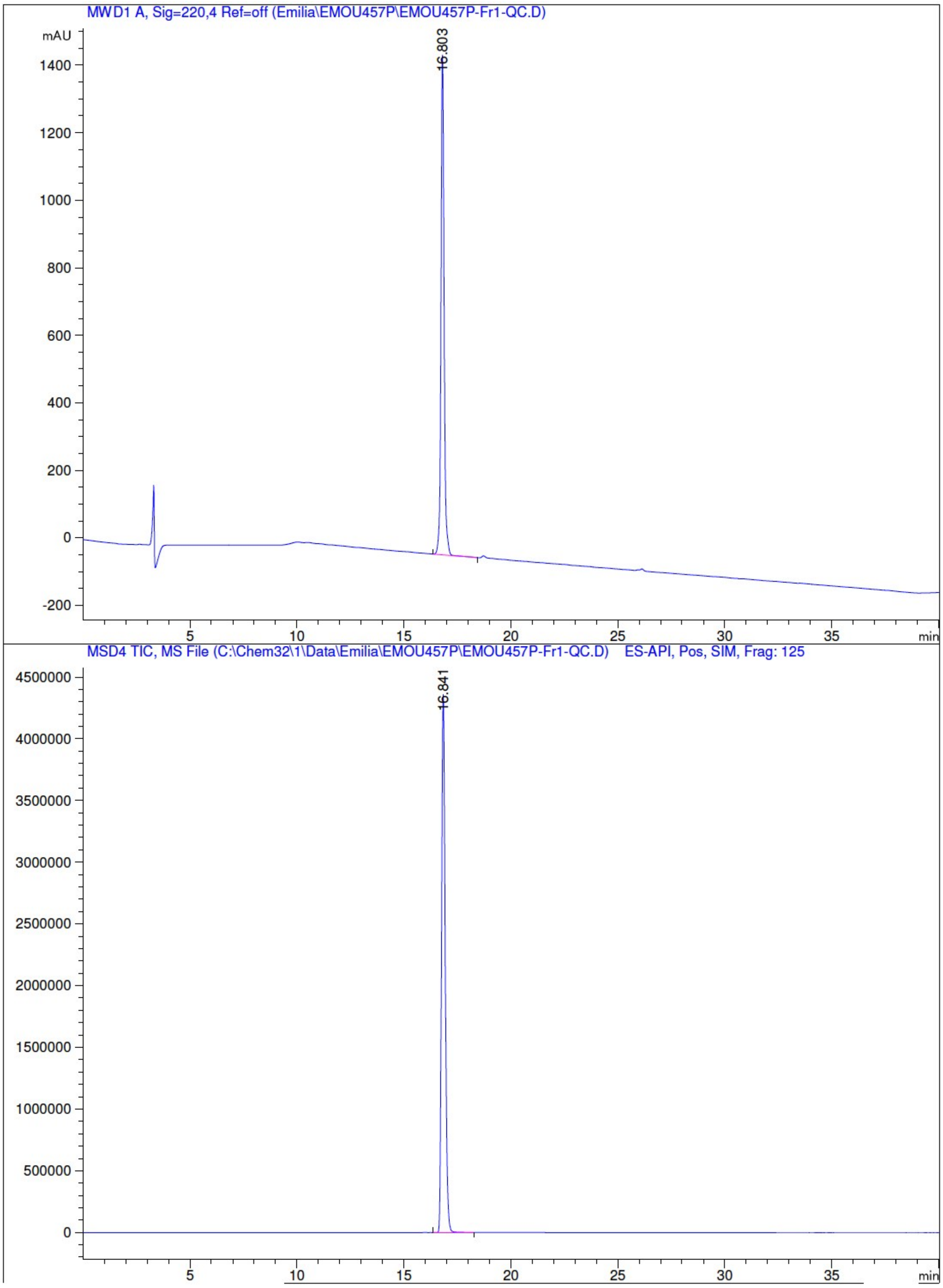

Peptide **4**; VGAGAI**G*Tz*$_{1\text{-}5}$A**<u>AYD</u>-NH_2, system A1

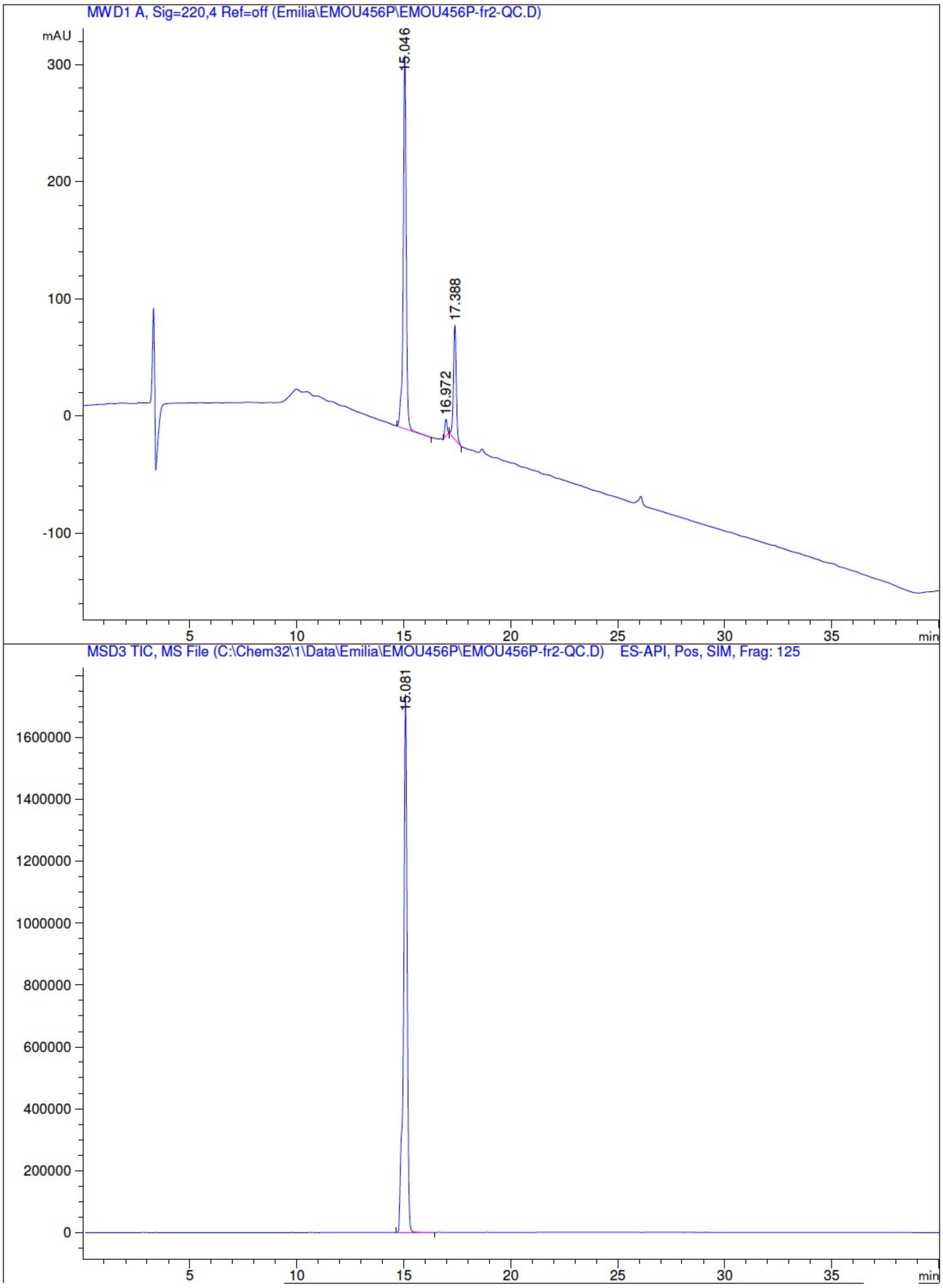

Peptide **5**; VGAGIGF**G*Tz***1-5**A**<u>AYD</u>-NH_2, system A1

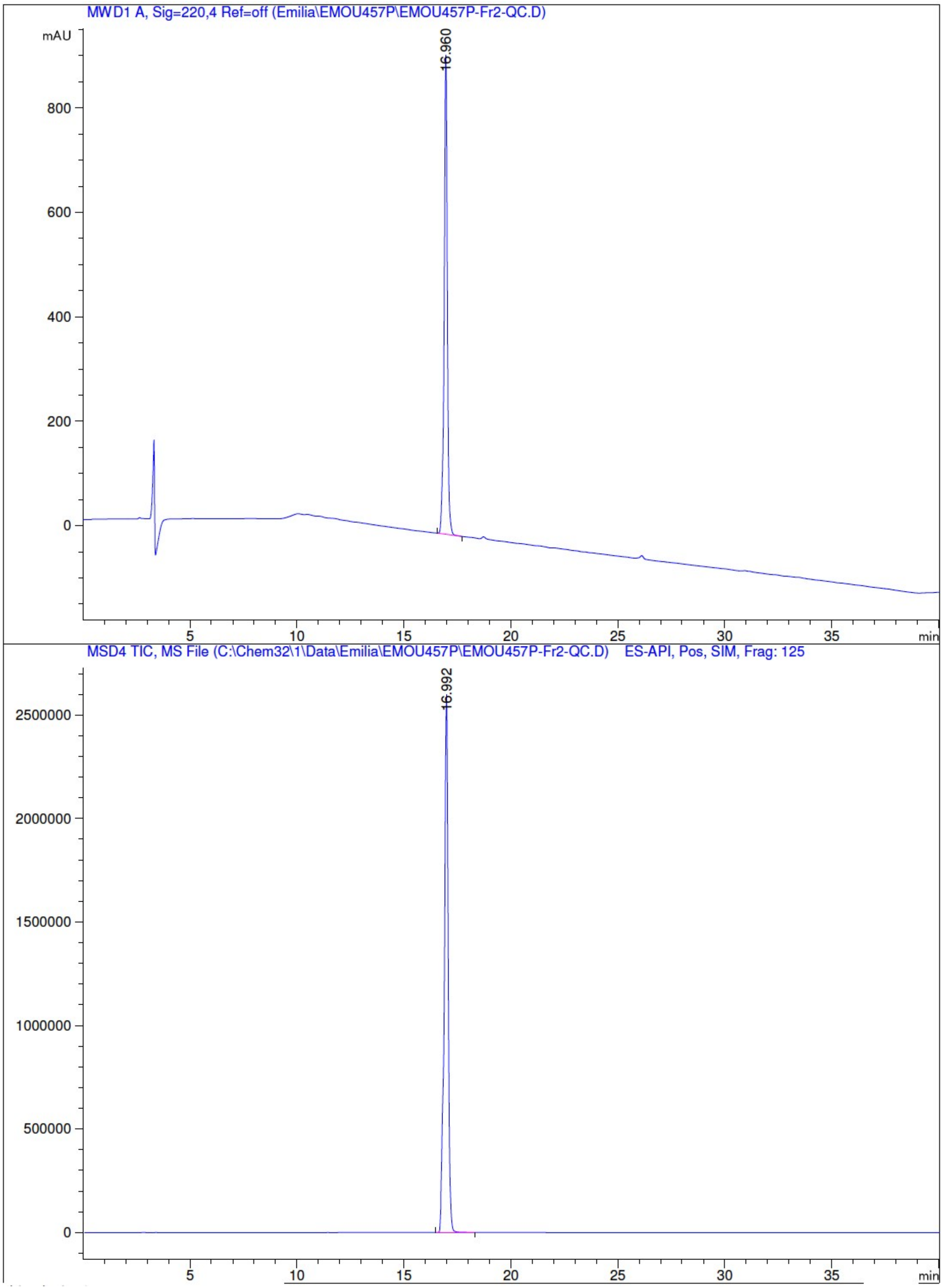

Peptide **6**; VGAGAI**GP**<u>AYD</u>-NH_2, system A1

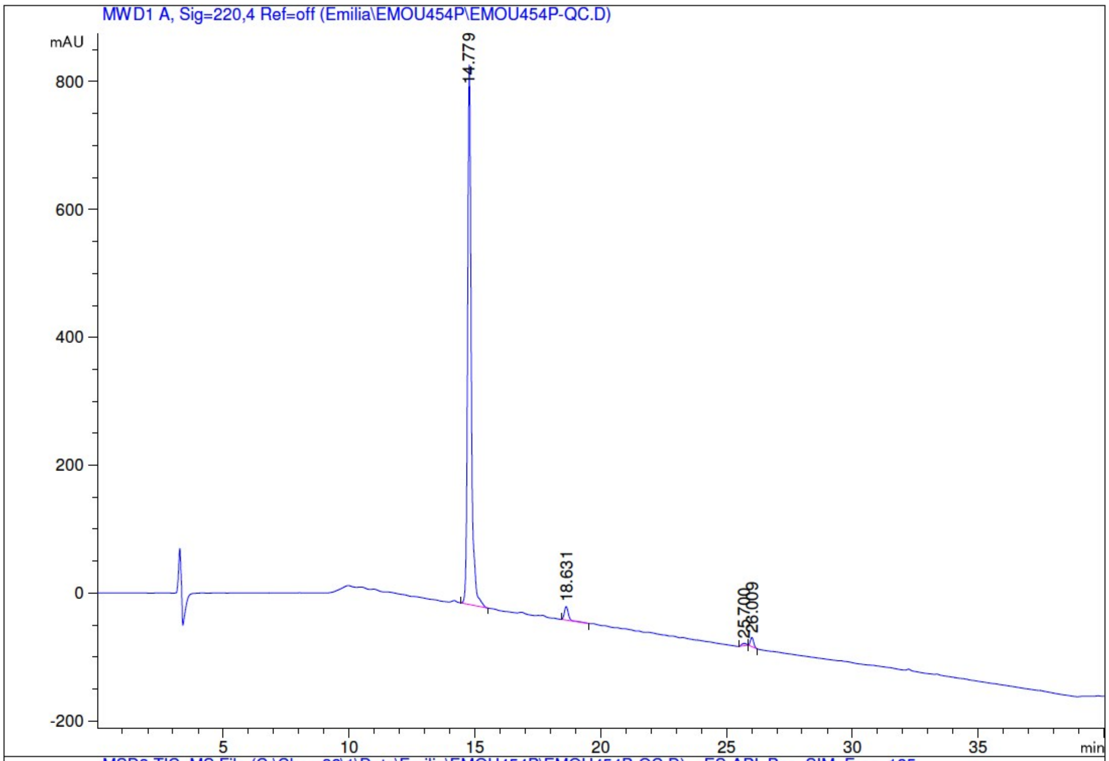

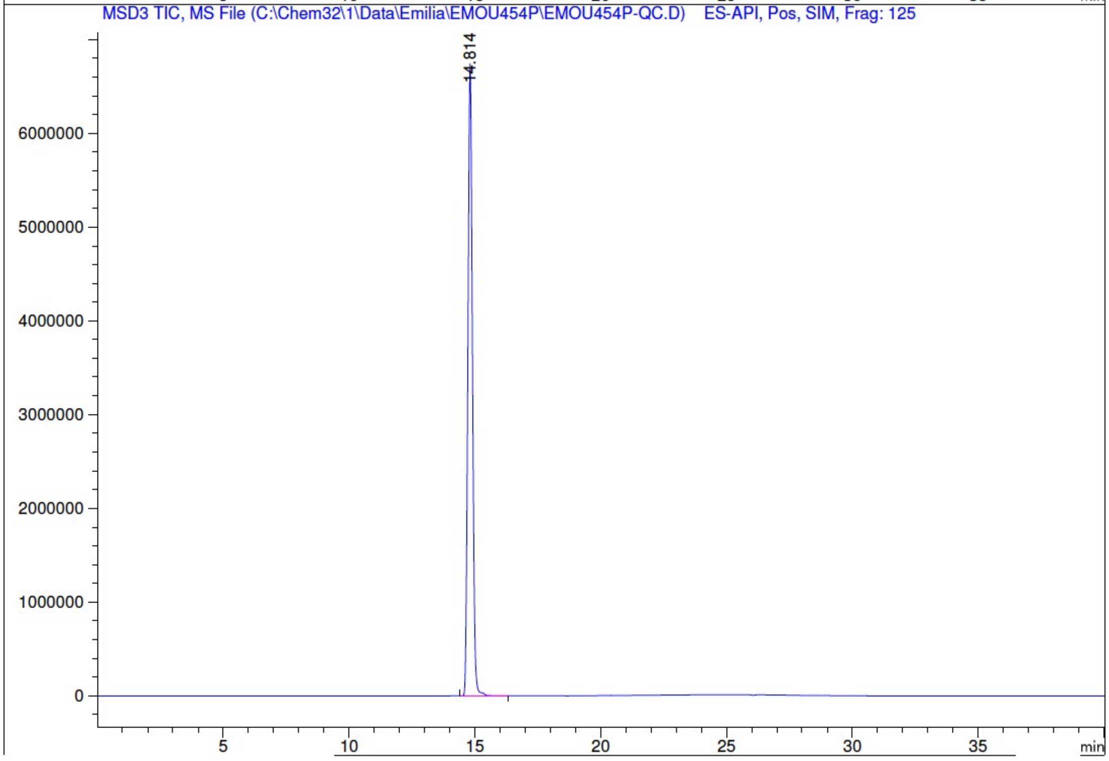

Peptide **7**; VGAGIG**AP**AYD-NH_2, system A1

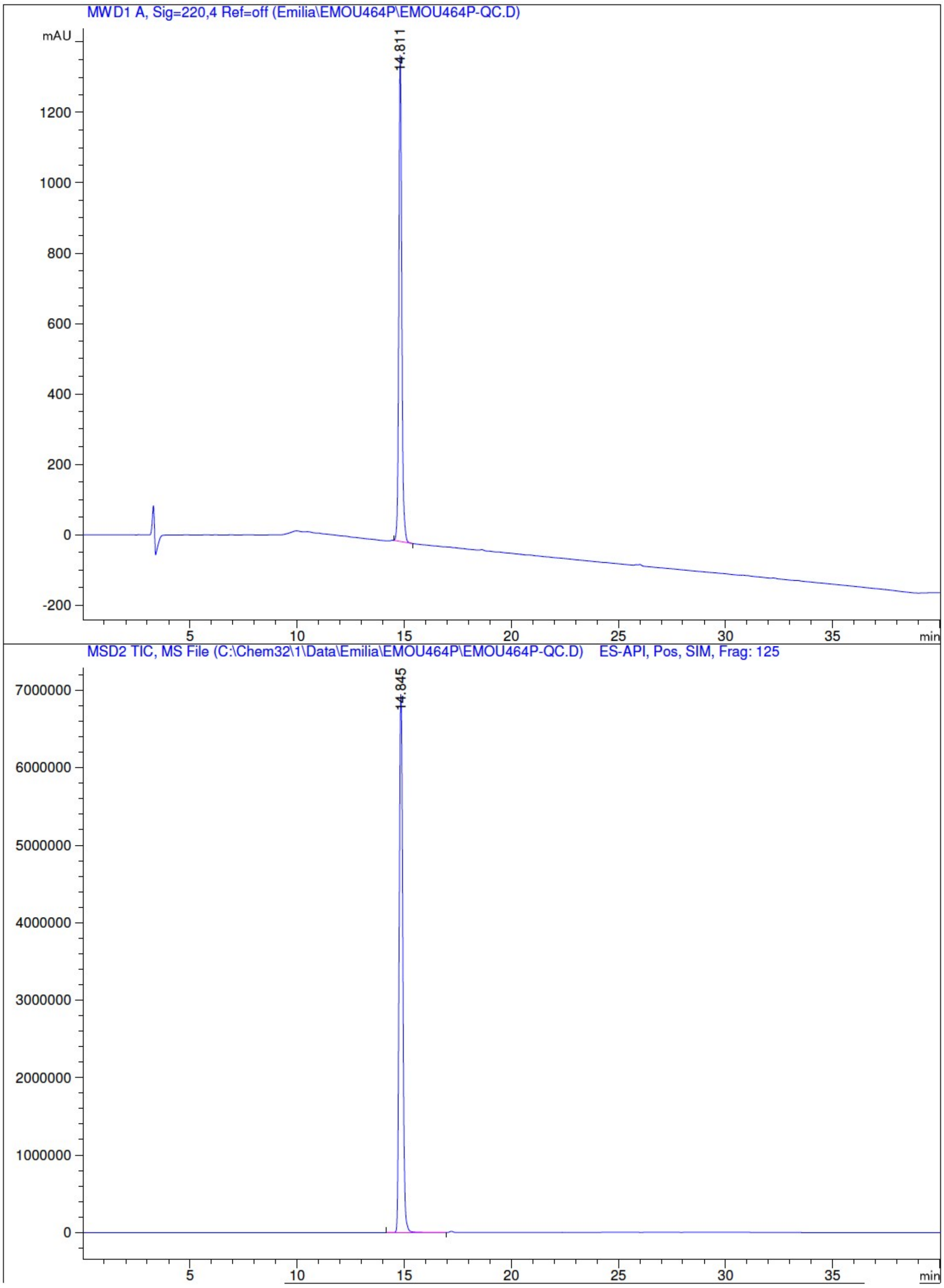

Peptide **9**; VGAGIG$_\beta$**AP**<u>AYD</u>-NH$_2$, system A1

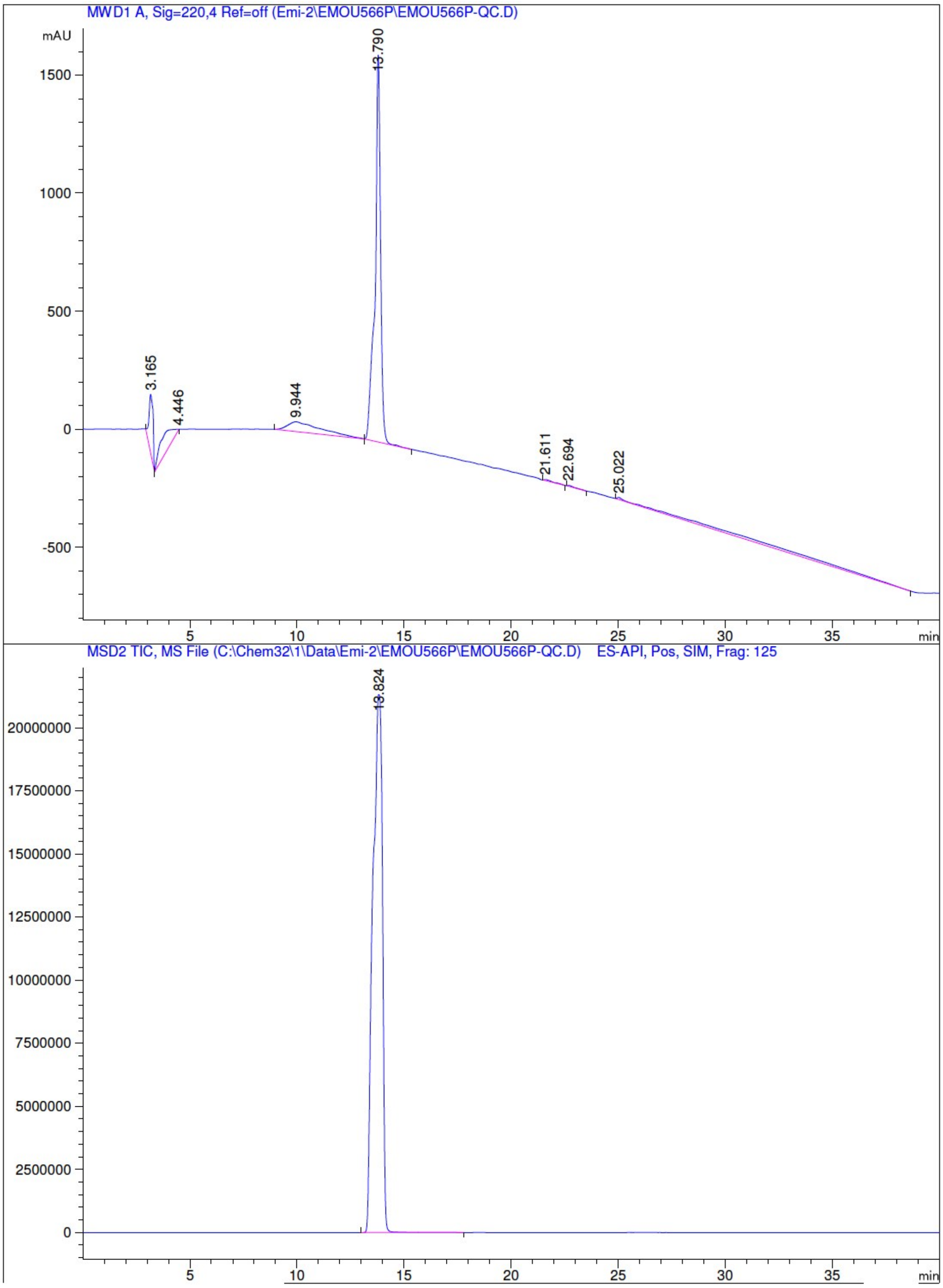

Peptide **10**; VGAGIG$_{\beta^{2}\text{-homo}}$**AP**AYD-NH$_2$, system A1

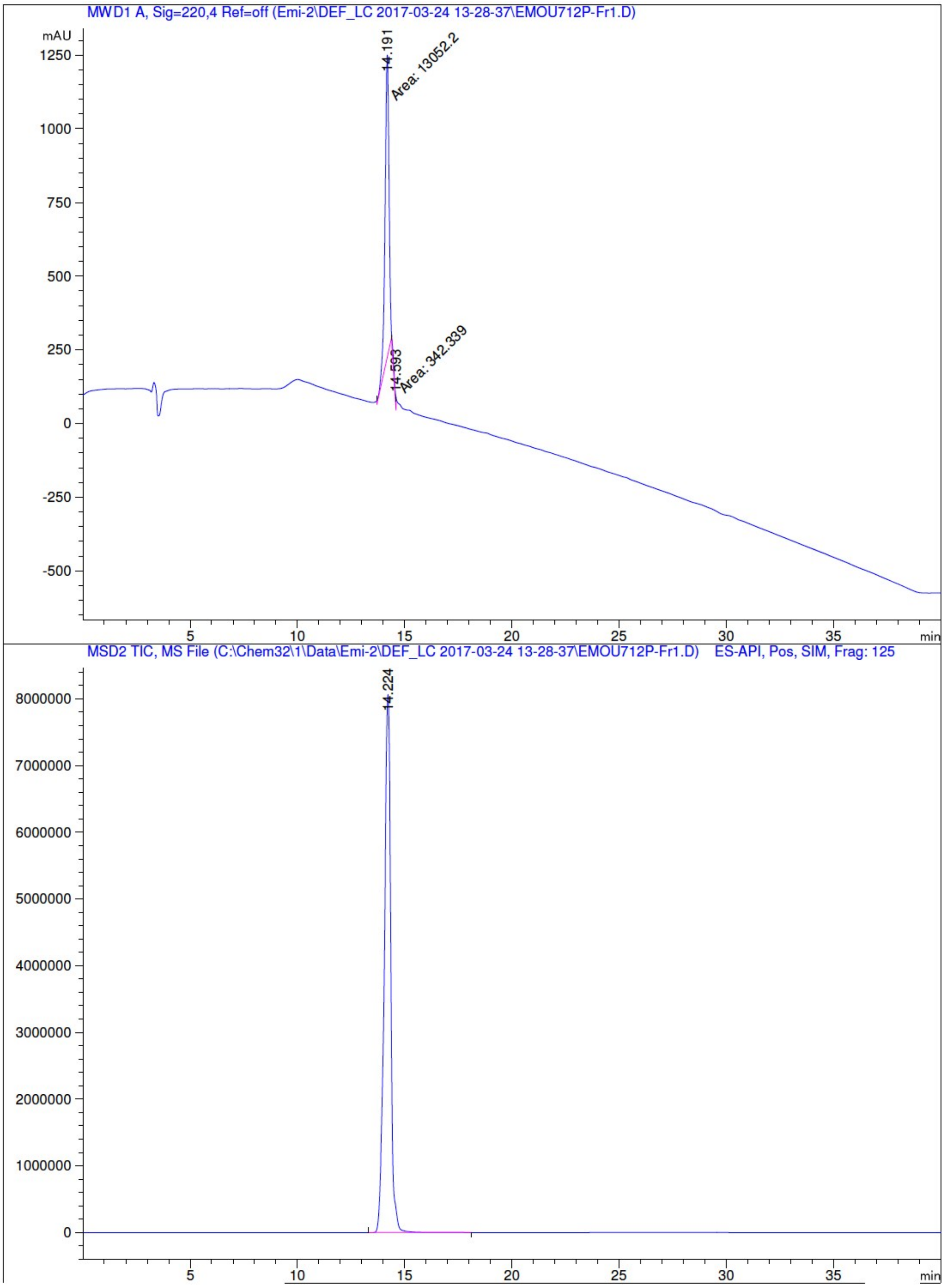

Peptide **11a**; VGAGIG**FTz**$_{1\text{-}4}$**A**<u>AYD</u>-NH_2, system A1

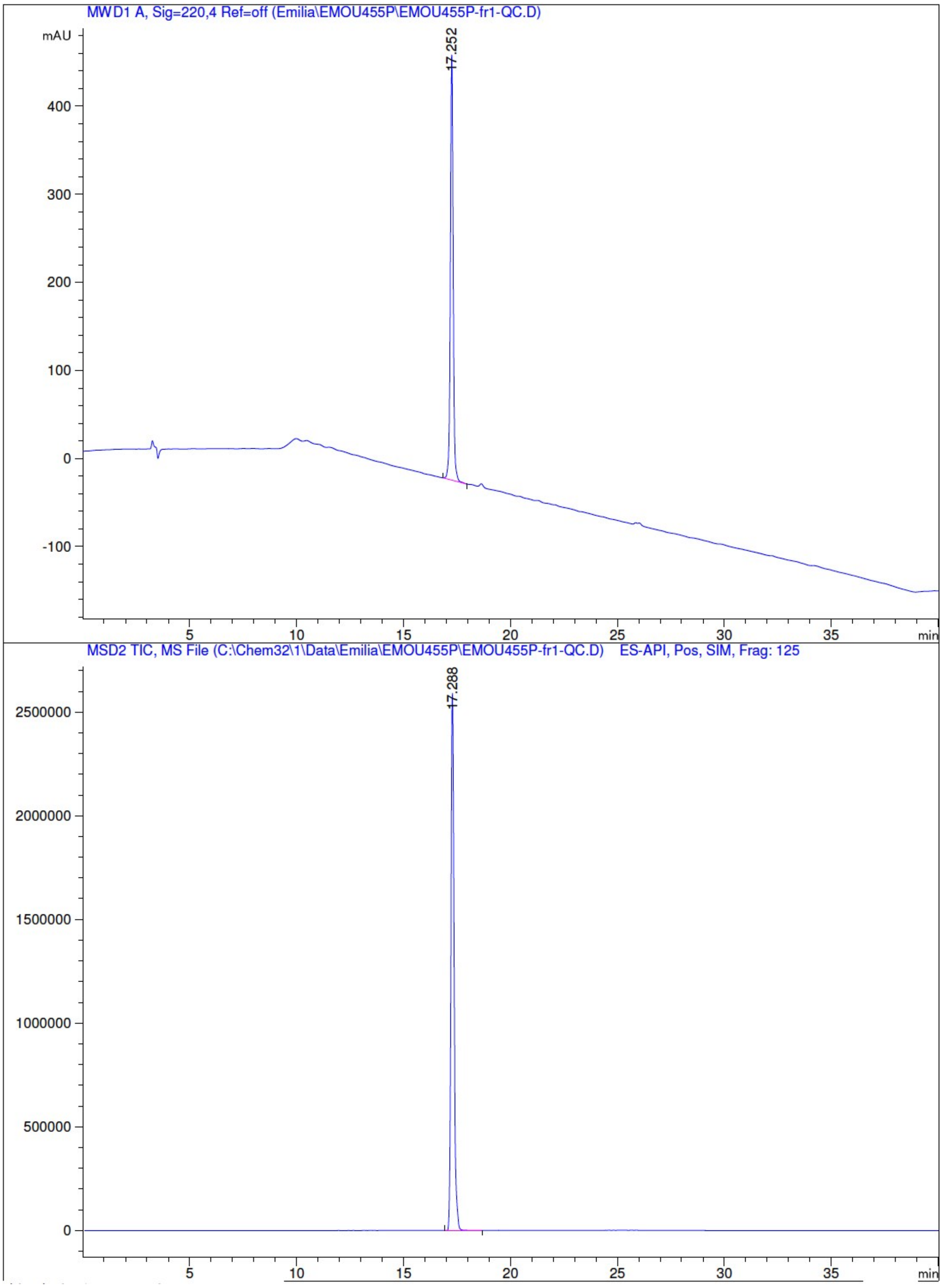

Peptide **11b**; VGAGIGf**Tz**$_{1\text{-}4}$**A**AYD-NH_2, system A1

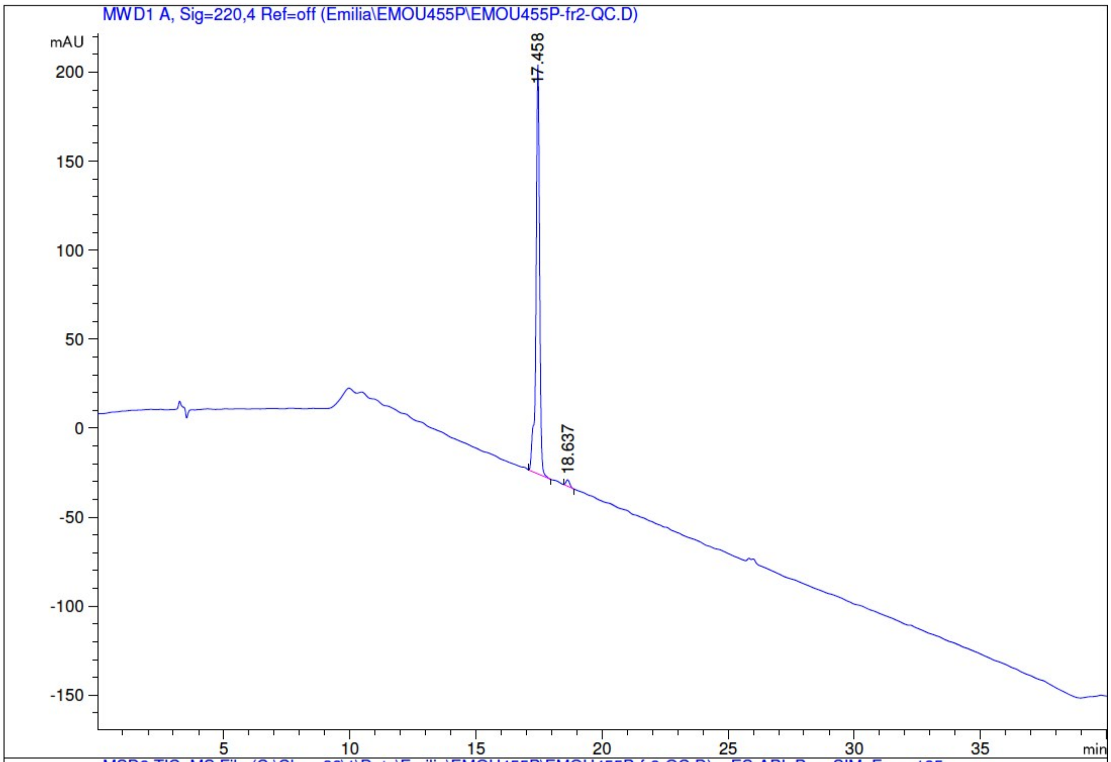

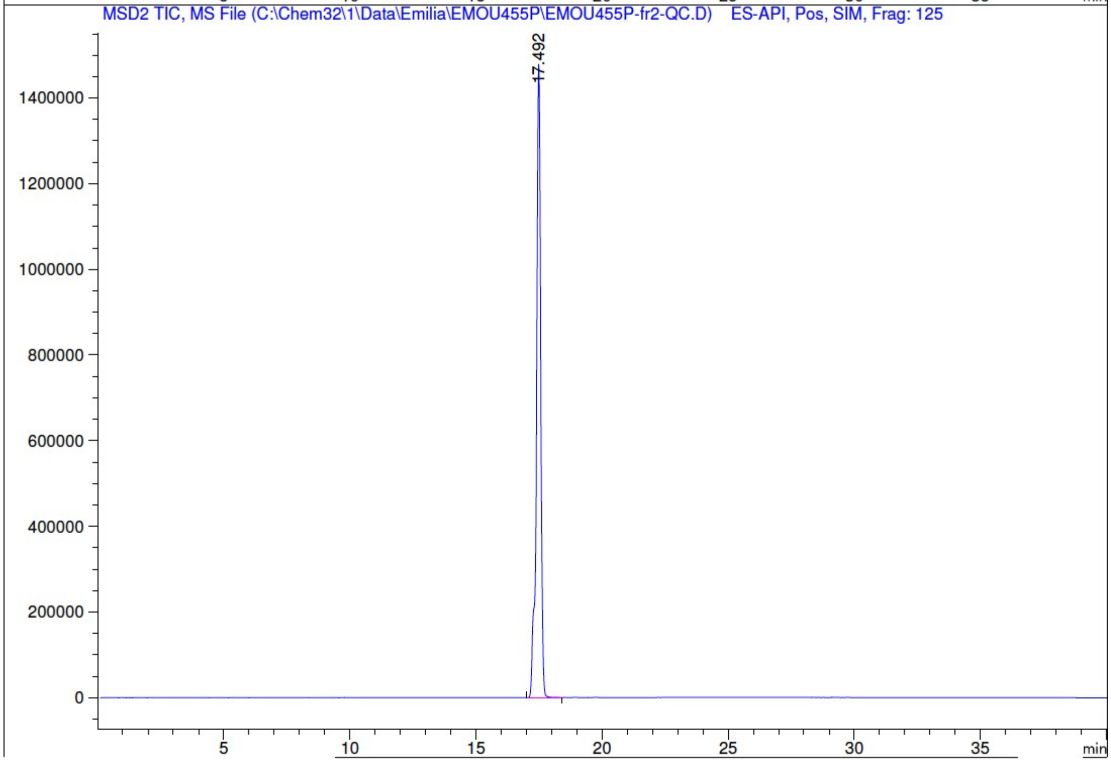

Peptide 1**2**; VGAGIG**f****P**AYD-NH_2, system A1

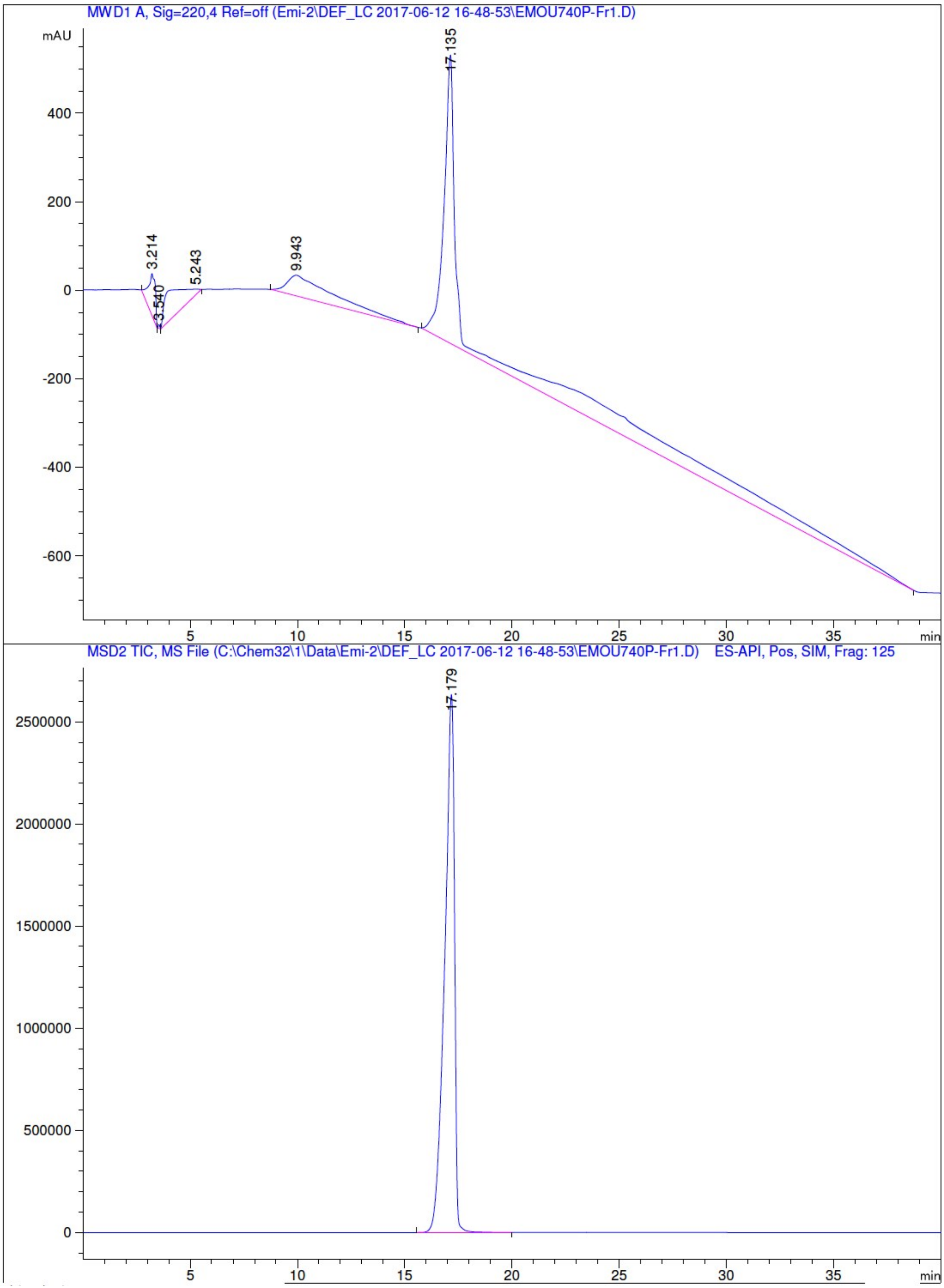

Peptide **13a**; VGAGIG**FTz**$_{1-4}$**G**AYD-NH_2, system A1

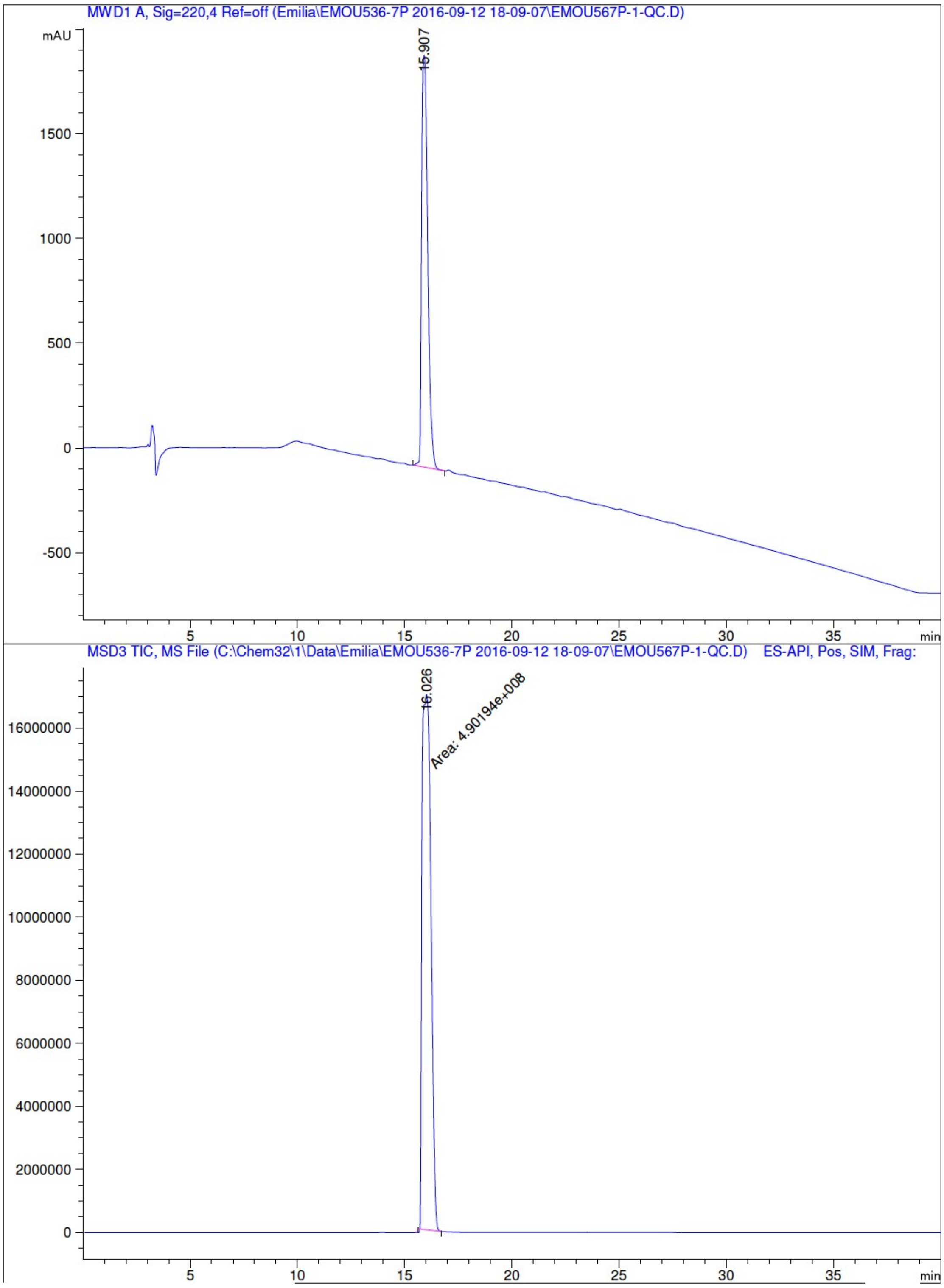

Peptide **13b**; VGAGIGf**Tz**$_{1-4}$**G**AYD-NH_2, system A1

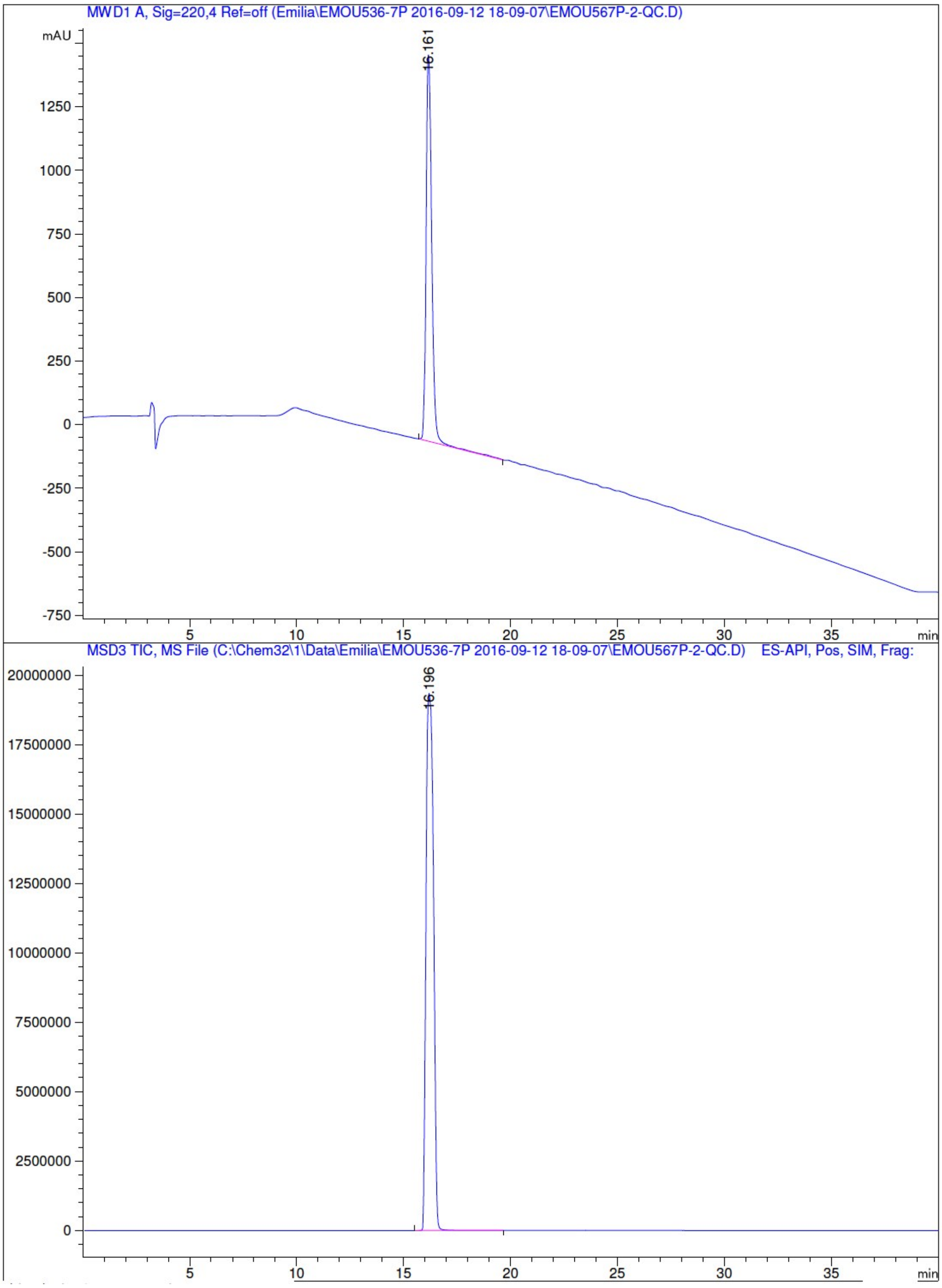

Peptide **14**; VGAGIGA**Tz**$_{1\text{-}4}$**A**AYD-NH$_2$, system A1

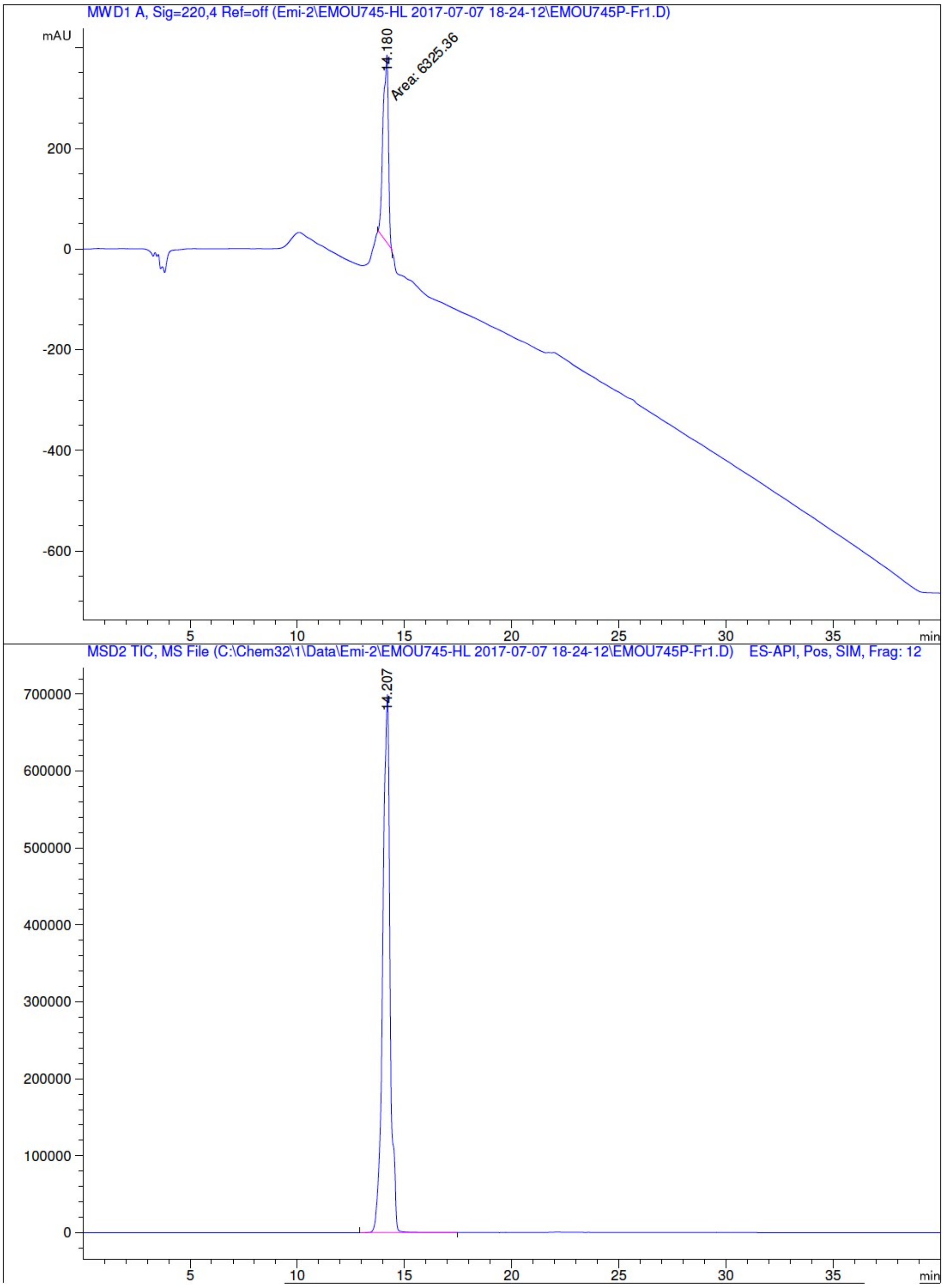

Peptide **15**; ITA**Tz**$_{1\text{-}4}$**A**IT**F****Tz**$_{1\text{-}4}$**A**AYD-NH_2, system A1

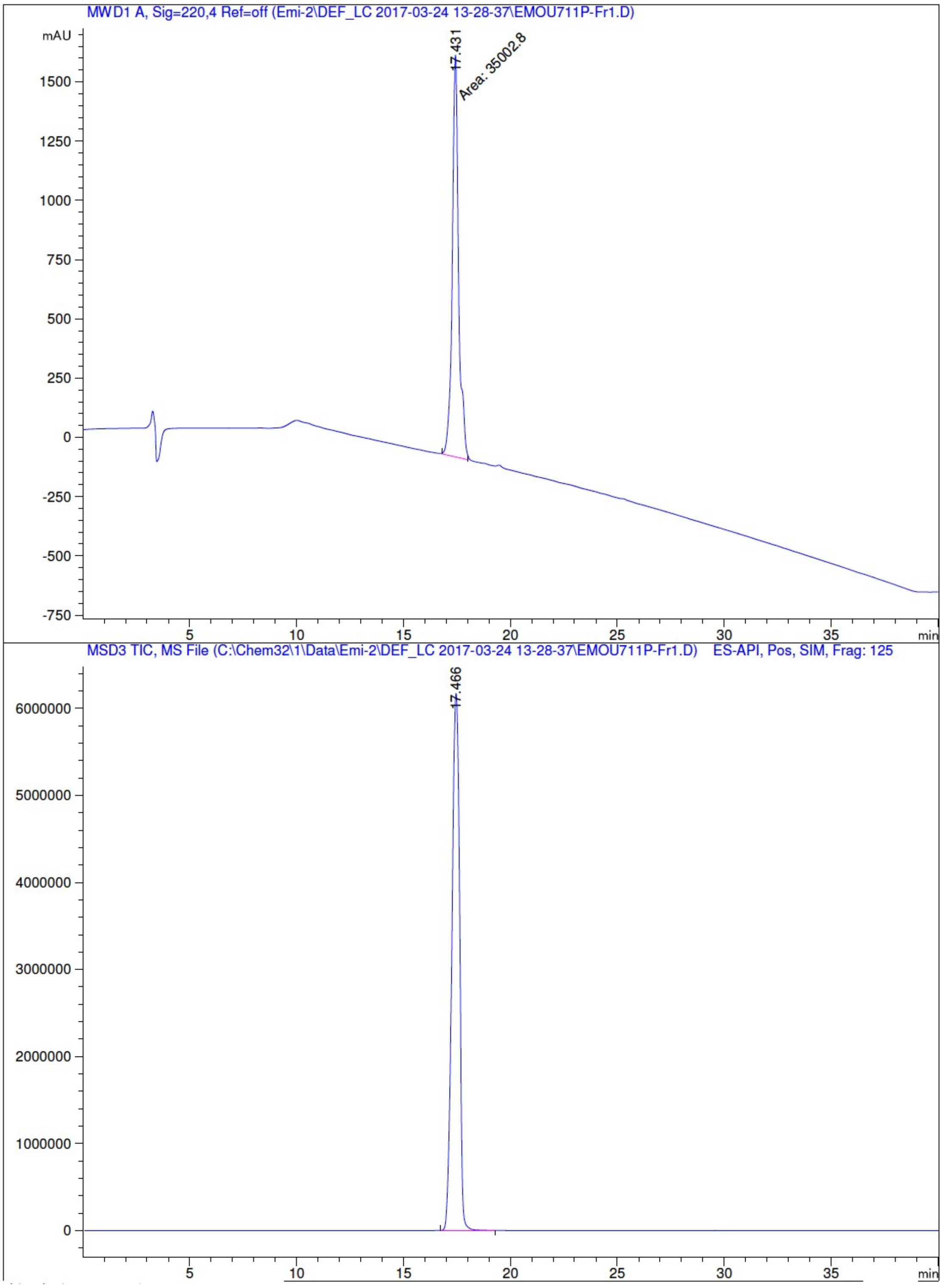

Peptide **16**; V-PEG$_4$-**FTz**$_{1\text{-}4}$**A**AYD-NH$_2$, system A1

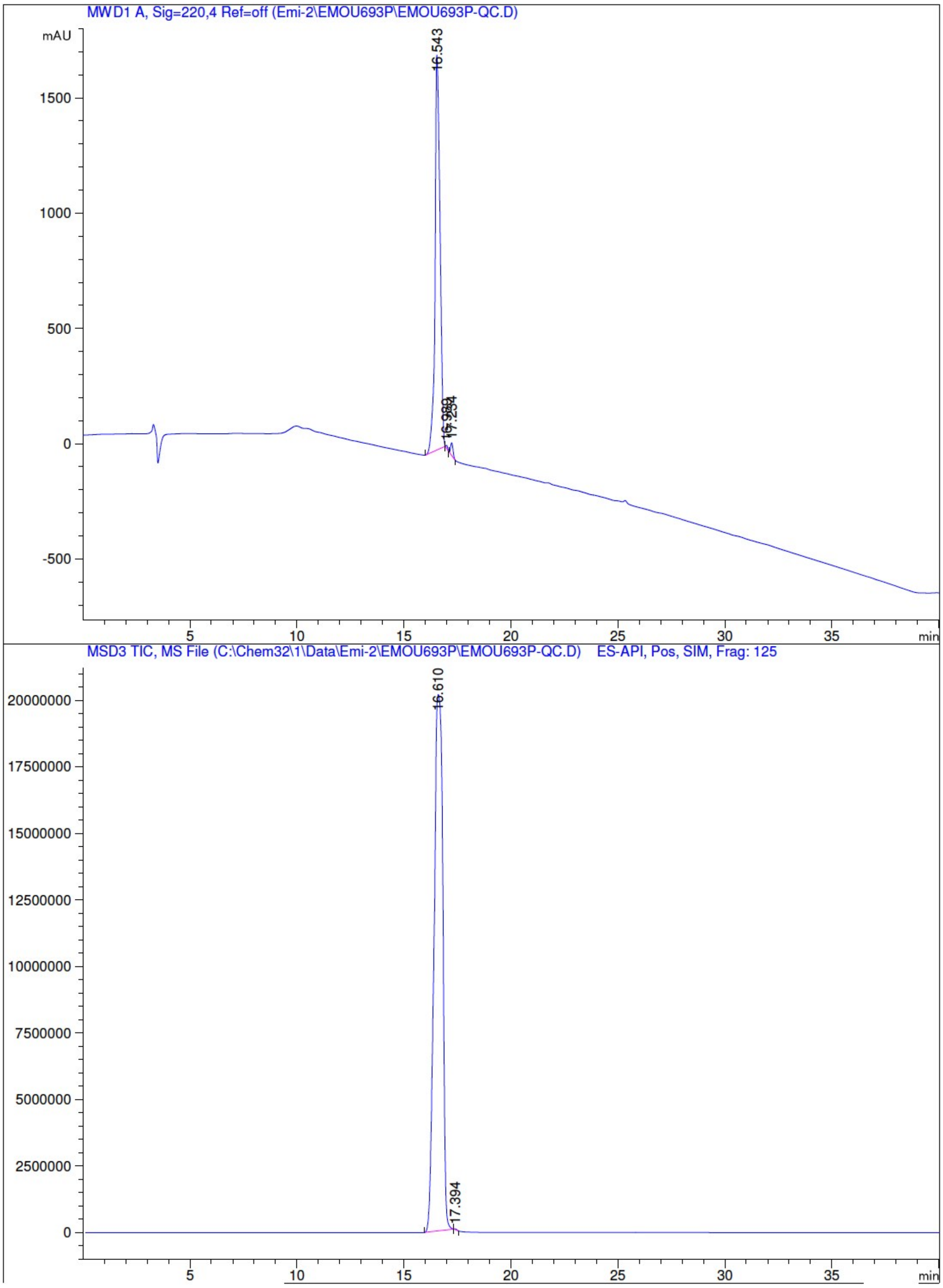

Peptide **17**; PEG_4-**F****Tz**$_{1\text{-}4}$**A**<u>AYD</u>-NH_2, system A1

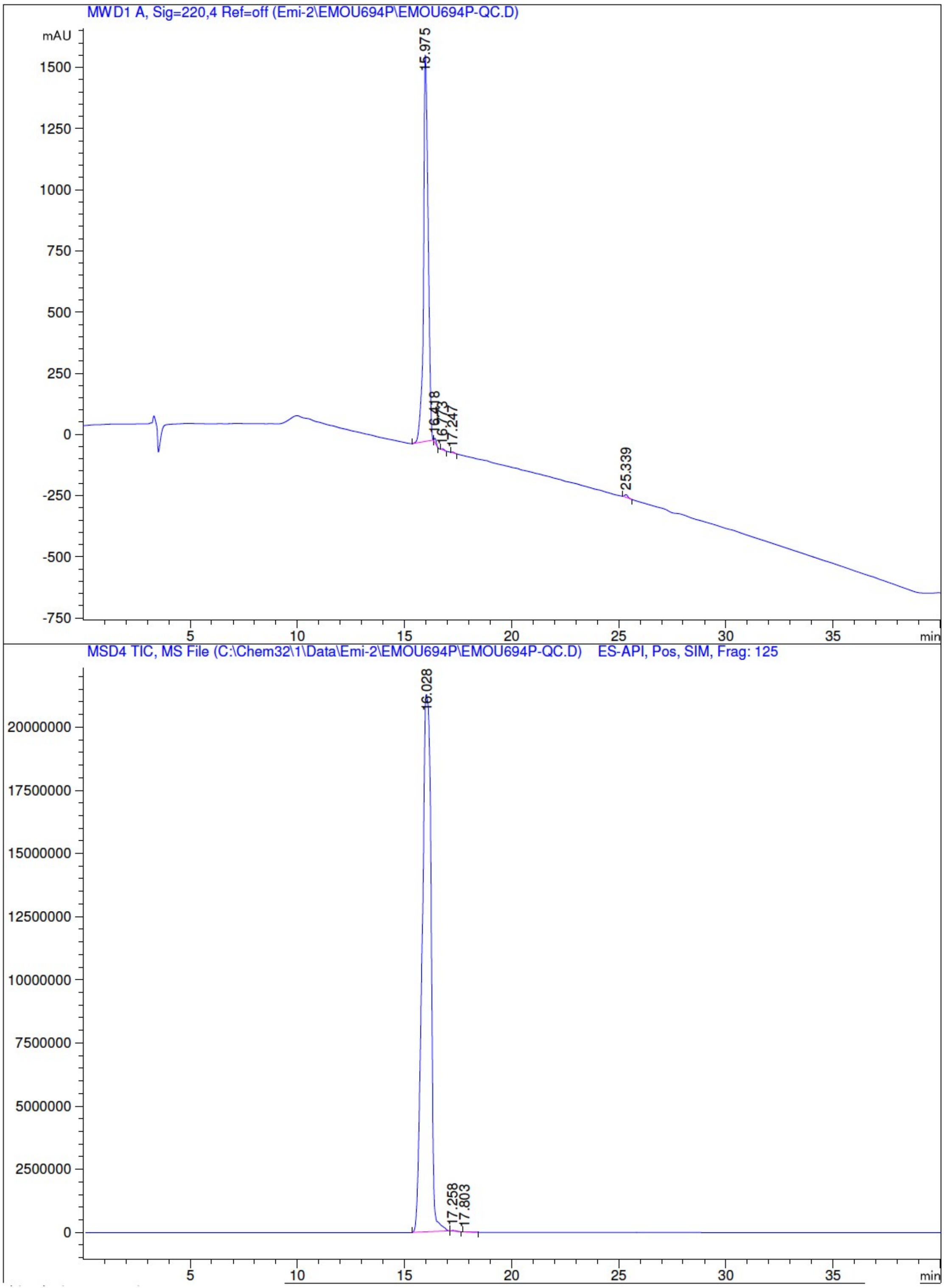

Peptide **18**; PEG_4-**ATz**$_{1\text{-}4}$**A**<u>AYD AYD</u>-NH_2, system A1

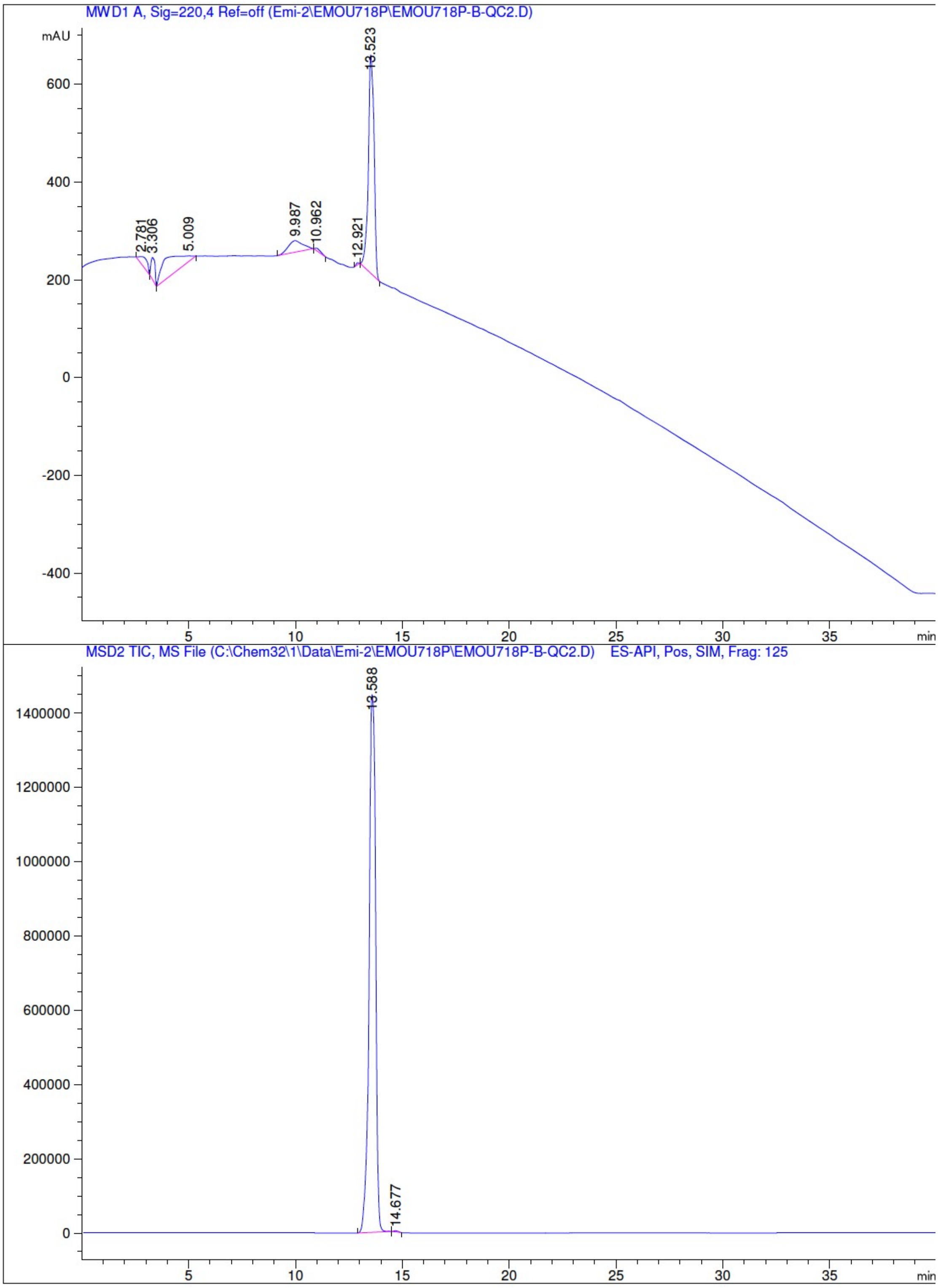

Peptide **19**; VGAGIF[**CC**]AYD-NH_2, system A1

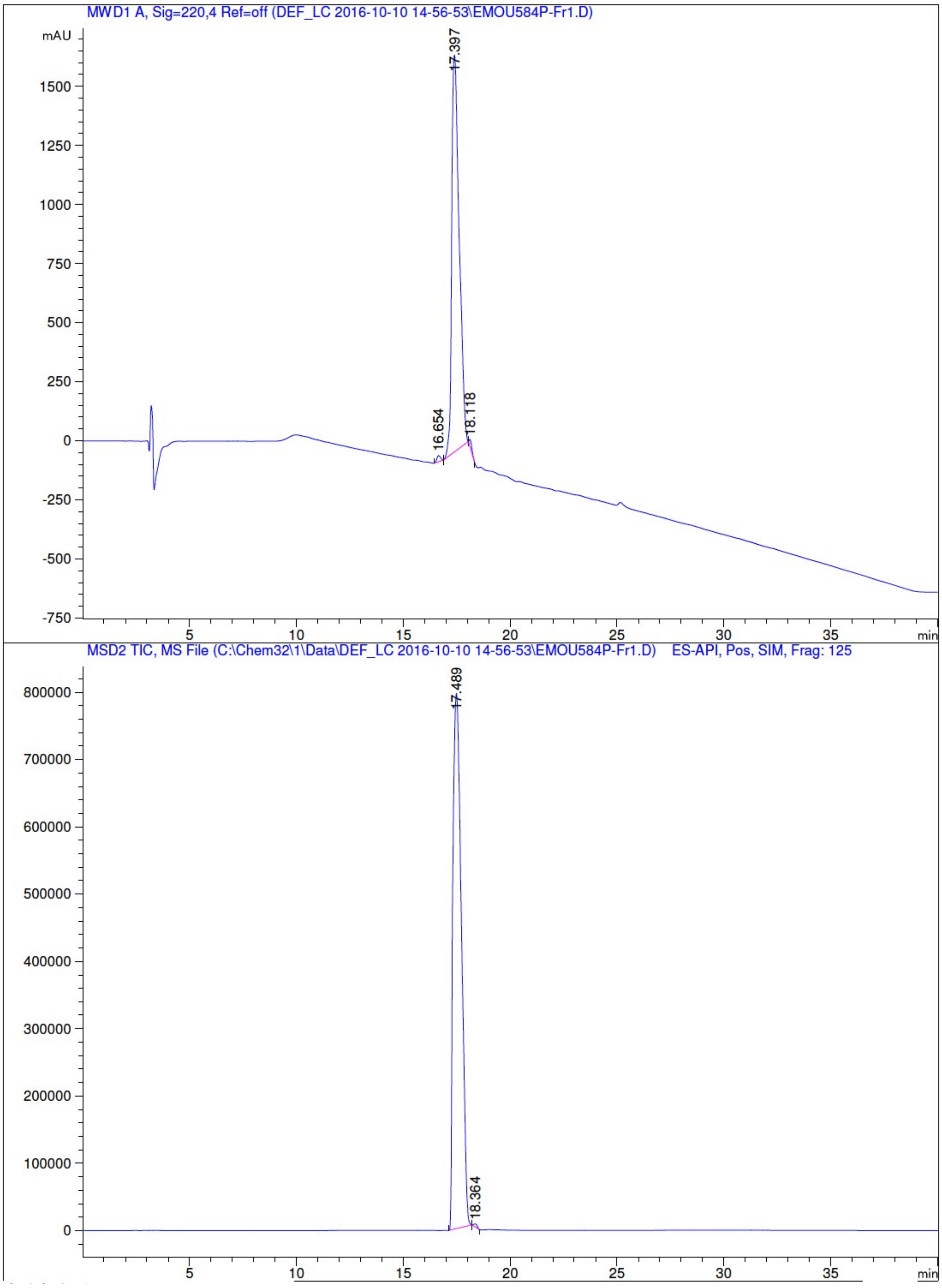

Peptide **20**; VPAPIP[**CC**]AYD-NH_2, system A1

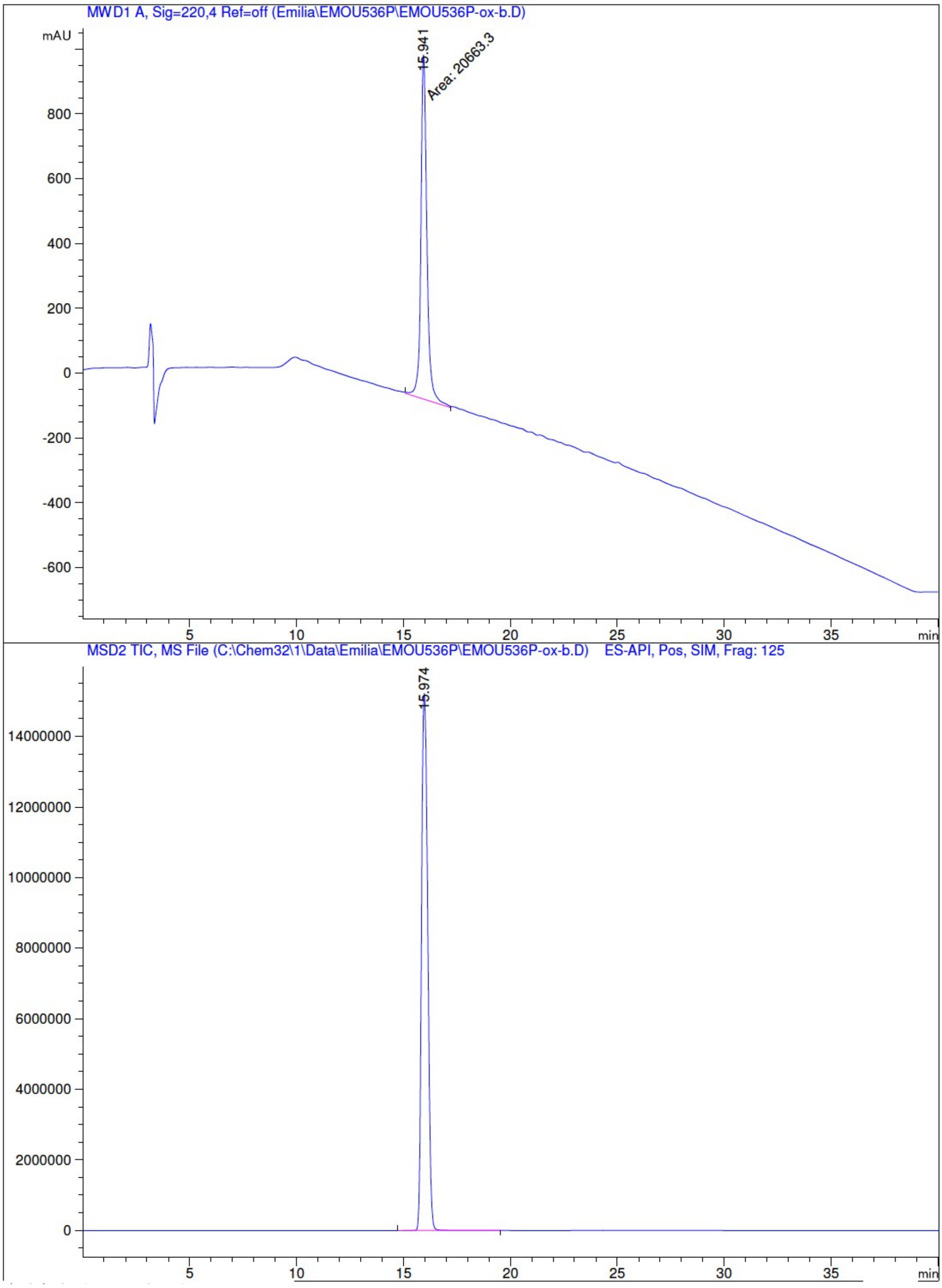

Peptide **21**; VGAGIF[**cc**]AYD-NH_2, system A1

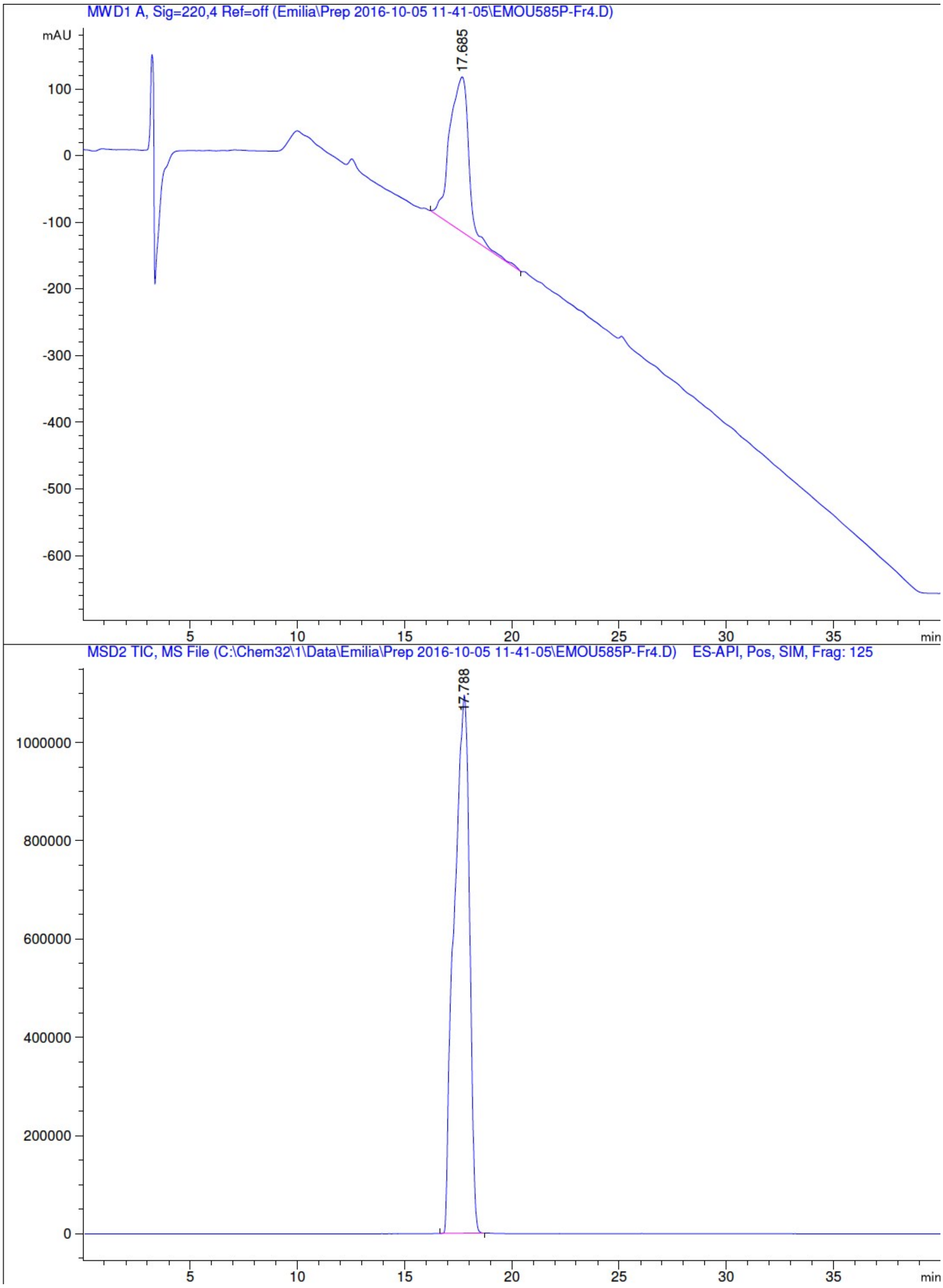

Peptide **22**; FyKT[**CC**]AYD-NH$_2$, system A1

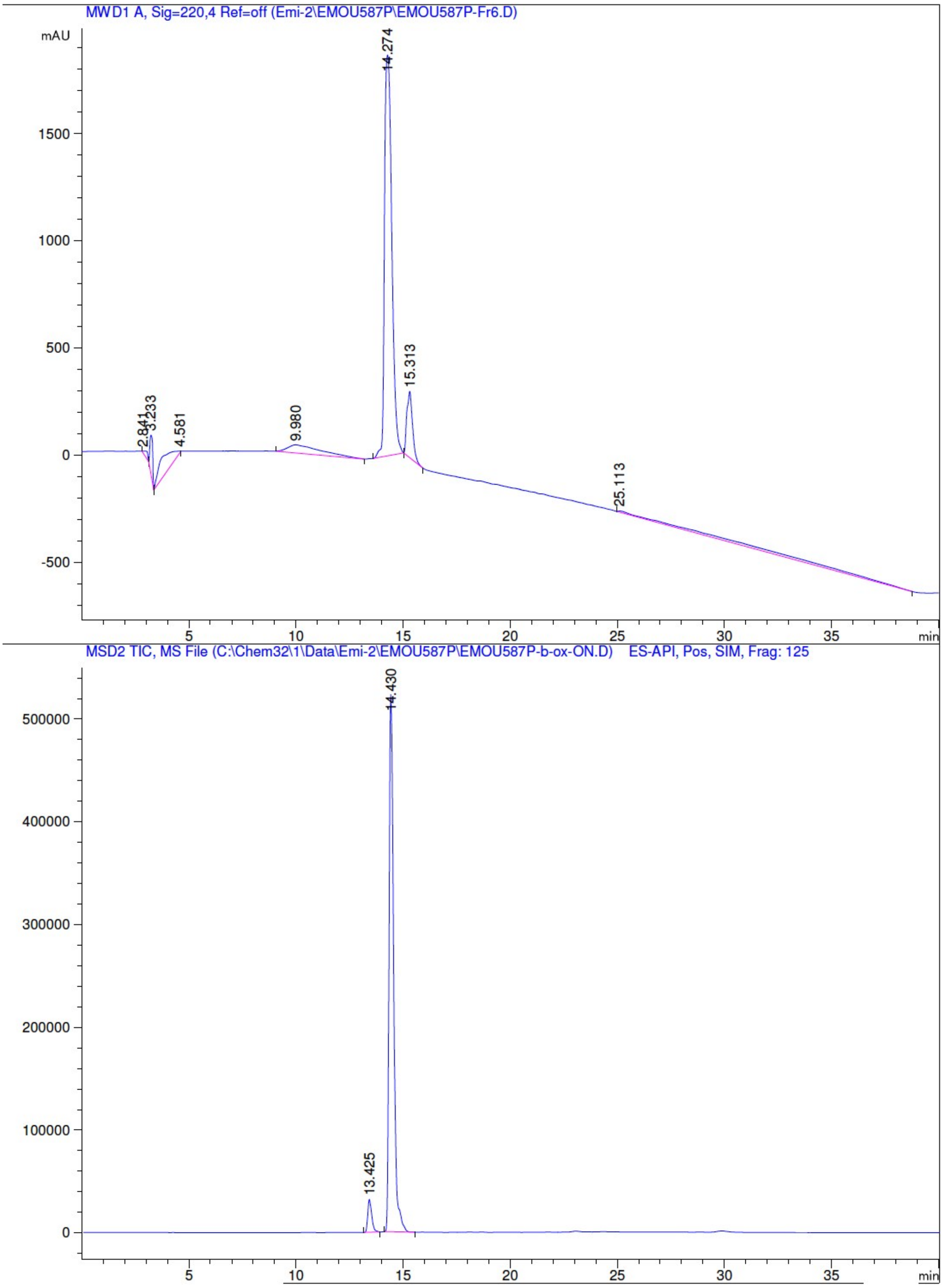

Peptide **23**; LKYG[**CC**]AYD-NH_2, system A1

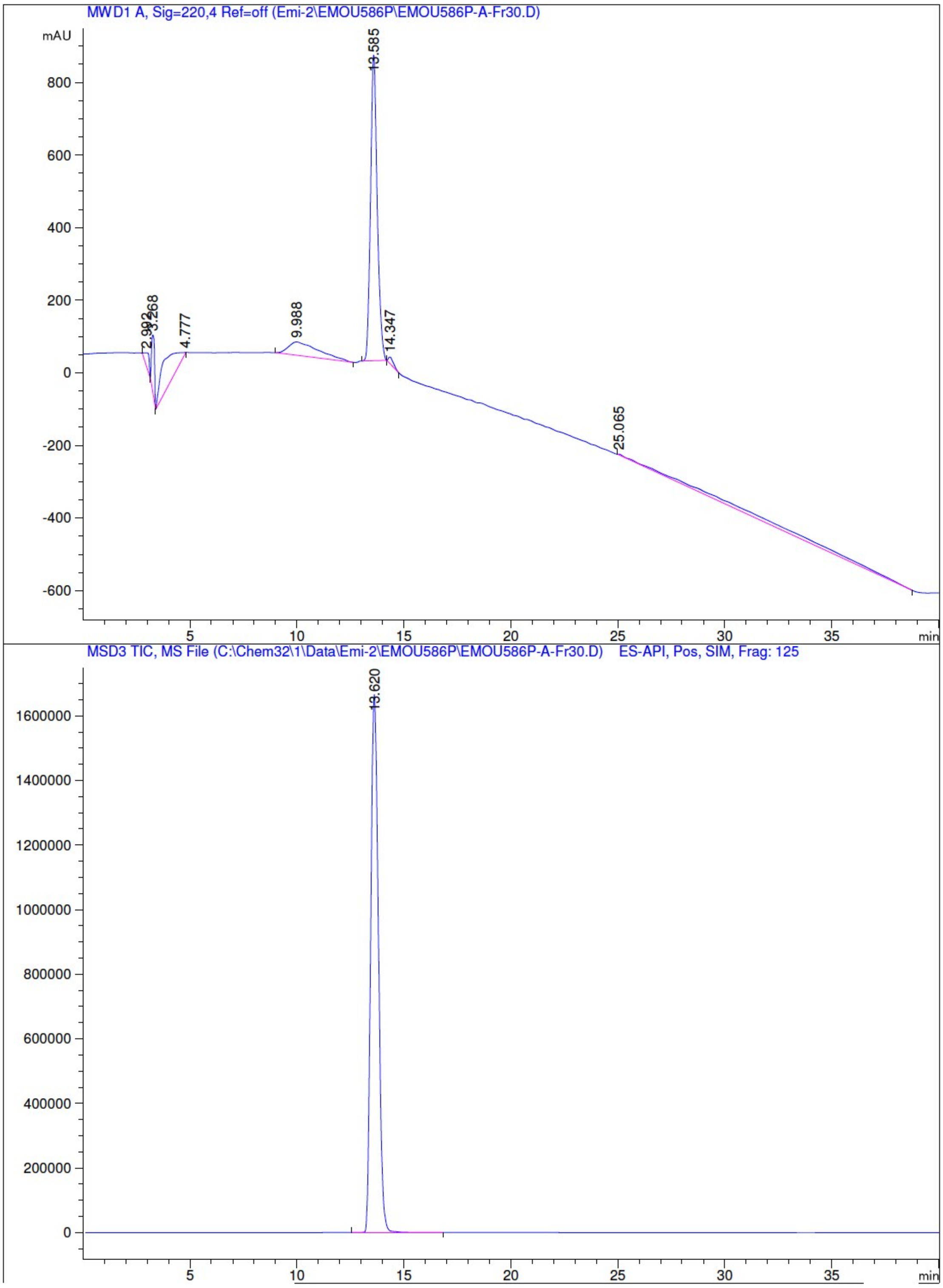

Peptide **24**; LGKYG[**CC**]AYD-NH_2, system A1

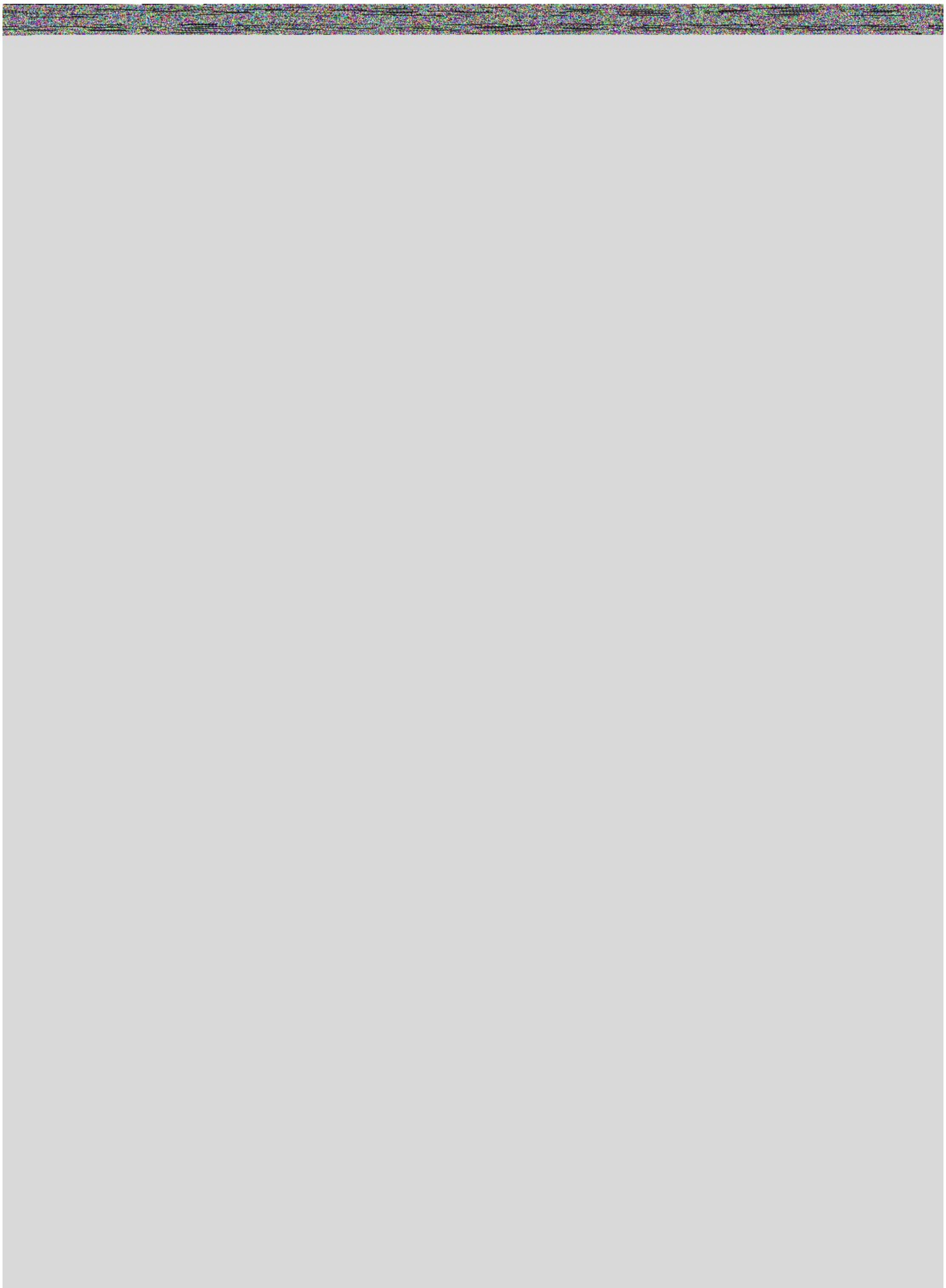

Peptide **25**; GYKLG[**CC**]AYD-NH_2, system A1

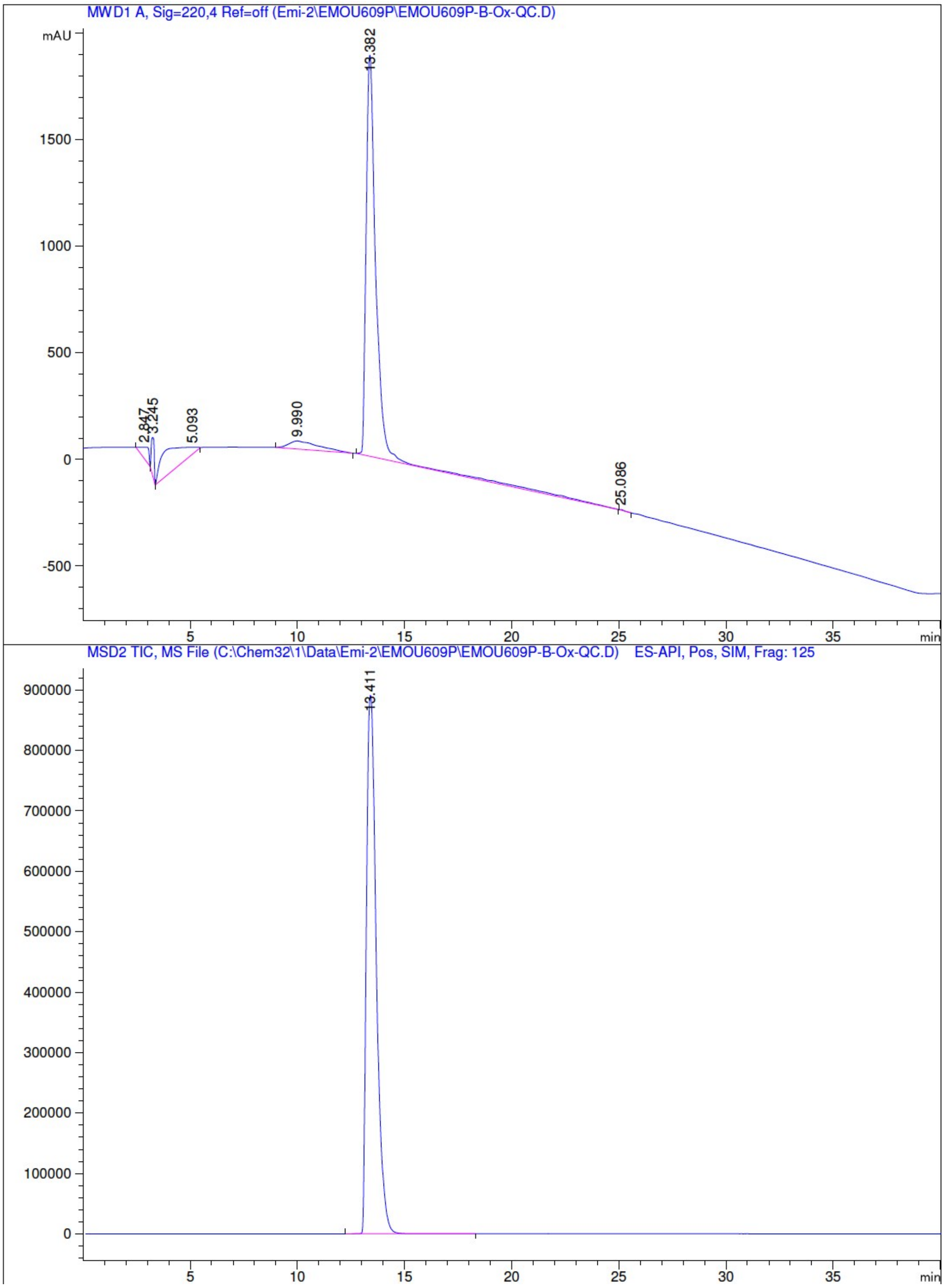

Peptide **26**; LGYKLG[**CC**]AYD-NH_2, system A1

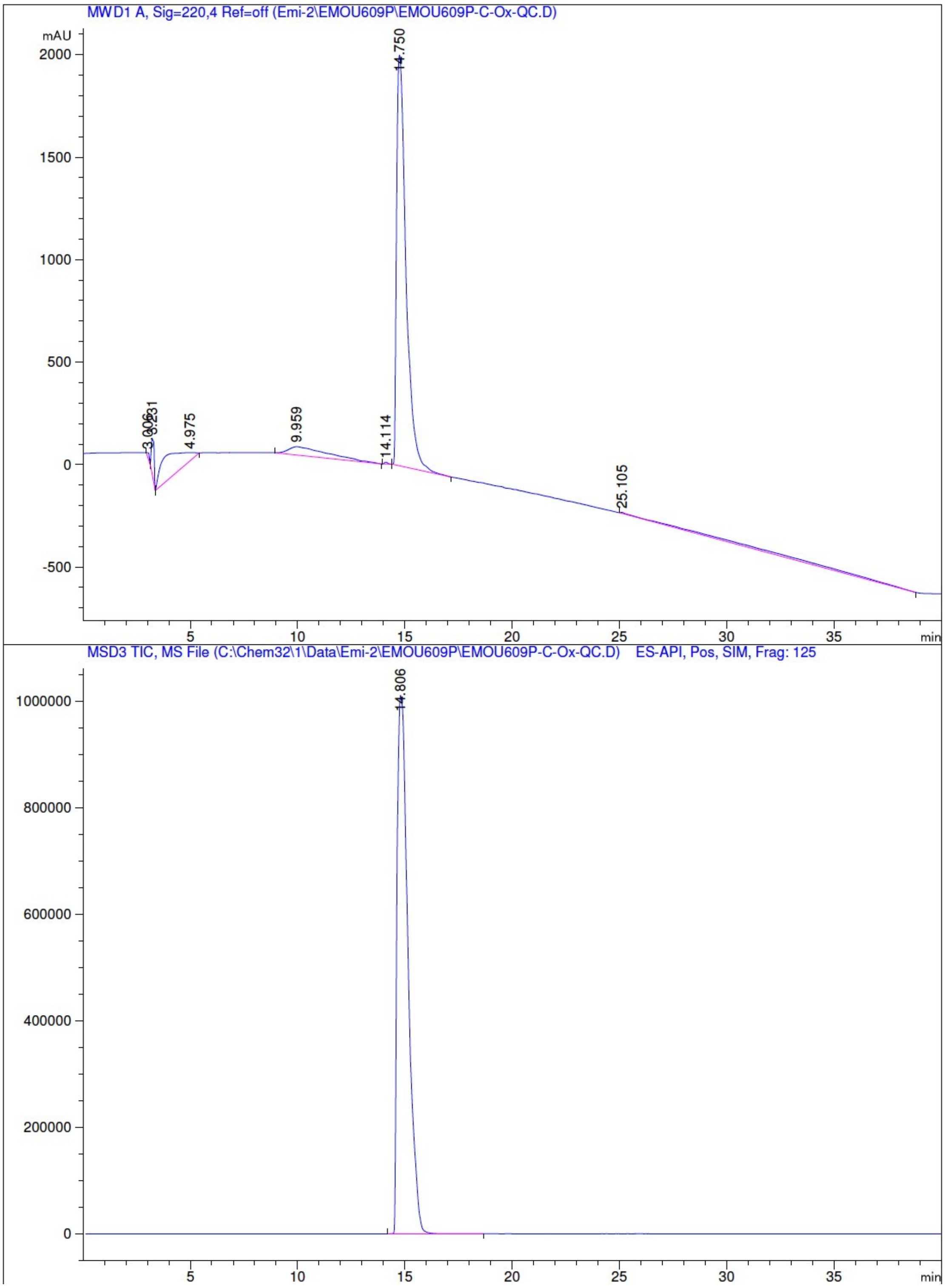

X. References

1- G. R. Fulmer, A. J. M. Miller, N. H. Sherden, H. E. Gottlieb, A. Nudelman, B. M. Stoltz, J. E. Bercaw and K. I. Goldberg, *Organometallics*, 2010, **29**, -2179.

2- J. Koehnke, A. Bent, W. E. Houssen, D. Zollman, F. Morawitz, S. Shirran, J. Vendome, A. F. Nneoyiegbe, L. Trembleau, C. H. Botting, M. C. M. Smith, M. Jaspars and J. H. Naismith, *Nat. Struct. Mol. Biol.*, 2012, **19**, -772.

3- P. Danner, M. Bauer, P. Phukan and Martin E. Maier, *European Journal of Organic Chemistry*, 2005, **2005**.

4- E. D. Goddard-Borger and R. V. Stick, *Org. Lett.*, 2007, **9**, -3800.

5- Lundquist and J. C. Pelletier, *Org. Lett.*, 2001, **3**, -783.

6- A. Tam, U. Arnold, M. B. Soellner and R. T. Raines, *J. Am. Chem. Soc.*, 2007, **129**, -12671.

7- E. Oueis, M. Jaspars, N. J. Westwood and J. H. Naismith, *Angew. Chem. Int. Ed.*, 2016, **55**.
